# Supplementary material for: Trial summary and protocol for a phase II randomised placebo-controlled double-blinded trial of Interleukin 1 blockade in Acute Severe Colitis: the IASO trial
Source: BMJ Open. 2019 Feb 15;9(2):e023765. doi: 10.1136/bmjopen-2018-023765 (PMC6398753; doi:10.1136/bmjopen-2018-023765)
Supplement: Supplementary file 1 [file bmjopen-2018-023765supp001.pdf]

Modified Truelove Witts Severity Index Form

Information for the IASO Trial

Subject ID: N ☐ ☐ - 1 ☐ ☐ ☐ IASO Trial Day: ________

Subject Date of Birth: D D - M M M - Y Y Y Y

Date of completion: D D - M M M - Y Y Y Y

Symptom Checklist (Circle the score that applies)

Category	Score
Diarrhoea (Number of daily stools)	
0–2	0
3–4	1
5–6	2
7–9	3
≥10	4
Nocturnal diarrhoea	
No	0
Yes	1
Visible blood in stool (% of movements)	
0%	0
<50%	1
≥50%	2
100%	3
Faecal incontinence	
No	0
Yes	1
Abdominal pain or cramping	
None	0
Mild	1
Moderate	2
Severe	3

Further categories are included overleaf

Modified Truelove Witts Severity Index Form

Category	Score
General well-being	
Perfect	0
Very good	1
Good	2
Average	3
Poor	4
Terrible	5
Abdominal tenderness	
None	0
Mild and localised	1
Mild to moderate & diffuse	2
Severe or rebound	3
Need for antidiarrhoeal drugs	
No	0
Yes	1

Total Score (Add all the circled scores together)

Total MTWSI score (between 0–21): ☐
