# Supplementary material for: Trial summary and protocol for a phase II randomised placebo-controlled double-blinded trial of Interleukin 1 blockade in Acute Severe Colitis: the IASO trial
Source: BMJ Open. 2019 Feb 15;9(2):e023765. doi: 10.1136/bmjopen-2018-023765 (PMC6398753; doi:10.1136/bmjopen-2018-023765)
Supplement: Author's manuscript [file bmjopen-2018-023765.draft_revisions.pdf]

BMJ Open is committed to open peer review. As part of this commitment we make the peer review history of every article we publish publicly available.

When an article is published we post the peer reviewers' comments and the authors' responses online. We also post the versions of the paper that were used during peer review. These are the versions that the peer review comments apply to.

The versions of the paper that follow are the versions that were submitted during the peer review process. They are not the versions of record or the final published versions. They should not be cited or distributed as the published version of this manuscript.

BMJ Open is an open access journal and the full, final, typeset and author-corrected version of record of the manuscript is available on our site with no access controls, subscription charges or pay-per-view fees (http://bmjopen.bmj.com).

If you have any questions on BMJ Open's open peer review process please email info.bmjopen@bmj.com

BMJ Open

Trial Summary & Protocol for a phase II randomised placebo controlled double blinded trial of Interleukin 1 blockade in Acute Severe Colitis – the IASO Trial

Journal:	*BMJ Open*
Manuscript ID	bmjopen-2018-023765
Article Type:	Protocol
Date Submitted by the Author:	25-Apr-2018

Complete List of Authors:	Thomas, Martin ; Cambridge Clinical Trials Unit<br>Bayliss, Carrie; Cambridge Clinical Trials Unit<br>Bond, Simon; Cambridge Clinical Trials Unit; MRC Biostatistics Unit<br>Dowling, Francis; Cambridge Clinical Trials Unit<br>Galea, James; University of Manchester<br>Jairath, Vipul; Department of Medicine, Division of Gastroenterology, Western University; Division of Epidemiology and Biostatistics, Western University<br>Lamb, Christopher; Institute of Cellular Medicine, Newcastle University; Newcastle Upon Tyne Hospitals NHS Foundation Trust, Department of Gastroenterology<br>Probert, Chris; Institue of Translational Medicine, University of Liverpool<br>Timperley-Preece, Elizabeth<br>Watson, Alastair; University of East Anglia<br>Whitehead, Lynne; Addenbrooke's Hospital<br>Williams, John; Swansea University Medical School<br>Parkes, M; Addenbrooke's Hospital; Department of Medicine, University of Cambridge<br>Kaser, Arthur; Department of Medicine, University of Cambridge<br>Raine, Tim; Addenbrooke's Hospital; Department of Medicine, University of Cambridge

Keywords:	GASTROENTEROLOGY, Inflammatory bowel disease < GASTROENTEROLOGY, IMMUNOLOGY, Immunology < BASIC SCIENCES

SCHOLARONE™
Manuscripts

Trial Summary & Protocol for a phase II randomised placebo controlled double blinded trial of Interleukin 1 blockade in Acute Severe Colitis – the IASO Trial

Thomas MG (martin.thomas@addenbrookes.nhs.uk), Bayliss C, Bond S, Dowling F, Galea J, Jairath V, Lamb CA, Probert C, Timperley-Preece E, Watson A, Whitehead L, Williams JG, Parkes M, Kaser A, Raine T

Abstract

Introduction: Acute severe ulcerative colitis (ASUC) is a severe manifestation of ulcerative colitis (UC) that warrants hospitalisation. Despite significant advances in therapeutic options for UC and in the medical management of steroid-refractory ASUC, the initial treatment paradigm has not changed since 1955 and is based on the use of intravenous (IV) corticosteroids. This treatment is successful in approximately 50% of patients but failure of this and subsequent medical therapy still occurs, with colectomy rates of up to 40% reported. The **I**nterleukin 1 (IL-1) blockade in **A**cute **S**evere C**o**litis (IASO) trial aims to investigate whether antagonism of IL-1 signalling using anakinra in addition to IV corticosteroid treatment can improve outcomes in patients with ASUC.

Methods and Analysis: IASO is a multi-centre, two-arm (parallel group), randomised (1:1), placebo-controlled, double-blinded trial of anakinra in ASUC. Its primary outcome will be the incidence of medical or surgical rescue therapy (colectomy) within 10 days following the commencement of IV corticosteroid therapy. Secondary outcomes will include disease activity, time to clinical response, time to rescue therapy, colectomy incidence by day 98 post IV corticosteroids, and safety. The trial aims to recruit 214 patients across 20 sites in the UK.

Ethics and Dissemination: The Trial has received approval from the Cambridge Central Research Ethics Committee (Ref. 17/EE/0347), the Health Research Authority (Ref: 201505) and Clinical Trials Authorisation from the Medicines and Healthcare products Regulatory Agency. We plan to present trial findings at scientific conferences and publish in high-impact peer-reviewed journals.

Trial Registrations: ISRCTN: 43717130 (12/10/2017); EudraCT: 2017-001389-10 (30/03/2017)

Strengths and limitations of this study

- A multicentre, UK-wide trial of 214 participants at 20 sites
- The largest first-line treatment study ever conducted in this patient group
- Potential for a step-change and improved outcomes in acutely ill patients, including cost-savings for the NHS
- Pragmatic trial with wide eligibility criteria

For peer review only - http://bmjopen.bmj.com/site/about/guidelines.xhtml

Introduction

This article summarises the approved trial protocol in use at the time of publication (version 1.2 dated 13 October 2017). The full version of the protocol can be found in the supplementary appendix. The most current version of the protocol will be made available online at https://www.journalslibrary.nihr.ac.uk/programmes/eme/1420102/#/.

Background & Rationale

Ulcerative colitis (UC) is an inflammatory condition of the colon associated with diarrhoea, rectal bleeding and abdominal pain. UC affects around 120,000 people in the UK.[1] UC tends to follow a relapsing/remitting course with periods of comparative health interspersed with unpredictable acute 'flares'. These may be severe enough to mandate hospital admission. Annual UK hospitalisation rates with acute severe UC (ASUC) are around 3% (3,600 individuals) per annum, with 20-40% of patients requiring emergency surgical removal of the colon (colectomy) during the same hospital admission.[2,3] A recent UK audit of emergency surgery in colitis showed mortality rates of 4% at 90 days.[4] Additionally, surgery carries significant morbidity associated with the need for at least a temporary stoma, as well as long-term physical and psychological complications.

Treatment for ASUC involves high dose intravenous (IV) corticosteroids and is standardised worldwide.[5] After 3-5 days, patients who do not exhibit an improvement may be considered for medical 'rescue' therapy with further immunosuppression using either infliximab or ciclosporin. Those patients who subsequently fail to respond to medical treatment, or patients with fulminant colitis at presentation, will then proceed to surgery. This is usually performed on the same admission with a subtotal colectomy and creation of an end ileostomy.

The initial management of ASUC has changed little since the seminal trial of IV corticosteroids in 1955.[6] Despite significant advances in therapeutic options and drug development for ambulatory patients with UC, patients with ASUC are typically excluded from the eligibility criteria of standard clinical trials for patients with moderate-severe UC. Clinical trials that have compared infliximab or ciclosporin as medical rescue treatments for ASUC have required patients to fail to respond to treatment with up to 5 days of intravenous steroids.[7–9] In the absence of reliable early prognostic markers, it may be appealing to treat all patients presenting with ASUC with immunosuppressive treatments normally reserved for rescue therapy rather than corticosteroids alone. The barriers for this approach include the toxicity of such treatment as well as the financial burden incurred. There is still therefore an unmet need to improve the management of ASUC by doing something at the point of initial treatment, rather than waiting for treatment failure.

One of the key mediators of colonic inflammation in ASUC is interleukin-1 (IL-1) which plays a pivotal role in local activation of neutrophils and a number of downstream inflammatory mediators.[10] The IL-1 axis has been repeatedly identified as a therapeutic target in UC.[11–13] IL-1 antagonism occurs naturally via the ubiquitous IL-1 receptor antagonist (IL-1Ra). A recombinant form of this naturally-occurring anti-inflammatory, anakinra (Kineret®), has been used for the treatment of patients with rheumatoid arthritis and crypopyrin associated periodic syndromes (CAPS) with good effect. Anakinra has a very good safety profile in many disease states including RA, CAPS, severe sepsis and intracranial haemorrhage at standard subcutaneous (SC) dosing regimes and using high dose IV administration regimes.[14–16]

Our hypothesis is that antagonism of the IL-1 axis with anakinra is an effective way of abrogating the early inflammatory response seen in ASUC and leads to improved clinical outcomes.

There is evidence to suggest that co-treatment with corticosteroids may be beneficial, given that corticosteroids suppress the production of IL-1Ra to a greater extent than its active family members – IL-1α and IL-1β.[17] While recent research has seen a trend towards antagonism of IL-1 using monoclonal antibodies, anakinra may offer some advantages. Firstly, its relative

For peer review only - http://bmjopen.bmj.com/site/about/guidelines.xhtml

short half-life (4-6h) compared with antibodies allows early institution of further immunosuppression therapy if rescue therapy is required. Secondly, anakinra antagonises both IL-1α and IL-1β, while monoclonal antibodies are specific to either form. Finally, given that the patent for anakinra is now expired, the cost of the product is much less than that of the monoclonal antibody alternatives. Given this evidence, antagonism of IL-1 mediated inflammation early in the course of inflammation, in conjunction with standard corticosteroid treatment, is a promising therapeutic avenue in patients with ASUC.

Aims & Objectives

IASO aims to test whether anakinra can reduce the need for medical or surgical rescue therapy in patients with ASUC when given alongside IV corticosteroids. The trial also aims to assess the effects of anakinra on disease biology and bowel inflammation in addition to assessing drug safety, and longer-term health and patient-reported quality of life outcomes. Finally, the trial will seek to advance scientific understanding of ASUC through the collection and analysis of a large range of biological samples, including blood and tissue transcriptomic analysis, as well as microbial analysis. As part of this work, we will attempt to validate a transcriptional predictor of outcomes in UC that we have previously reported.[18]

Primary Objective

To compare the clinical effects of anakinra with placebo when given in addition to current standard care in patients with ASUC.

Secondary Objective

To compare the effects of anakinra with placebo when given in addition to current standard care to patients with ASUC in terms of:

- Safety
- Need for colectomy
- Patient-reported outcomes
- Endoscopic and histologic evidence of treatment effects
- Evidence of disruption of the IL-1 signalling pathway

For peer review only

For peer review only - http://bmjopen.bmj.com/site/about/guidelines.xhtml

Methods

Trial Design & Flowchart

IASO is a multi-centre, two arm (parallel group), randomised, placebo-controlled, double-blinded trial. The trial will also feature a sub-study specifically examining the endoscopic effects of treatment with anakinra. A summary of the trial design is shown in *Figure 1*.

[Figure 1]

Figure 1. IASO Trial Flowchart.

**Data collection for the primary endpoint and the colectomy-specific secondary endpoint is performed at days measured relative to the start of IMP treatment. However, the subsequent data analysis will assess these endpoints using time relative to the earlier start of IV corticosteroid treatment.*

Centres

IASO is a multi-centre study in UK NHS acute hospitals. We plan to include approximately 4 centres in the feasibility phase of the trial, increasing to 20 centres for the main phase.

All sites will recruit, treat and follow up participants equally, but a subset of sites (4) will also recruit participants into the optional endoscopic sub-study involving one additional flexible sigmoidoscopy with biopsy collection.

Trial duration

Participants' involvement in the trial will last approximately 6 months. This includes an initial treatment period of approximately 5–7 days, during which screening and an investigational medicinal product (IMP) treatment course of up to 5 days will be completed. Review of participant outcomes following trial participation may continue for up to 5 years using Hospital Episode Statistics (HES) data, without any additional burden to the participant.

Participant Selection

Inclusion Criteria

To be included in the trial the participant must:

- Be aged 16–80 years inclusive
- Have given written informed consent to participate
- Be hospitalised with clinically confirmed or suspected ASUC and a modified Truelove and Witts severity index (MTWSI)[7] score ≥11
- Have a requirement for treatment with IV corticosteroids in the judgement of the treating clinician, with the possibility to receive a first dose of IMP within 36 hours of commencement of IV corticosteroids

Exclusion Criteria

The presence of any of the following will preclude participant inclusion:

- Pregnant or breast-feeding women
- Oral corticosteroid dosing for a duration of 8 weeks or more immediately prior to commencement of IV corticosteroid dosing
- History of severe hepatic impairment (e.g. Child-Pugh = Grade C)
- Moderate or Severe renal impairment (estimated glomerular filtration rate [eGFR] <60ml/min/1.73m^2)

For peer review only - http://bmjopen.bmj.com/site/about/guidelines.xhtml

- Neutropenia (neutrophil count <1.5x10^9/l)
- Previous treatment with anakinra for any indication
- Documented hypersensitivity to the active substance or to any of the excipients or to E. coli derived proteins; latex allergy
- Evidence (from blood cultures etc) or clinical suspicion of systemic infection*
- Current or previous cytomegalovirus (CMV) infection requiring treatment with anti-viral agents
- Current treatment with anti-TNF-ɑ therapy or anti-TNF-ɑ discontinuation within previous 16 weeks
- A history of pulmonary TB infection
- Any absolute contraindication to IV corticosteroid
- History of malignancy (with the exception of non-melanoma skin cancer) or colonic dysplasia
- Rectal therapy in previous 14 days (sub-study exclusion only)
- Receipt of another IMP as part of a CTIMP within the previous 16 weeks

*Concurrent prescription of antibiotics to cover for the possibility of GI infection whilst awaiting stool culture results, or the possibility of bacterial translocation relating to severe colitis, is not an exclusion criterion where the physician suspects ulcerative colitis is the most likely diagnosis.

Outcome Measures

Primary Outcome

The primary outcome of the trial will be the incidence of medical or surgical rescue therapy (colectomy) within 10 days following the commencement of intravenous corticosteroid therapy.

For the purposes of the primary endpoint analysis, the time point of the start of rescue therapy will be as follows:

- Medical rescue therapy: Date and approximate time of first treatment administration
- Surgical rescue therapy: Date and approximate time of start of surgery

Secondary Outcomes

1. Incidence of Colectomy within 98 days following commencement of IV corticosteroid therapy.
2. Burden of disease activity, measured by daily MTWSI scores over Days 1-5 after initial IMP administration.
3. Time to clinical response (defined as 2nd consecutive day with MTWSI<10)
4. Time to medical or surgical rescue therapy, measured according to the time after the first dose of IV corticosteroids until the time that rescue therapy occurs (using definitions as set out in the primary endpoint). Data will be captured up to the same time point as the primary endpoint.
5. Incidence of SAEs, measured until Day 10 (+3) following commencement of IMP treatment.

Exploratory Outcomes

For peer review only - http://bmjopen.bmj.com/site/about/guidelines.xhtml

1. Patient-reported quality of life (EuroQol five dimensions; EQ-5D[19] and Crohn's and ulcerative colitis questionnaire-32; CUCQ-32[20]) at baseline and at 3 and 6 months following commencement of IMP treatment
2. Endoscopic response at Day 3 (± 1 day) (via standardised endoscopic scoring completed by a local and a central blinded assessor) following commencement of IMP treatment (endoscopic sub-study group only)

In addition to the above exploratory endpoints, the trial will also perform further exploratory analyses related to the identification of biomarkers and treatment response predictors, as well as characterisation of the stool microbial response to anakinra. Finally, patient readmission and colectomy data (e.g. via HES) may be examined to investigate the impact of treatment in the longer term.

Trial Treatment

For the purpose of this trial, the following are considered the IMP:

- Anakinra solution for injection
- Placebo to match anakinra solution for injection

IMP is supplied in commercial pre-filled syringe configurations (100mg/0.67ml), packed into trial-specific participant kits containing 11 pre-filled syringes. The syringes and kits have blinded labelling and are identified by unique pack (kit) numbers. IMP will be stored in accordance with the requirements of the SmPC for Anakinra.[21]

Participants will receive an initial loading dose of 100 mg IMP given intravenously (IV) within 36 hours of first IV corticosteroids. This will be followed by 100 mg SC IMP given twice daily, over a maximum of 5 days (maximum of 10 doses).

IV methylprednisolone or hydrocortisone, as per local guidelines for ASUC standard care, will be administered to participants as non-IMPs. Participants will continue to be treated with routine concomitant medications for other co-morbidities as appropriate within their current clinical condition, with modifications e.g. Oral to IV switches, or temporary cessation as necessary. Appropriate supportive care may also be prescribed, including fluids, nutritional supplements and other medications as necessary.

Participants will return to their normal standard of care as defined by their local physicians, following completion of trial treatment.

Treatment Termination & Trial Withdrawal

Participants may choose to withdraw from treatment or the full study at any stage without prejudice to standard clinical care.

Clinicians should cease treatment with the study drug according to their judgement or under any of the following criteria:

- Withdrawal of consent for treatment administration
- Development of renal impairment (eGFR <60 ml/min/1.73m^2) or severe hepatic impairment (Child-Pugh grade C)
- Development of neutropenia (neutrophil count <1.5x10^9/l)
- Detection of a significant GI pathogen in stool specimen
- Sufficient recovery to allow for cessation of IV steroids
- Hospital discharge due to recovery

For peer review only - http://bmjopen.bmj.com/site/about/guidelines.xhtml

- Decision to commence medical or surgical rescue therapy
- CMV reactivation necessitating treatment with anti-viral agents (in the judgement of treating clinicians)
- The development of a SAE necessitating termination of IMP treatment.

Where possible, trial assessments will continue following treatment termination.

In addition to the treatment termination criteria, the following criteria would result in participant withdrawal from the trial, with no further assessments being performed:

- Withdrawal of consent for further assessments and data collection
- Death

Randomisation & Stratification

All patients screened for the trial will be assigned a unique participant ID number. Suitable participants will subsequently be randomised (1:1) to drug:placebo using an online randomisation system accessible via password-protected access. Randomisation will be stratified for 2 variables:

- Previous vs current therapy with any of: immunomodulators (azathioprine, 6-mercaptopurine, 6-thioguanine, methotrexate, ciclosporin) biologics (anti-TNF-α monoclonal antibodies, anti-adhesion molecule antibodies, other anti-cytokine antibodies) or oral janus kinase inhibitors
- Current vs previous oral corticosteroid prescription(s) within 8 weeks prior to first dose of intravenous corticosteroids

Blinding

Trial participants, research teams and Site pharmacies will be blinded to the treatment group for the duration of the trial.

Procedures & Assessments

Participant Identification & Informed Consent

We will recruit inpatients with ASUC. Identification of potential participants will be undertaken by the clinical team with subsequent consenting and trial-specific procedures carried out by the research team.

A total of 40 individuals will be asked to join an optional scientific sub-study cohort. Participants can decline to participate in the sub-study but still participate in the main study.

Anonymised data on all participants who are approached will be collated in accordance with Consolidated Standards of Reporting Trials (CONSORT) guidelines.

Screening

The tests required for screening all form part of standard clinical care, therefore no additional tests will be performed other than a urine β-human chorionic gonadotropin (β-hCG) test for females where there is a possibility of pregnancy.

Baseline

The following activities will be performed and recorded at baseline:

- Medical history review

For peer review only - http://bmjopen.bmj.com/site/about/guidelines.xhtml

- Review of blood parameters (haemoglobin, total white blood cell, neutrophil, lymphocyte, monocyte, platelet, albumin and C-reactive protein) following a blood test performed as part of standard care
- 3 research blood samples (1 plasma tube, 1 nucleic acid tube, 1 genotyping tube) as described in the laboratory manual
- Concomitant medications review
- Demographics
- MTWSI assessment
- Stool sample collection
- EQ-5D and CUCQ-32 questionnaires

Randomisation & Post-Baseline Procedures

Following baseline data collection, participants will be randomised and allocated treatment as described above.

Prior to the first administration of IMP (Day 0), the following assessments will be performed:

- Review of adverse events
- Recording of any changes to concomitant medication since the last assessment
- Confirmation that IMP administration can proceed, according to the following criteria:
  - The time of administration of the first IMP dose falls no later than 36 hours following the time of first dose of IV corticosteroid.
  - IV corticosteroid therapy remains ongoing at the time of administration of the first IMP dose (i.e. that a decision to discontinue corticosteroids or transition to oral steroids has not been made after the time of participant consent to participate in the trial and before the time of the administration of the first IMP dose).
  - A firm decision to start rescue therapy or perform emergency colectomy has not already been taken

During the days subsequent to the first dose of trial medication, the following activities will be recorded for daily up to and including Day 5 following treatment initiation:

- MTWSI assessment / review of standard care clinical MTWSI assessment domains[#]
- Recording of any changes to concomitant medication prescription since the last assessment
- Review of blood parameters (haemoglobin, total white blood cell, neutrophil, lymphocyte, monocyte, platelet, albumin and C-reactive protein) following blood tests performed as part of standard care
- 2 research blood samples (1 plasma tube, 1 nucleic acid tube)*
- Review of adverse events
- Medical or surgical (emergency colectomy) rescue therapy assessment
- Stool sample collection on Day 5 (± 1 day) following commencement of IMP.

[#]Not recorded for patients who have undergone colectomy

*A minimum of 1 sample every 72 hours should be taken. Wherever possible every effort should be made to obtain the samples every day (up to 10 samples).

For peer review only - http://bmjopen.bmj.com/site/about/guidelines.xhtml

Sub-Study-Only Assessments

In addition to the daily assessments described above for Days 1-5, the scientific sub-study group will undergo an additional optional flexible sigmoidoscopy (without bowel preparation) on Day 3 (± 1 day) following commencement of IMP. This will be performed during their index inpatient admission.

Findings will be recorded according to a standardised endoscopic scoring system (Mayo)[22] by blinded assessors. Histology will be scored on up to a maximum of 6 biopsies taken from each of the rectum and sigmoid colon, according to validated scoring systems for colitis severity at the end of the trial.[23–25]

Where possible, endoscopic scores, text reports and photographic recordings of sigmoidoscopies will be sent to the central trial team for verification. In addition, where available, data collected as part of standard care (non-sub-study) endoscopies will be used to supplement trial-specific data. These will be scored by the blinded central assessor.

Follow-Up

Following the participants' inpatient stay, the following assessments will occur:

- Day 10 (+3) following commencement of IMP treatment – Rescue therapy assessment*
- Day 10 (+3) following commencement of IMP treatment – AE monitoring
- Day 98 (+14) following commencement of IMP treatment – Colectomy assessment†
- Approximately 3 and 6 months following commencement of IMP treatment – EQ-5D and CUCQ-32 questionnaires.

*In the event that a participant is discharged prior to Day 10 without having started rescue therapy, a member of the trial team will contact the participant on days Day 10 (+3 days) to confirm that the participant remains out of hospital and has not received rescue therapy elsewhere since discharge. If the participant is not contactable, local hospital records will be checked for admission.

†If not previously recorded, a member of the trial team will contact the participant on Day 98 (+14) to confirm that the participant remains out of hospital and has not undergone a colectomy since discharge. If the participant is not contactable, local hospital records will be checked for admission.

Participant status following discharge, including readmissions and subsequent surgery may be assessed using patient-specific HES data from the NHS Digital, Data Access Request Service. Any data collection would start at 1 year post-commencement of IMP treatment and examination of outcomes via HES data would potentially continue for up to 5 years, depending on study outcomes.

Safety Monitoring & Reporting

This section summarises the safety monitoring and reporting processes for the trial. Full details of the safety processes can be found in the comprehensive protocol included as part of the supplementary appendix.

Adverse events (AEs) will be recorded from the point of Informed Consent. Standard regulatory definitions for assessing AE seriousness will be used.[26]

As the IMP has a short half-life (<6h) no cumulative or late effects are anticipated. Therefore, recording of AEs and reporting of SAEs will be actively monitored up to Day 10 (+3 days). Following the end of the active monitoring period, investigators are still required to report and SARs or SUSARs of which they become aware.

For peer review only - http://bmjopen.bmj.com/site/about/guidelines.xhtml

For peer review only

Pregnancy reporting is only required for trial participants whilst they are receiving IMP and for up to 24 hours post their last dose.

Statistics & Data Management

Sample Size

We plan to include approximately 214 participants (107 per group) in the trial with 40 participants also included in the optional endoscopic sub-study. Recruitment of 214 patients to the main trial will give 85% power to detect a 20% absolute risk reduction (ARR) in the primary endpoint from a rate in the control group of 49% testing at the 5% significance level.

We have based our estimate of the primary endpoint rate on data from the recently completed NIHR-funded CONSTRUCT trial.[27] This trial recruited 270 patients with ASUC across 52 UK sites between 2010-2013. In CONSTRUCT, 49% of patients admitted with ASUC needed escalation to medical rescue therapy following treatment with corticosteroids (Prof J Williams, personal communication). Since there are no existing estimates of effect size for anakinra in ASUC, we chose to power IASO to detect a 20% ARR following protocol review by members of the British Society of Gastroenterology IBD Clinical Research Group, who advised that this was the minimum effect size needed that would make a substantial difference to the patient population, and which would be broadly in line with effect sizes seen in other drugs for moderate to severe colitis.[28–30]

The scientific sub-study based on testing cytokines will be performed on 40 participants. The choice of sample size is based on power studies in transcriptomic studies in UC[31] with adjustment for multiple testing, as well as pragmatic grounds of cost.

Patient Population

Populations to be analysed in the trial are as follows:

- Full Analysis Population: All randomised participants who receive at least one dose of IMP
- Randomised Population: All randomised participants, regardless of whether IMP was received
- Safety Population: All consenting participants

Any further populations may additionally be defined within the Statistical Analysis Plan.

Analyses

Primary Analysis

We will report summary statistics on the primary and secondary endpoints according to treatment group. The primary analysis will consist of an estimate, 95% confidence interval and p-value of the absolute risk difference of the incidence rates of the need for medical or surgical rescue therapy within 10 days following the first administration of IV corticosteroids between the two treatment arms, using logistic regression to adjust for important baseline covariates. The primary efficacy analysis will be based upon the Full Analysis Population.

The baseline characteristics will include:

- Prior diagnosis of IBD at the point of hospitalisation (‘first presentation’)
- Prior hospitalisation for ASUC
- Previous or current therapy with any of: immunomodulators (azathioprine, 6-mercaptopurine, 6-thioguanine, methotrexate, ciclosporin) biologics (anti-TNF-α monoclonal

For peer review only - http://bmjopen.bmj.com/site/about/guidelines.xhtml

antibodies, anti-adhesion molecule antibodies, other anti-cytokine antibodies) or oral janus kinase inhibitors

- Current or previous oral corticosteroid prescription(s) within 8 weeks prior to first dose of intravenous corticosteroids
- Demographics

Secondary endpoints will be compared in a similar regression model to estimate the treatment effect on an appropriate scale of comparison. This will include an assessment of the effects of treatment with anakinra on the secondary endpoints.

Secondary Analyses

The following secondary analyses will be performed in the trial:

- Analysis of the Randomised Population
- Complier Average Causal Effect analysis to assess the influence of the amount of treatment received, as distinct from treatment assigned

To test the hypothesis that the clinical effects of anakinra in ASUC may differ between groups of patients with differing levels of prior inflammatory burden, pre-specified subgroup analyses, in the form of estimating treatment-covariate interactions, will be performed on the following baseline characteristics:

- Prior diagnosis of IBD at the point of hospitalisation ('first presentation')
- Prior hospitalisation for ASUC
- Naïvety to any of: immunomodulators (azathioprine, 6-mercaptopurine, 6-thioguanine, methotrexate, ciclosporin) biologics (anti-TNF-α monoclonal antibodies, anti-adhesion molecule antibodies, other anti-cytokine antibodies) or oral janus kinase inhibitors at the point of hospitalisation.
- Receipt of oral corticosteroids within 8 weeks prior to first dose of intravenous corticosteroids
- Cases of suspected or confirmed ASUC without evidence of CMV reactivation requiring treatment with anti-viral agents and without evidence of a significant GI pathogen
- Duration between 1st dose of IV corticosteroids to 1st dose of IMP.

Interim Analyses

As part of planned interim analyses, an independent Data Monitoring Committee (iDMC) will examine unblinded data and provide a recommendation to a Trial Steering Committee (TSC) as to whether the trial should continue at two pre-specified points detailed below.

After the first 20 participants have been recruited, and all completed a minimum of 10 days follow up (to the time of the primary endpoint), trial feasibility will be assessed. The trial will be regarded as feasible only if all of the following measures are met:

1. ≥10% of eligible participants are recruited (based upon use of screening logs to determine the percentage of eligible participants that are randomised in each site)
2. Adherence to dosing as per protocol (≥80% of participants received appropriately prescribed doses within any given 24h window)
3. Rescue therapy started on or before the 7th day following initial IV corticosteroid administration in ≥80% cases where required during the index admission (as determined by review of timing of rescue therapy administration relative to corticosteroid administration by trial investigators)

For peer review only - http://bmjopen.bmj.com/site/about/guidelines.xhtml

4. Maintenance of blinding: there should be no evidence of unnecessary unblinding of participants or investigators.
5. No serious safety concerns identified, especially with regard to infections or SUSARs.

After the first 100 participants have completed Day 10 assessments, futility analysis will be performed to test the hypothesis that the reduction in absolute risk difference in the primary outcome is ≥10% using a 1-sided 2.5% significance test using logistic regression on the absolute risk reduction scale. If the hypothesis is rejected, i.e. the lower limit of a 95% 2-sided confidence interval for Treatment - Control is above -10%, then the iDMC will consider the recommendation for the study to stop early for futility. This is equivalent to spending 0.1% from the 15% total beta value set to control the type 2 error under the alternative hypothesis (49% and 29% rates). If the study continues past the interim, then the final analysis will not be adjusted for the futility analysis, and standard p-values and confidence intervals will be reported.

Data Management

All outcome data will be transferred onto a Case Report Form (CRF) which will be anonymised. Data captured on CRFs will subsequently be stored in a central database prior to analysis. Personal identifiable data kept for the purposes of questionnaire postage and HES-data capture will be destroyed at the end of the trial.

Missing Data

Due to short and intensive period of follow up for the primary endpoint, we do not anticipate significant missing data. Where we are unable to obtain missing data within 12 weeks of the 3- or 6-month time points, we will employ the standard approaches to management of missing quality of life data previously described in the CONSTRUCT trial.[27]

For participants who withdraw consent to treatment, permission to continue to acquire data for outcome analysis will be sought. For those who do not consent to ongoing monitoring, including those who wish to withdraw entirely from the study, existing data acquired to the point of withdrawal will be included in the final study analysis, with missing data handled according to standard missing data methods, including missing-at-random (MAR) methods with sensitivity testing for deviation from MAR assumptions.

Trial End

For regulatory notification purposes, the end of trial will be the earliest of:

- The receipt of the final, returned 6-month quality of life questionnaires
- 6 months (+12 weeks) after the last participant entered the study (date of questionnaire censoring).

However, participants may enter a period of long-term follow-up via HES data examination and the trial would remain open to the Ethics Committee until the last participant’s HES data capture has been completed.

Approvals

IASO has received ethical (17/EE/0347) and Health Research Authority approval. The trial is being carried out under a Clinical Trial Authorisation from the MHRA.

Sponsorship & Funding

The trial is sponsored by Cambridge University Hospitals NHS Foundation Trust and University of Cambridge.

For peer review only - http://bmjopen.bmj.com/site/about/guidelines.xhtml

This project is funded by the Efficacy and Mechanism Evaluation (EME) Programme, an MRC and NIHR partnership.* The views expressed in this publication are those of the author(s) and not necessarily those of the MRC, NHS, NIHR or the Department of Health.

*The EME Programme is funded by the MRC and NIHR, with contributions from the CSO in Scotland and NISCHR in Wales and the HSC R&D Division, Public Health Agency in Northern Ireland.

The trial drug is being provided by Swedish Orphan Biovitrum.

The Wellcome Trust Sanger Institute are providing financial support and academic collaboration for sample sequencing.

Patient and Public Involvement

Patient involvement was actively sought during the planning and preparation stage for this trial, and will form a key part of the trial as it progresses.

A 10-member patient steering group assisted with trial design in an early workshop, reviewed previous versions of the trial protocol and assisted with responses to the initial funding application review. The group provided valuable insight into the acceptability of different dosing regimens and into ways to support patients during the consent process. The patient information leaflet was reviewed by the group. To ensure that the views of the wider patient community were sought, an online questionnaire regarding willingness to participate in inpatient research studies in colitis was circulated to members of Crohn's and Colitis UK and responses used to inform trial design.

A patient representative will serve as a member of the TSC.

Dissemination Plan

This is an NIHR funded trial and the NIHR publishing guidelines will be followed. Authorship of final study outputs will be assigned in accordance with guidelines set out by the International Committee of Medical Journal Editors. Plain language summaries of key trial outputs will be prepared in conjunction with the patient steering group and published on the trial website. This website is listed in the patient information leaflet for the benefit of all trial participants.

Data Sharing

Anonymised datasets generated during and/or analysed during the trial will be stored in a publically available repository. Following completion of the trial and the analysis, it is anticipated that de-identified research data including sequencing data will be made available for other researchers. This will include use of database repositories such as Gene Expression Omnibus (GEO, https://www.ncbi.nlm.nih.gov/geo/), as well as sharing of participant-level datasets that underlie the results reported in published articles. Any clinical data will be shared with researchers who provide a methodologically sound proposal in order to achieve aims in the approved proposal.

Author Contributions

The full protocol was written by Martin Thomas, Tim Raine, Arthur Kaser, Carrie Bayliss and Francis Dowling. Sections related to Statistics and Data Analysis were written by Simon Bond. The Trial Treatment Section was written by Lynne Whitehead.

The study design was conceived by Tim Raine with intellectual input provided by Arthur Kaser, Miles Parkes, Alastair Watson, Simon Bond, Vipul Jairath, John Williams, Christopher Probert, James Galea, Elizabeth Timperley-Preece and Christopher Lamb.

For peer review only - http://bmjopen.bmj.com/site/about/guidelines.xhtml

Acknowledgements

The authors would like to thank the Cambridge Clinical Trials Unit for its support during the setup and conduct of the trial. The authors would also like to thank the patient steering group for its assistance with the trial design and for its ongoing involvement in the trial.

Christopher Lamb is a Clinical Lecturer supported by the NIHR.

Competing Interests

The authors have no competing interests to declare.

References

1 Molodecky NA, Soon IS, Rabi DM, *et al.* Increasing incidence and prevalence of the inflammatory bowel diseases with time, based on systematic review. *Gastroenterology* 2012;**142**:46–54 e42; quiz e30. doi:10.1053/j.gastro.2011.10.001

2 Lynch RW, Lowe D, Protheroe a, *et al.* Outcomes of rescue therapy in acute severe ulcerative colitis: data from the United Kingdom inflammatory bowel disease audit. *Aliment Pharmacol Ther* 2013;**38**:935–45. doi:10.1111/apt.12473

3 Dinesen LC, Walsh AJ, Protic MN, *et al.* The pattern and outcome of acute severe colitis. *J Crohn's Colitis* 2010;**4**:431–7. doi:10.1016/j.crohns.2010.02.001

4 NELA Project Team. Table 36. Third patient report of the National Emergency Laparotomy Audit. Published Online First: 2017.http://www.nela.org.uk/reports

5 Harbord M, Eliakim R, Bettenworth D, *et al.* Third European evidence-based consensus on diagnosis and management of ulcerative colitis. Part 2: Current management. *J Crohn's Colitis* 2017;**11**:769–84. doi:10.1093/ecco-jcc/jjx009

6 Truelove SC, Witts LJ. Cortisone in Ulcerative Colitis Final Report on a Therapeutic Trial. *Br Med J* 1955;**2**:1041–8. doi:10.1136/bmj.2.4947.1041

7 Lichtiger S, Present DH, Kornbluth A, *et al.* Cyclosporine in Severe Ulcerative Colitis Refractory to Steroid Therapy. *N Engl J Med* 1994;**330**:1841–5. doi:10.1056/NEJM199406303302601

8 Järnerot G, Hertervig E, Friis-Liby I, *et al.* Infliximab as rescue therapy in severe to moderately severe ulcerative colitis: A randomized, placebo-controlled study. *Gastroenterology* 2005;**128**:1805–11. doi:10.1053/j.gastro.2005.03.003

9 Williams JG, Alam MF, Alrubaiy L, *et al.* Infliximab versus ciclosporin for steroid-resistant acute severe ulcerative colitis (CONSTRUCT): a mixed methods, open-label, pragmatic randomised trial. *Lancet Gastroenterol Hepatol* 2016;**1**:15–24. doi:10.1016/S2468-1253(16)30003-6

10 Dinarello CA. Interleukin-1 in the pathogenesis and treatment of inflammatory diseases. *Blood* 2011;**117**:3720–32. doi:10.1182/blood-2010-07-273417

11 Planell N, Lozano JJ, Mora-Buch R, *et al.* Transcriptional analysis of the intestinal mucosa of patients with ulcerative colitis in remission reveals lasting epithelial cell alterations. *Gut* 2013;**62**:967–76. doi:10.1136/gutjnl-2012-303333

12 Dinarello CA, Simon A, van der Meer JWM. Treating inflammation by blocking interleukin-1 in a broad spectrum of diseases. *Nat Rev Drug Discov* 2012;**11**:633–52. doi:10.1038/nrd3800

13 Cominelli F, Pizarro TT. Interleukin-1 and interleukin-1 receptor antagonist in inflammatory bowel disease. *Aliment Pharmacol Ther* 1996;**10 Suppl 2**:49–53; discussion 54.http://www.ncbi.nlm.nih.gov/pubmed/8899101

For peer review only

For peer review only - http://bmjopen.bmj.com/site/about/guidelines.xhtml

14 Opal SM, Fisher CJ, Dhainaut JF, *et al.* Confirmatory interleukin-1 receptor antagonist trial in severe sepsis: a phase III, randomized, double-blind, placebo-controlled, multicenter trial. The Interleukin-1 Receptor Antagonist Sepsis Investigator Group. *Crit Care Med* 1997;**25**:1115–24. doi:10.1097/00003246-199707000-00010

15 Kullenberg T, Löfqvist M, Leinonen M, *et al.* Long-term safety profile of anakinra in patients with severe cryopyrin-associated periodic syndromes. *Rheumatol (United Kingdom)* 2016;**55**:1499–506. doi:10.1093/rheumatology/kew208

16 Fleischmann R, Tesser J, Schiff MH, *et al.* Safety of extended treatment with anakinra in patients with rheumatoid arthritis. *Ann Rheum Dis* 2006;**65**:1006–12. doi:10.1136/ard.2005.048371

17 Langereis JD, Oudijk EJD, Schweizer RC, *et al.* Steroids induce a disequilibrium of secreted interleukin-1 receptor antagonist and interleukin-1Beta synthesis by human neutrophils. *Eur Respir J* 2011;**37**:406–15. doi:10.1183/09031936.00170409

18 Lee JC, Lyons P a, McKinney EF, *et al.* Gene expression profiling of CD8+ T cells predicts prognosis in patients with Crohn disease and ulcerative colitis. *J Clin Invest* 2011;**121**:4170–9. doi:10.1172/JCI59255

19 EuroQol - a new facility for the measurement of health-related quality of life. *Health Policy (New York)* 1990;**16**:199–208. doi:10.1016/0168-8510(90)90421-9

20 Alrubaiy L, WY C, Dodds P, *et al.* Development of a short questionnaire to assess the quality of life in Crohn's disease and ulcerative colitis. *J Crohns Colitis* 2015;**9**:66–76. doi:10.1093/ecco-jcc/jju005

21 European Medicines Agency. Kineret (Anakinra) Summary of Product Characteristics. Available at: https://www.medicines.org.uk/emc/product/559.

22 Schroeder KW, Tremaine WJ, Ilstrup DM. Coated Oral 5-Aminosalicylic Acid Therapy for Mildly to Moderately Active Ulcerative Colitis. *N Engl J Med* 1987;**317**:1625–9. doi:10.1056/NEJM198712243172603

23 Geboes K, Riddell R, Öst A, *et al.* A reproducible grading scale for histological assessment of inflammation in ulcerative colitis. *Gut* 2000;**47**:404–9. doi:10.1136/gut.47.3.404

24 Marchal-Bressenot A, Salleron J, Boulagnon-Rombi C, *et al.* Development and validation of the Nancy histological index for UC. *Gut* 2017;**66**:43–9. doi:10.1136/gutjnl-2015-310187

25 Mosli MH, Feagan BG, Zou G, *et al.* Development and validation of a histological index for UC. *Gut* 2017;**66**:50–8. doi:10.1136/gutjnl-2015-310393

26 European Medicines Agency. NOTE FOR GUIDANCE ON CLINICAL SAFETY DATA MANAGEMENT: DEFINITIONS AND STANDARDS FOR EXPEDITED REPORTING (CPMP/ICH/377/95). Available at: http://www.ema.europa.eu/docs/en_GB/document_library/Scientific_guideline/2009/09/WC500002749.pdf. 1995.

27 Williams JG, Alam MF, Alrubaiy L, *et al.* Comparison Of iNfliximab and ciclosporin in STeroid Resistant Ulcerative Colitis: pragmatic randomised Trial and economic evaluation (CONSTRUCT). *Health Technol Assess* 2016;**20**:1–320. doi:10.3310/hta20440

28 Rutgeerts P, Sandborn WJ, Feagan BG, *et al.* Infliximab for Induction and Maintenance Therapy for Ulcerative Colitis. *N Engl J Med* 2005;**353**:2462–76. doi:10.1056/NEJMoa050516

29 Feagan BG, Rutgeerts P, Sands BE, *et al.* Vedolizumab as Induction and Maintenance Therapy for Ulcerative Colitis. *N Engl J Med* 2013;**369**:699–710. doi:10.1056/NEJMoa1215734

30 Sandborn WJ, Su C, Sands BE, *et al.* Tofacitinib as Induction and Maintenance Therapy for Ulcerative Colitis. *N Engl J Med* 2017;**376**:1723–36. doi:10.1056/NEJMoa1606910

For peer review only - http://bmjopen.bmj.com/site/about/guidelines.xhtml
For peer review only

31 Boland BS, Boyle DL, Sandborn WJ, *et al.* Validated gene expression biomarker analysis for biopsy-based clinical trials in ulcerative colitis. *Aliment Pharmacol Ther* 2014;**40**:477–85. doi:10.1111/apt.12862

For peer review only

For peer review only - http://bmjopen.bmj.com/site/about/guidelines.xhtml

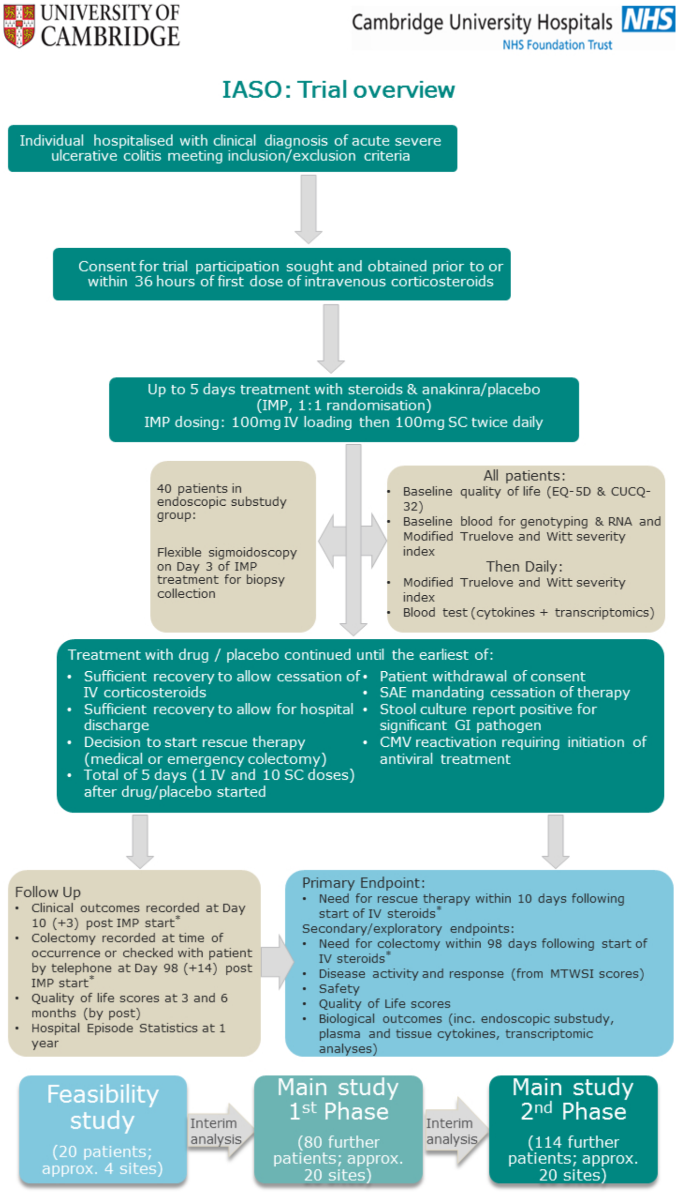

Figure 1. IASO Trial Flowchart.
*Data collection for the primary endpoint and the colectomy-specific secondary endpoint is performed at days measured relative to the start of IMP treatment. However, the subsequent data analysis will assess these endpoints using time relative to the earlier start of IV corticosteroid treatment.

Clinical Trial Protocol

Trial Title:	A phase II randomised placebo controlled double blinded trial of **I**nterleukin 1 blockade in **A**cute **S**evere C**o**litis
Protocol Number:	CCTU0165
EudraCT Number:	2017-001389-10
ISRCTN Number:	43717130
Investigational Product:	Anakinra (Kineret®)
Protocol Version:	1.2

Chief Investigator:	Professor Arthur Kaser
CI Address:	Department of Medicine, Box 157, Addenbrooke's Hospital, Cambridge CB2 0QQ, UK
Telephone:	01223 331130
Trial Sponsor:	Cambridge University Hospitals NHS Foundation Trust and the University of Cambridge
Trial Funder:	National Institute for Health Research and Medical Research Council
SAE Reporting:	Cambridge Clinical Trials Unit (CCTU)<br>Clinical Trial Coordinator: Dr Martin Thomas<br>Telephone: 01223 254 920<br>Fax: 01223 254247<br>Email: add-tr.iaso@nhs.net

For peer review only - http://bmjopen.bmj.com/site/about/guidelines.xhtml

1 Protocol Signatures:

I give my approval for the attached protocol entitled "A phase II randomised placebo controlled double blinded trial of **I**nterleukin 1 blockade in **A**cute **S**evere C**o**litis" version 1.2, dated 13 October 2017.

Chief Investigator

Name: Professor Arthur Kaser

Signature: ______________________________

Date: ______________________

Trial Statistician

Name: Dr Simon Bond

Signature: ______________________________

Date: ______________________

Site Signatures

I have read the attached protocol entitled "A phase II randomised placebo controlled double blinded trial of **I**nterleukin 1 blockade in **A**cute **S**evere C**o**litis" version 1.2, dated 13 October and agree to abide by all provisions set forth therein.

I agree to comply with the conditions and principles of Good Clinical Practice as outlined in the European Clinical Trials Directives 2001/20/EC and 2005/28/EC, the Medicines for Human Use (Clinical Trials) Regulations 2004 (SI 2004/1031) and any subsequent amendments of the clinical trial regulations, the Sponsor's SOPs, and other regulatory requirements as amended.

Name: ______________________________

Signature: ______________________________

Date: ______________________

2 Protocol and amendment history

Amendment No.	Protocol version no.	Date	Details of changes made
1	1.1	18 September 2017	Modified Section 11.4 and changed units/value of eGFR as part of the response to the MHRA review.
2	1.2	13 October 2017	ISRCTN number added, emc website link removed from Section 11.1.1.8

For peer review only - http://bmjopen.bmj.com/site/about/guidelines.xhtml

For peer review only

For peer review only

For peer review only - http://bmjopen.bmj.com/site/about/guidelines.xhtml

3 Trial Management Committee(s) and Protocol Contributors

Chief Investigator	Prof Arthur Kaser, Department of Medicine, Box 157, Addenbrooke's Hospital, Cambridge CB2 0QQ, UK<br>01223 331130<br>ak729@cam.ac.uk
Lead Investigator	Dr Tim Raine, Department of Medicine, Box 157, Addenbrooke's Hospital, Cambridge CB2 0QQ, UK<br>01223 331130<br>tr223@cam.ac.uk
Trial Coordinator	Dr Martin Thomas, Cambridge Clinical Trials Unit, Addenbrooke's Hospital, Cambridge CB2 0QQ, UK<br>01223 254920<br>martin.thomas@addenbrookes.nhs.uk
Statistician	Dr Simon Bond Cambridge, Cambridge Clinical Trials Unit,<br>Addenbrooke's Hospital, Cambridge CB2 0QQ, UK<br>01223 596475<br>simon.bond@addenbrookes.nhs.uk
Trials Pharmacist	Dr Lynne Whitehead, Central Pharmacy, Addenbrooke's Hospital, Cambridge CB2 0QQ, UK<br>01223 216057<br>lynne.whitehead@addenbrookes.nhs.uk
Protocol Contributors	Dr Miles Parkes, Department of Medicine, Box 157, Addenbrooke's Hospital, Cambridge CB2 0QQ, UK<br><br>Prof Alastair Watson, Department of Medicine, University of East Anglia, Norwich NR4 7TJ, UK<br><br>Dr Christopher Lamb, Institute of Cellular Medicine, Newcastle University, Newcastle upon Tyne NE2 4HH, UK<br><br>Prof John Williams, Swansea University College of Medicine,<br>, Swansea SA2 8PP, UK<br><br>Prof Chris Probert, Department of Gastroenterology, University of Liverpool, Liverpool L69 3GE, UK<br><br>Dr Vipul Jairath, Nuffield Department of Medicine, University of Oxford, Headington, Oxford OX3 7BN, UK<br><br>Mr James Galea, University of Manchester, Salford Royal NHS Foundation Trust, Salford M6 8HD, UK
DMC chair	Dr Peter Irving, St Thomas' Hospital, Westminster Bridge Road, London, SE1 7EH, UK<br>+44 (0)207 188 2499<br>peter.irving@kcl.ac.uk
TSC chair	Dr Oliver Brain, John Radcliffe Hospital, Headley Way, Headington, Oxford OX3 9DU, UK<br>+44 (0)1865 228755<br>Oliver.Brain@ouh.nhs.uk

For peer review only

For peer review only - http://bmjopen.bmj.com/site/about/guidelines.xhtml

TSC Patient representative	Harriet Boswell
Central Blinded Edoscopy Assessor	Dr Tim Raine, Department of Medicine, Box 157, Addenbrooke's Hospital, Cambridge CB2 0QQ, UK<br>01223 331130<br>tr223@cam.ac.uk

For peer review only - http://bmjopen.bmj.com/site/about/guidelines.xhtml

Table of Contents

1 Protocol Signatures: ... 2
2 Protocol and amendment history ... 2
3 Trial Management Committee(s) and Protocol Contributors ... 4
Table of Contents ... 6
4 Abbreviations ... 8
5 Trial Synopsis ... 9
6 Trial Flow Chart ... 14
7 Introduction ... 15
7.1 Background ... 15
7.2 Clinical Data ... 15
8 Rationale for Trial ... 17
9 Trial Design ... 18
9.1 Statement of Design ... 18
9.2 Number of Centres ... 19
9.3 Number of Participants ... 19
9.4 Participants Trial Duration ... 19
9.5 Trial Objectives ... 19
9.6 Trial Outcome Measures ... 20
10 Selection and withdrawal of participants ... 21
10.1 Inclusion Criteria ... 21
10.2 Exclusion Criteria ... 21
10.3 Patient Trial ID and Treatment Assignment ... 22
10.4 Method of Blinding ... 22
10.5 Participant Withdrawal Criteria ... 22
11 Trial Treatments ... 23
11.1 Treatment Summary ... 23
11.2 Non Investigational Medicinal Products ... 26
11.3 Concomitant Therapy ... 26
11.4 Emergency unblinding ... 26
11.5 Accountability and dispensing ... 26
12 Procedures and assessments ... 27
12.1 Participant identification ... 27
12.2 Consent ... 27
12.3 Screening evaluation ... 28
12.4 Baseline Assessments ... 28
12.5 Trial assessments ... 28
12.6 Schedule of Assessments ... 32
12.7 Long-Term Follow-up Assessments ... 33
12.8 End of Trial Participation ... 33
12.9 Trial restrictions ... 33
13 Assessment of Safety ... 33
13.1 Definitions ... 33
13.2 Expected Adverse Reactions/Serious Adverse Reactions (AR /SARs) ... 34
13.3 Expected Adverse Events/Serious Adverse Events (AE/SAE) ... 34
13.4 Evaluation of adverse events ... 35
13.5 Reporting serious adverse events ... 36
13.6 Reporting of Suspected Unexpected Serious Adverse Reactions (SUSARs) ... 36
13.7 Pregnancy Reporting ... 38
14 Toxicity/Reaction Management ... 38
15 Evaluation of Results (Definitions and response/evaluation of outcome measures) ... 38

For peer review only - http://bmjopen.bmj.com/site/about/guidelines.xhtml

15.1 Response criteria 38
16 Storage and Analysis of Samples 38
17 Statistics 38
17.1 Statistical methods 38
17.2 Interim analyses and criteria for the premature termination of the trial 40
17.3 Number of Participants to be enrolled 41
17.4 Procedure to account for missing or spurious data 42
17.5 Definition of the end of the trial 42
18 Data handling and record keeping 42
18.1 CRF 42
18.2 Source Data 43
18.3 Data Protection & Participant Confidentiality 43
19 Data Monitoring Committee/Trial Steering Committee 43
20 Ethical & Regulatory considerations 44
20.1 Ethical committee review 44
20.2 Regulatory Compliance 44
20.3 Protocol Amendments 44
20.4 Peer Review 44
20.5 Public and Patient Involvement 45
20.6 Declaration of Helsinki and Good Clinical Practice 45
20.7 GCP Training 45
21 Sponsorship, Financial and Insurance 45
22 Monitoring, Audit & Inspection 46
23 Protocol Compliance and Breaches of GCP 46
24 Publications policy 46
25 Appendices 47
25.1 Appendix 1 – Authorisation of Participating Sites 47
25.2 Appendix 2 – Safety Reporting Flow Chart 49
25.3 Appendix 3 – Amendment History 50
25.4 Appendix 4 – EQ-5D and CUCQ-32 Questionnaires 50
25.5 Appendix 4 – Treatment decision tree for IASO trial 56
26 REFERENCES 57

For peer review only - http://bmjopen.bmj.com/site/about/guidelines.xhtml

4 Abbreviations

AE	Adverse Event
APR	Annual Progress Report
AR	Adverse Reaction
ARR	Absolute Risk Reduction
ASUC	Acute Severe Ulcerative Colitis
BD	Twice Daily
β-hCG	β-Human Chorionic Gonadotropin
BSG	British Society of Gastroenterology
BSG IBD CRG	BSG IBD Clinical Research Group
CAPS	Cryopyrin-Associated Periodic Syndromes
CCTU	Cambridge Clinical Trials Unit
CCUK	Crohn's & Colitis UK
CI	Chief Investigator
CMV	Cytomegalovirus
CONSORT	Consolidated Standards of Reporting Trials
CRF	Case Report Form
CRP	C-Reactive Protein
CTA	Clinical Trial Authorisation
CTIMP	Clinical Trial of Investigational Medicinal Product
CUCQ-32	Crohn's and ulcerative colitis questionnaire-32
CYP450	Cytochrome p450
DIBD	Developmental International Birth Date
DSUR	Development Safety Update Report
eGFR	Estimated Glomerular Filtration Rate
EME	Efficacy and Mechanism Evaluation
EQ-5D	EuroQol five dimensions
EudraCT	European Clinical Trials Database
GCP	Good Clinical Practice
GI	Gastrointestinal
GMP	Good Manufacturing Practice
HES	Hospital Episode Statistics
HRA	Health Research Authority
IB	Investigator Brochure
IBD	Inflammatory Bowel Disease
IL-1	Interleukin-1 (including A and B variants)
IL-1R	Interleukin-1 Receptor
IL-1RA	Interleukin-1 Receptor Antagonist
IL-6	Interleukin-6
IL-10	Interleukin-10
iDMC	Independent Data Monitoring Committee
IMP	Investigational Medicinal Product
ISF	Investigator Site File
ISRCTN	International Standard Randomised Controlled Trials Number
IV	Intravenous
MAR	Missing-at-random
MHRA	Medicines and Healthcare products Regulatory Agency
MRC	Medical Research Council
mRNA	Messenger Ribonucleic Acid
MTWSI	Modified Truelove and Witts Severity Index

For peer review only - http://bmjopen.bmj.com/site/about/guidelines.xhtml

For peer review only

NHS R&D	National Health Service Research & Development
NIHR	National Institute for Health Research
NIMP	Non-Investigational Medicinal Product
PI	Principal Investigator
PK	Pharmacokinetic
RA	Rheumatoid Arthiritis
RSI	Reference Safety Information
R&D	Research & Development
RCT	Randomised Control Trial
REC	Research Ethics Committee
SAE	Serious Adverse Event
SAR	Serious Adverse Reaction
SC	Subcutaneous
SOP	Standard Operating Procedure
SmPC	Summary of Product Characteristics
SUSAR	Suspected Unexpected Serious Adverse Reaction
TB	Tuberculosis
TMG	Trial Management Group
TSC	Trial Steering Committee
TMF	Trial Master File
TNF-α	Tumour Necrosis Factor α
UC	Ulcerative Colitis
UK	United Kingdom

5 Trial Synopsis

Title of clinical trial	A phase II randomised placebo controlled double blinded trial of Interleukin 1 blockade in Acute Severe Colitis
Sponsor name	Cambridge University Hospitals NHS Foundation Trust and the University of Cambridge
EudraCT number	2017-001389-10
Medical condition or disease under investigation	Acute severe ulcerative colitis
Purpose of clinical trial	To evaluate whether anakinra is an effective treatment for patients with acute severe ulcerative colitis
Primary objective	To compare the clinical effects of anakinra with placebo when given in addition to current standard care in patients with acute severe colitis.
Secondary objective (s)	To compare the effects of anakinra with placebo when given in addition to current standard care to patients with acute severe colitis in terms of:<br>• Safety<br>• Need for colectomy<br>• Patient reported outcomes<br>• Endoscopic and histologic evidence of treatment effects<br>• Evidence of disruption of the IL-1 signalling pathway

For peer review only - http://bmjopen.bmj.com/site/about/guidelines.xhtml

Trial Design	Phase II, Randomised, placebo controlled, double blind
Primary Outcome Measure	Need for medical or surgical rescue therapy within 10 days following commencement of intravenous corticosteroid therapy.
Secondary Outcome Measures	• Need for colectomy within 98 days following commencement of intravenous corticosteroid therapy<br>• Disease activity<br>• Time to clinical response<br>• Time to rescue therapy (medical or surgical)<br>• Incidence of serious adverse events<br><br>Exploratory Outcome Measures<br><br>• Patient-reported quality of life (EQ-5D[25] and CUCQ-32[26]) at baseline and at 3 and 6 months following commencement of IMP treatment<br>• Centrally- and locally-scored endoscopic response at Day 3 (± 1 day) following commencement of IMP treatment (endoscopic sub-study group only)
Planned Sample Size	214 patients (subject to satisfactory progress through feasibility and interim analyses)<br>40 patients will participate in the endoscopic sub-study
Summary of eligibility criteria	Inclusion Criteria:<br>• Patients aged 16–80 inclusive<br>• Hospitalised patients with clinically confirmed or suspected ASUC and a MTWSI score ≥11<br>• Requirement for treatment with IV corticosteroids in the judgement of the treating clinician, with the possibility to receive a first dose of IMP within 36 hours of commencement of IV corticosteroids<br>Exclusion Criteria<br>• Pregnant or breast-feeding women<br>• Oral corticosteroid dosing for a duration of 8 weeks or more immediately prior to commencement of IV corticosteroid dosing<br>• History of severe hepatic impairment (e.g. Child-Pugh = Grade C)<br>• Moderate or severe renal impairment (eGFR <60ml/min/1.73m^2)<br>• Neutropenia (neutrophil count <1.5x10^9/l)<br>• Previous treatment with anakinra for any indication<br>• Documented hypersensitivity to the active substance or to any of the excipients or to

For peer review only - http://bmjopen.bmj.com/site/about/guidelines.xhtml

	E. coli derived proteins; latex allergy<br>• Evidence (from blood cultures etc) or clinical suspicion of systemic infection*<br>• Current or previous cytomegalovirus (CMV) infection requiring treatment with anti-viral agents<br>• Current treatment with anti-TNF-α therapy or anti-TNF-α discontinuation within previous 16 weeks<br>• A history of pulmonary TB infection<br>• Any absolute contraindication to IV corticosteroid<br>• History of malignancy (with the exception of non-melanoma skin cancer) or colonic dysplasia<br>• Rectal therapy in previous 14 days (sub-study exclusion only)<br>• Receipt of another IMP as part of a CTIMP within the previous 16 weeks<br>*concurrent prescription of antibiotics to cover the possibility of GI infection whilst awaiting stool culture results, or the possibility of bacterial translocation relating to severe colitis, is not an exclusion criterion where the physician suspects ulcerative colitis is the most likely diagnosis.
Investigational medicinal product and dosage	100 mg Anakinra and matched placebo<br><br>Single 100mg intravenous loading dose followed by 100mg subcutaneous dose twice daily (up to 10 doses)
NIMPs	IV methylprednisolone or hydrocortisone, as per local guidelines for ASUC standard care.
Route(s) of administration	Intravenous and subcutaneous
Maximum duration of treatment of a participant	5 days (1 IV dose, 10 SC doses)
Procedures: Screening & enrolment	Identification of potential trial participants will be performed by the clinical care team. Consenting and screening of potential participants will be performed at each participating site by trial investigators, or by a suitably qualified and delegated member of the trial team.<br><br>A total of 40 participants recruited at 4 centres will be asked to join an optional scientific sub-study cohort. Participants can decline to participate in the sub-study but still participate in the main study.<br><br>Screening will be performed based upon the eligibility criteria and will involve:<br>• Review of the inclusion/exclusion criteria

For peer review only - http://bmjopen.bmj.com/site/about/guidelines.xhtml
For peer review only

	• Urine β-hCG, if necessary
Procedures: Baseline	The following activities will be performed and recorded at baseline:<br>• Medical history review<br>• Review of routine blood parameters<br>• 3 research blood samples (1 plasma tube, 1 nucleic acid tube, 1 genotyping tube)<br>• Concomitant medications review<br>• Demographics<br>• MTWSI Assessment / review of standard care MTWSI assessments<br>• Stool sample collection<br>• EQ-5D and CUCQ-32 questionnaires
Procedures:<br>Treatment period	Administration of IMP as indicated in the dosing schedule.<br><br>Daily activities following initial dosing:<br>• MTWSI Assessment / review of standard care MTWSI assessments<br>• Recording of any changes to concomitant medication prescription since the last assessment<br>• Review of blood parameters following blood tests performed as part of standard care<br>• 2 research blood samples (1 plasma tube, 1 nucleic acid tube)<br>• Review of adverse events<br>• Medical or surgical (emergency colectomy) rescue therapy assessment<br><br>In addition to the daily data collection described above for Days 1-5, a stool sample will be collected on Day 5 (± 1 day).<br><br>As part of the sub-study participants will be undergo an additional flexible sigmoidoscopy (without bowel preparation) and biopsy sampling on Day 3 (± 1 day) post commencement of IMP treatment.
Procedures:<br>Follow Up period	Following the participants' inpatient stay, unless specified otherwise (See Section 12.5), assessments will occur at the following timepoints:<br>• Day 10 (+3) – Rescue therapy assessment & AE review<br>• Day 98 (+ 14) – colectomy assessment<br>• Approximately 3 and 6 months following commencement of IMP treatment – participants will be contacted by post and asked to complete the EQ-5D and CUCQ-32 questionnaires.

For peer review only - http://bmjopen.bmj.com/site/about/guidelines.xhtml

Procedures: End of trial	Participant status during following discharge, including readmissions and subsequent surgery may be assessed using patient-specific HES and equivalent data from NHS Digital. Data collection would start at 1 year post-commencement of IMP treatment and possible examination of outcomes via HES data would potentially continue for up to 5 years, depending on study outcomes.
Procedures for safety monitoring during trial	The DMC and TSC will periodically review safety data during the trial.
Criteria for withdrawal of participants	Treatment termination criteria<br><br>Participants may withdraw from treatment or the full study at any time without prejudice to their clinical care. In addition, responsible clinicians should cease treatment with the study drug according to their judgement or under any of the following criteria:<br>• Development of renal impairment (eGFR rate <60 ml/min/1.73m^2) or severe hepatic impairment (Child Pugh grade C)<br>• Development of neutropenia (neutrophil count <1.5x10^9/l)<br>• Detection of a significant GI pathogen in stool specimen<br>• Sufficient recovery to allow for cessation of IV steroids<br>• Hospital discharge due to recovery<br>• Decision to commence medical or surgical rescue therapy<br>• CMV reactivation necessitating treatment with anti-viral agents (in the judgement of treating clinicians)<br>• The development of a SAE necessitating termination of IMP treatment.<br><br>Trial withdrawal criteria<br>In addition to the treatment termination criteria, the following criteria would result in participant withdrawal from the trial, with no further assessments being performed:<br>• Withdrawal of consent to collect data<br>• Death

For peer review only

For peer review only - http://bmjopen.bmj.com/site/about/guidelines.xhtml

6 Trial Flow Chart

UNIVERSITY OF CAMBRIDGE

Cambridge University Hospitals NHS
NHS Foundation Trust

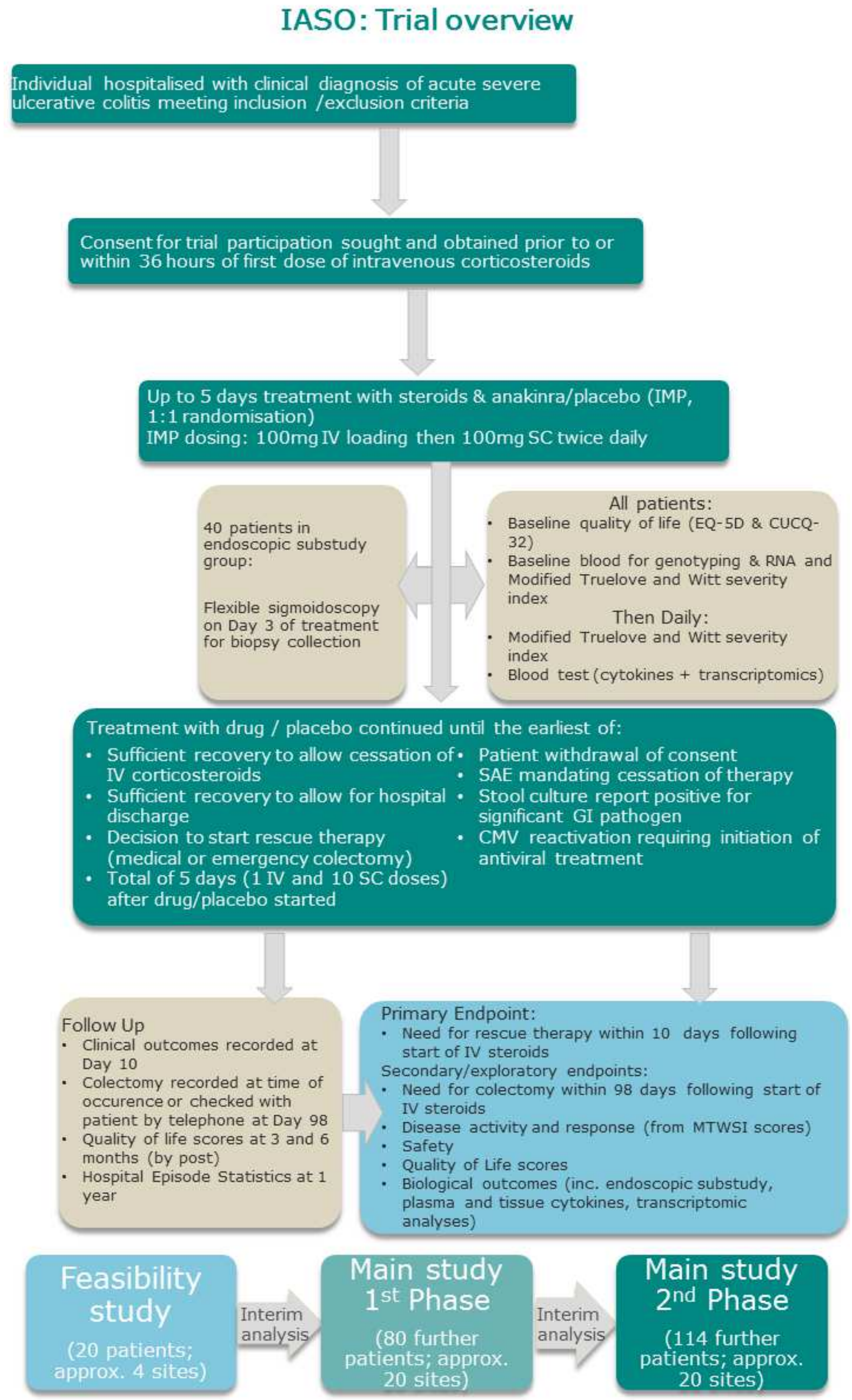

For peer review only - http://bmjopen.bmj.com/site/about/guidelines.xhtml

7 Introduction

7.1 Background

Acute severe ulcerative colitis carries a burden of morbidity and mortality with limited treatment options

Ulcerative colitis (UC) is an inflammatory condition of the colon associated with diarrhoea, rectal bleeding and abdominal pain. UC affects around 120,000 people in the UK. UC tends to follow a relapsing/remitting course with periods of comparative health interspersed with unpredictable acute 'flares'. These may be severe enough to mandate hospital admission: Annual UK hospitalisation rates with acute severe UC (ASUC) are around 3% (3600 individuals) per annum, with 20-40% of patients requiring emergency surgical removal of the colon (colectomy) during the same hospital admission. A recent UK audit of emergency surgery in colitis showed significant mortality rates[1]. Additionally, surgery carries significant morbidity associated with the need for at least a temporary external drain for the faecal material through the skin (stoma), as well as long-term physical and psychological complications.

The care of patients with ASUC follows standardised pathways throughout Europe and much of the rest of the world[2]. Patients meeting diagnostic criteria for ASUC are started on high dose intravenous (IV) corticosteroids. After 3-5 days, patients who are not meeting criteria for improvement may be considered for 'rescue' therapy with further immunosuppression using either infliximab or ciclosporin. Infliximab is costly, and although ciclosporin is a cheaper drug, it requires monitoring and is associated with complications that significantly increase the overall cost. Those patients who fail to respond to medical treatment will then proceed to a colectomy usually performed within 30 days of admission, with removal of most of the colon, and creation of a stoma.

Colonic inflammation in ASUC is characterised by neutrophilic infiltration and high levels of interleukin-1

Colonic inflammation in ASUC is marked by infiltration of neutrophils. These express high levels of a range of inflammatory cytokines, including tumour necrosis factor-α (TNF-α, the molecular target of infliximab) and interleukin-1 (IL-1). IL-1 plays a pivotal role in driving neutrophil activation and a number of downstream inflammatory mediators[3]. High levels of IL-1 are found in inflamed colonic tissue from patients with ASUC[4], and the concentration of IL-1 in the inflamed bowel correlates with endoscopic measurements of severity[5, 6]. The IL-1 axis has been repeatedly identified as a therapeutic target in UC, but this hypothesis has yet to lead to a published clinical trial[4, 7, 8]. The receptor for IL-1 is blocked by an antagonist known as anakinra, a recombinant protein similar to the endogenous protein known as IL-1 receptor antagonist (IL-1RA).

7.2 Clinical Data

Anakinra is a recombinant protein that blocks IL-1 receptor signalling

Anakinra is a low cost drug that has been studied in a number of different indications, but there are no published trials of its use in UC. During the 1990s a clinical trials programme in patients with sepsis culminated in phase III studies that found no evidence of benefit, but importantly, no evidence of an adverse safety signal (and importantly, no evidence of worsening of infectious complications) despite the drug being given at high doses to critically ill patients with systemic infections[9]. The drug is currently licensed for use in patients with rheumatoid arthritis following successful clinical studies that again showed a favourable safety profile[10]. More recent studies have used anakinra in a wide range of indications and doses. Anakinra has recently been used successfully to treat 2 patients with inflammation of the colon related to

For peer review only - http://bmjopen.bmj.com/site/about/guidelines.xhtml

chronic granulomatous disease[11], as well as in a number of animal models of colitis[11, 12].

Anakinra has a short half-life when given IV, but can achieve stable plasma concentrations with subcutaneous (SC) dosing[13]. Using a pharmacokinetic (PK) model informed by tissue microdialysis data and serum measurements in patients receiving anakinra in the context of a clinical trial, we have modelled a dosing regimen for anakinra based upon rapid IV loading (100 mg) then 100 mg twice daily SC doses (Figure 1).

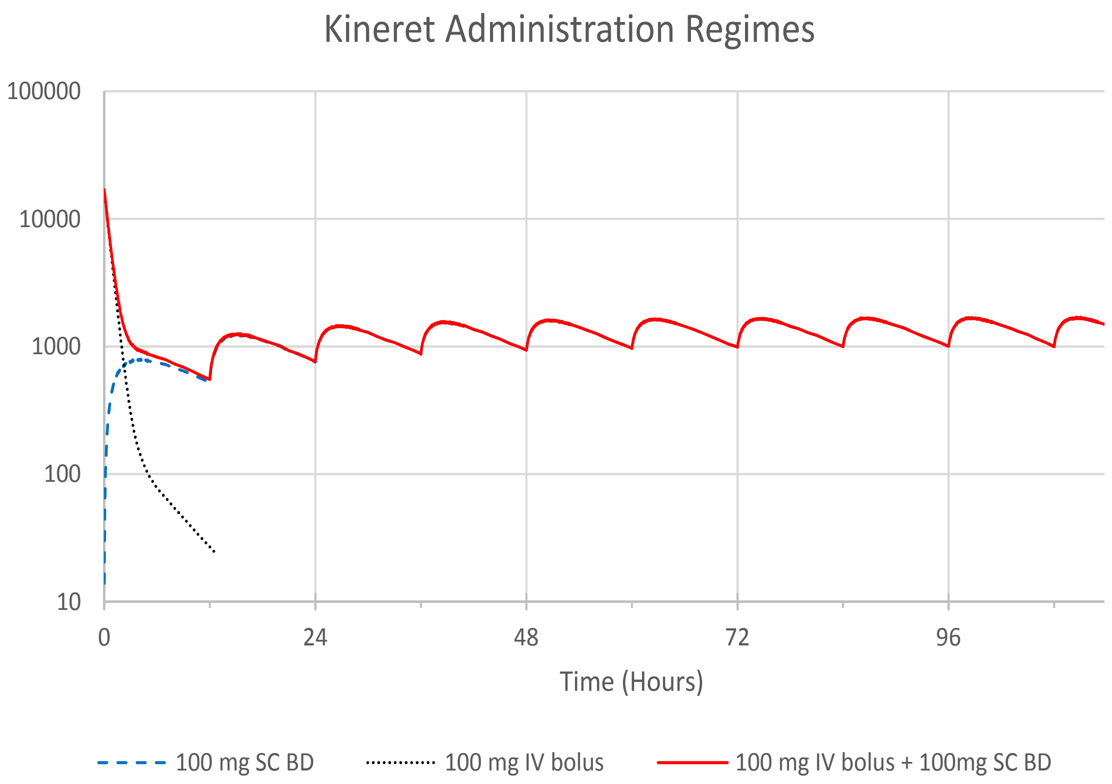

Figure 1. The IASO trial dosing strategy rapidly achieves stable anakinra tissue concentrations in the therapeutic range. (Predicted concentrations of anakinra from simulation data from pharmacokinetic model using trial population data, courtesy Dr J Galea)

We estimate this achieves tissue concentrations of anakinra around 1000ng/ml. Since IL-1 receptor signalling can be stimulated by receptor occupancy levels as low as 2%, large excess concentrations of anakinra are needed to block signalling[14]. Based upon published data demonstrating tissue IL-1 concentrations in UC, our chosen dosing strategy should achieve colonic tissue anakinra concentrations sufficient to antagonise IL-1 signalling and hence reduce or prevent neutrophil activation[5, 15]. This may lead to improvements in patient health at early timepoints following admission.

As part of routine clinical practice for ASUC, treatment with corticosteroids reduces the secretion of both IL-1 and IL-1RA by neutrophils. However, the effect on IL-1RA is greater than on IL-1, skewing the IL-1:IL-1RA ratio towards greater IL-1 availability[16]. This suggests a potential for synergy between corticosteroids and anakinra to reduce total IL-1 levels whilst also rebalancing the IL-1:IL-1RA ratio in favour of the anti-inflammatory IL-1RA.

Monoclonal antibodies are currently in development to target IL-1 signalling. However, in comparison to these, anakinra has a number of advantages:

For peer review only - http://bmjopen.bmj.com/site/about/guidelines.xhtml

- Cessation of drug therapy is followed by rapid elimination of the drug from the plasma over a matter of hours, whereas monoclonal antibodies will take several weeks for elimination increasing the risks in the event of a serious adverse event (SAE) or when giving rescue therapy.
- Anakinra blocks the IL-1 receptor (IL-1R) and therefore antagonises the effects of both forms of biologically active IL-1: IL-1A and IL-1B; anti-IL-1 monoclonal antibodies by their nature must be specific for either IL-1A or IL-1B.
- Finally, anakinra is affordable in contrast to the costs associated with monoclonal antibody development and production.

A trial of anakinra in ASUC is needed now

The initial management of ASUC has changed little since 1955 when, in an early randomised placebo-controlled trial, Truelove and Witts demonstrated that IV corticosteroids reduce the need for colectomy[17]. Despite significant advances in the management of ambulatory patients with UC, patients with ASUC have largely been excluded from clinical studies and the initial therapeutic management of patients after hospitalisation has scarcely changed in 60 years. Major therapeutic advances have been restricted to the development of 'rescue' therapy for those failing treatment with IV corticosteroids. These have been performed in small cohorts (a study of just 20 patients randomised to placebo or IV ciclosporin and a study of 84 patients randomised to infliximab or placebo[18, 19]). The use of 'rescue' therapies has undoubtedly advanced care and is currently necessary in around 50% of patients ([20] and Prof John Williams personal communication from CONSTRUCT trial data). For those receiving rescue therapy, colectomy rates at 3 months remain around 50% (CONSTRUCT trial data). Avoiding surgery, particularly emergency surgery, and reducing the impact of hospitalisation with ASUC has been identified by patients as a key priority[21].

With current standards of care, there is a difficult balance to be struck between the risks of giving expensive or toxic rescue therapy to those who may not need it, versus the risks of delaying effective medical treatment for those who will fail corticosteroid therapy alone. The question therefore arises as to whether we can improve the management of ASUC by doing something different right from the point of initial treatment, rather than waiting for treatment failure? The ideal therapy to test in this regard would be safe, cheap and potentially efficacious. To reduce risk during trials, the therapy should also be capable of being given alongside or even potentially synergistic with steroid therapy. In all these regards, anakinra shows strong potential.

8 Rationale for Trial

A randomised, placebo controlled trial of anakinra in patients with ASUC offers an opportunity to understand whether this drug can benefit patients by impacting upon outcomes in a clinically meaningful manner. Although no formal health economic analysis is planned given the scale and limitations of the trial, the low cost of the trial intervention, and the high cost of the outcomes we are trying to prevent (rescue therapy, prolonged admission, surgery) mean that there is also a strong possibility for potential health economic benefits to arise from a successful trial outcome.

Although anakinra has never been used previously in ASUC, we have adopted an approach of proceeding to an RCT with due consideration of alternatives and with some important safety measures. An alternative would have been to administer the drug on an open-label basis in the first instance. However, interpretation of open-label data without a matched placebo group would be problematic in a condition showing heterogeneity in outcomes between reported studies[22]. Furthermore, anakinra already has a proven safety record when given to large numbers patients, including those on immunosuppressants and with systemic infections. Finally, any patients given

For peer review only - http://bmjopen.bmj.com/site/about/guidelines.xhtml

open-label anakinra would not be able to contribute data to a subsequent RCT due to the absence of blinding. Instead, this trial proceeds directly to a placebo controlled RCT with a planned series of early analyses that will test trial feasibility and detect any evidence of futility (see details below). This allows all patients to contribute data to the final analysis, whilst reducing trial duration, costs and risks to patients.

This trial will involve exposure to anakinra at a dose considerably lower than that used in large scale phase III trials in sepsis (up to 10mg/kg/hr intravenous – i.e. 16.8g/day for a 70kg patient vs the 200mg/day dose in this trial). Despite being performed in critically ill patients, including those with systemic infections, these studies reported no significant adverse safety profile. It should be noted that the licensed route of administration of anakinra is by daily SC injection, whilst in this trial we will use a loading dose given as an IV bolus, followed by twice daily SC injections. Nevertheless, in addition to the much higher IV doses used in the previous phase III sepsis studies mentioned, anakinra has also been used at a range of concentrations given by both intravenous and subcutaneous routes in a number of other trials with a favourable safety profile[9, 10], including inpatient studies adopting a similar twice daily SC injection schedule[23].

In accordance with standard Clinical Trial of Investigational Medicinal Product (CTIMP) design, staff at participating sites will be Good Clinical Practice (GCP) trained, receive trial specific training and be subject to GCP inspections. Day-to-day trial management will be supported by the Trial Management Group (TMG). The trial will be overseen by trial steering committee (TSC) supported by an independent data monitoring committee (iDMC). Due to the potential for a combined immunosuppressant effect of treatment with corticosteroids, anakinra and then rescue therapy, the iDMC will be specifically asked to monitor for infection-related SAEs.

Anakinra will be used in addition to the current standard of care. No currently effective treatments will be withheld or delayed.

Despite the robust scientific rationale and supporting animal data, there are no published reports of the use of anakinra in ASUC. Therefore, the study design will include a series of interim analyses performed in an unblinded manner by the data monitoring committee which will be designed to detect any evidence of (a) harm and (b) futility.

The first interim analysis, designed to assess any major safety signals, will be performed after the first 20 participants have been recruited (feasibility study). A further interim analysis is scheduled after the first 100 participants have been recruited, and is designed to assess for both safety and any evidence of futility. The independent data monitoring committee will be asked to assess the likelihood of the trial producing a negative outcome, and will advise the TSC on the need for early trial termination.

9 Trial Design

9.1 Statement of Design

This phase II trial comprises a two-arm (parallel group), randomised, placebo-controlled, multi-centre, double-blinded study with pre-specified interim analysis for evidence of harm or futility.

A sub-study will also investigate endoscopic response to treatment in both trial arms.

For peer review only - http://bmjopen.bmj.com/site/about/guidelines.xhtml

9.2 Number of Centres

IASO will be a multi-centre study based in UK NHS acute hospitals.
We plan to include approximately 4 centres in the first phase of the trial and 20 centres in the second phase of the trial.

All sites will recruit, treat and follow up participants equally, but a subset of sites will also recruit participants into the optional endoscopic sub-study involving one additional flexible sigmoidoscopy with biopsy collection.

9.3 Number of Participants

We plan to include approximately 214 participants in the trial with 40 participants also included in the optional endoscopic sub-study.

Further details regarding the sample size justifications can be found in Section 17.3.1.

9.4 Participants Trial Duration

Participants' involvement in the trial will last approximately 6 months. This includes an initial treatment period of approximately 5–7 days, during which screening and an investigational medicinal product treatment course of up to 5 days will be completed. After this, there will be a follow up period of approximately 6 months.

Review of participant outcomes following trial participation may continue for up to 5 years using Hospital Episode Statistics (HES) data, without any additional burden to the participant.

9.5 Trial Objectives

IASO is a trial to evaluate whether anakinra is an effective treatment for patients with ASUC. The trial will test whether anakinra can reduce the need for medical or surgical rescue therapy and improve outcomes including colectomy in patients with ASUC when given alongside corticosteroids. The primary aim is to compare the clinical effects of anakinra with placebo when given in addition to current standard care. The secondary aims include assessing the effects of the intervention on disease biology and bowel inflammation, as well as drug safety, and effects on longer term health and patient reported quality of life. The trial also aims to generate scientific data to permit analysis of plasma and tissue markers of response (both to anakinra and to steroids), in particular showing the effects of IL-1 blockade on the mucosal immune system. The trial will seek to validate a transcriptional predictor of outcomes in UC that we have previously reported[24].

9.5.1 Primary objective

The primary objective of this trial is to compare the clinical effects of anakinra with placebo when given in addition to current standard care in patients with acute severe colitis.

9.5.2 Secondary objectives

To compare the effects of anakinra with placebo when given in addition to current standard care to patients with acute severe colitis in terms of:

- Safety
- Need for colectomy
- Patient reported outcomes
- Endoscopic and histologic evidence of treatment effects

For peer review only - http://bmjopen.bmj.com/site/about/guidelines.xhtml

- Evidence of disruption of the IL-1 signalling pathway

9.6 Trial Outcome Measures

9.6.1 Primary outcome measure

Incidence of medical or surgical rescue therapy within 10 days following the commencement of intravenous corticosteroid therapy.

Need for rescue therapy reflects failure to respond to initial treatment and is judged against clinical criteria between 3-5 days after commencement of IV corticosteroid therapy[2] (see Appendix 4 – Treatment decision tree for IASO trial). In line with international guidelines, the initiation of rescue therapy should not be delayed in those with clinical need. A reduction in the need for rescue therapy will reflect evidence of beneficial effects of the intervention. Although rescue therapy may be initiated as early as 3 days following commencement of intravenous corticosteroids, some clinicians may choose to delay this decision to allow more time for a clinical response. The outcome measure will therefore be the proportion of participants in each group who have started rescue therapy within 10 days following the commencement of intravenous corticosteroids.

For the purposes of the primary endpoint analysis, the timepoint of the start of rescue therapy will be as follows:

- Medical rescue therapy: Date and approximate time of first treatment administration
- Surgical rescue therapy: Date and approximate time of start of surgery

9.6.2 Secondary outcome measures

1. *Incidence of Colectomy within 98 days following commencement of IV corticosteroid therapy.*

2. *Burden of disease activity, measured by daily modified Truelove and Witts severity index (MTWSI)[18] scores over Days 1-5 after initial IMP administration.*

3. *Time to clinical response (defined as 2nd consecutive day with MTWSI<10)*

4. *Time to medical or surgical rescue therapy, measured according to the time after the first dose of IV corticosteroids until the time that rescue therapy occurs (using definitions as set out in primary endpoint). Data will be captured up to the same timepoint as the primary endpoint.*

5. *Incidence of SAEs, measured until Day 10 (+3) following* commencement of IMP treatment*.*

9.6.3 Exploratory outcome measure

1. Patient-reported quality of life (EQ-5D[25] and CUCQ-32[26]) at baseline and at 3 and 6 months following commencement of IMP treatment
2. Endoscopic response at Day 3 (± 1 day) (via standardised endoscopic scoring completed by a local and a central blinded assessor) following commencement of IMP treatment (endoscopic sub-study group only)

For peer review only - http://bmjopen.bmj.com/site/about/guidelines.xhtml

In addition to the above exploratory endpoints, the trial will also aim to collect data to perform further exploratory analyses. These will include data related to investigating biomarkers of ASUC (gene and inflammatory cytokine expression), predictors of response (based on IL-1 signalling as well as biochemical and haematological parameters [e.g. haemoglobin, total white blood cell, neutrophil, lymphocyte, monocyte, platelet, albumin and c-reactive protein levels]) and stool microbial characterisation at baseline and at Day 5 (± 1 day).

Furthermore, data from the endoscopic substudy will be investigated to determine the histological response to treatment in addition to determining the expression of inflammatory mediators (e.g. tissue IL-1A, IL-1B, IL-1RA, TNF-α, IL-6, IL-10 levels) at Day 3 (± 1 day) following commencement of IMP treatment. Finally, patient readmission and colectomy data (e.g. via HES) may be examined to investigate the impact of treatment in the longer term.

10 Selection and withdrawal of participants

10.1 Inclusion Criteria

To be included in the trial the participant must:

- Be aged 16–80 years inclusive
- Have given written informed consent to participate
- Be hospitalised with clinically confirmed or suspected ASUC and a MTWSI score ≥11
- Have a requirement for treatment with IV corticosteroids in the judgement of the treating clinician, with the possibility to receive a first dose of IMP within 36 hours of commencement of IV corticosteroids

10.2 Exclusion Criteria

The presence of any of the following will preclude participant inclusion:

- Pregnant or breast-feeding women
- Oral corticosteroid dosing for a duration of 8 weeks or more immediately prior to commencement of IV corticosteroid dosing
- History of severe hepatic impairment (e.g. Child-Pugh = Grade C)
- Moderate or Severe renal impairment (estimated glomerular filtration rate [eGFR] <60ml/min/1.73m^2)
- Neutropenia (neutrophil count <1.5x10^9/l)
- Previous treatment with anakinra for any indication
- Documented hypersensitivity to the active substance or to any of the excipients or to E. coli derived proteins; latex allergy
- Evidence (from blood cultures etc) or clinical suspicion of systemic infection*
- Current or previous cytomegalovirus (CMV) infection requiring treatment with anti-viral agents
- Current treatment with anti-TNF-α therapy or anti-TNF-α discontinuation within previous 16 weeks
- A history of pulmonary TB infection
- Any absolute contraindication to IV corticosteroid
- History of malignancy (with the exception of non-melanoma skin cancer) or colonic dysplasia
- Rectal therapy in previous 14 days (sub-study exclusion only)
- Receipt of another IMP as part of a CTIMP within the previous 16 weeks

*concurrent prescription of antibiotics to cover for the possibility of GI infection whilst awaiting stool culture results, or the possibility of bacterial translocation relating to

For peer review only - http://bmjopen.bmj.com/site/about/guidelines.xhtml

severe colitis, is not an exclusion criterion where the physician suspects ulcerative colitis is the most likely diagnosis.

10.3 Patient Trial ID and Treatment Assignment

All patients screened for the trial will be assigned a unique participant ID number. Suitable participants will subsequently be randomised (1:1) to drug:placebo using an online randomisation system accessible via password-protected access. Randomisation will be stratified for:

- Previous or current therapy with any of: immunomodulators (azathioprine, 6-mercaptopurine, 6-thioguanine, methotrexate, ciclosporin) biologics (anti-TNF-ɑ monoclonal antibodies, anti-adhesion molecule antibodies, other anti-cytokine antibodies) or oral janus kinase inhibitors
- Current or previous oral corticosteroid prescription(s) within 8 weeks prior to first dose of intravenous corticosteroids

Access to the web-based randomisation system (Sealed Envelope) will be via individual user accounts provided to the principal investigator (PI) and suitably trained and delegated members of the research team at each site as appropriate. Immediate allocation of treatment will be performed, with documentation of the decision in a confirmatory email. The system will allocate the participant a treatment code which will relate uniquely to a supply of IMP (complete pack for up to 5 days of treatment) that is held at that trial site.

10.4 Method of Blinding

Trial participants and research teams will be blinded to the treatment group for the duration of the trial. Site pharmacies will also be blinded. The physical appearance of the IMPs is matched and the IMPs will be presented in identical packaging. SC administration of the trial drug has been associated with injection site reactions. The incidence of reactions is reduced by pre-warming the pre-filled syringe prior to injection by holding the syringe in the hand, which will form part of the administration instructions. It is important to note that injection site reactions also occur with the placebo, since, in part, the reaction relates to the carrier buffer[27]. This greatly reduces the risk of accidental unblinding.

10.5 Participant Withdrawal Criteria

There are two types of withdrawal in this trial:

- Treatment termination – participant withdraws from IMP administration
- Trial Withdrawal – participant withdraws from the trial in its entirety

Participants may choose to withdraw from treatment or the full study at any stage without prejudice to standard clinical care.

10.5.1 Treatment termination

Clinicians should cease treatment with the study drug according to their judgement or under any of the following criteria:

- Withdrawal of consent for treatment administration
- Development of renal impairment (eGFR <60 ml/min/1.73m^2) or severe hepatic impairment (Child Pugh grade C)
- Development of neutropenia (neutrophil count <1.5x10^9/l)
- Detection of a significant GI pathogen in stool specimen
- Sufficient recovery to allow for cessation of IV steroids
- Hospital discharge due to recovery
- Decision to commence medical or surgical rescue therapy

For peer review only - http://bmjopen.bmj.com/site/about/guidelines.xhtml

- CMV reactivation necessitating treatment with anti-viral agents (in the judgement of treating clinicians)
- The development of a SAE necessitating termination of IMP treatment.

Where possible, trial assessments will continue following treatment termination, with the exception of the withdrawal conditions described in Section 10.5.2.

Participants who undergo sufficient recovery to be discharged from hospital will not be assessed daily but will be followed up as normal. Participants who undergo a colectomy during their inpatient stay will undergo assessments as planned, with the exception of the MTWSI score.

10.5.2 Trial withdrawal

In addition to the treatment termination criteria, the following criteria would result in participant withdrawal from the trial, with no further assessments being performed:

- Withdrawal of consent for further assessments and data collection
- Death

All participants who received at least one dose of IMP and are withdrawn from the trial will have their outcomes reported as part of the main findings.

Due to the short nature of the intervention, withdrawal rates are expected to be low.

The iDMC will regularly review all safety data and consider whether there is evidence of harm that might necessitate recommending changes to the trial protocol or trial termination. In addition, there will be 2 pre-specified analyses during the trial. The iDMC will consider performance of the trial against pre-specified criteria, and whether to recommend trial termination.

11 Trial Treatments

11.1 Treatment Summary

For the purpose of this trial, the following are considered the IMP(s):

- Anakinra solution for injection
- Placebo to match anakinra solution for injection

11.1.1 Name and description of the IMP

Anakinra (brand name Kineret) is a clear, colourless to white solution for injection which may contain some translucent-to-white particles of protein in the solution. The placebo solution will have the same physical appearance. Both active and placebo treatments will be supplied in commercial pre-filled syringe configurations (100mg/0.67ml), packed into trial-specific participant kits, each containing 11 pre-filled syringes. The syringes and kits will have blinded labelling conforming to regulatory requirements and be identified by unique pack (kit) numbers.

11.1.1.1 *Legal status*

Anakinra is a licensed drug in the UK for the treatment of rheumatoid arthritis and several other indications under the Cryopyrin-Associated Periodic Syndromes (CAPS) grouping and is administered subcutaneously. Within this study, the IMP is being used in a new indication (ASUC), dosage regimen and for the loading dose, administered by a currently unlicensed route (IV bolus dose).

For peer review only - http://bmjopen.bmj.com/site/about/guidelines.xhtml

The trial is being carried out under a Clinical Trial Authorisation. The drug is therefore only to be used by the named investigators, for the participants specified in this protocol, and within the trial.

11.1.1.2 *Supply*

IMP supply to sites will be overseen by the trial co-ordinator and distributed by the Royal Free Hospital, London. Upon initial authorisation by the sponsor, an initial supply will be sent to sites; supplies thereafter will be requested and distributed as detailed in the pharmacy manual. IMP will be distributed and stored as per requirements of the SmPC for Anakinra.

IMP for the trial will be provided free-of-charge for participating sites.

11.1.1.3 *Packing and Labelling*

IMP will be ordered using trial-specific prescriptions stating the kit number to dispense. Participant-specific details will be added to labelling at point of dispensing.

11.1.1.4 *Storage conditions*

IMP will be stored in accordance with the requirements of the SmPC for Anakinra.

11.1.1.5 *Maximum duration of treatment of a participant*

The maximum amount of time a participant will be receiving the IMP will be 5 days (a total of 1 IV and 10 SC doses).

11.1.1.6 *Dose*

Initial IV dose – 100mg
Subsequent SC dose – 100mg for up to 10 doses as per administration schedule in Section 11.1.1.7.

11.1.1.7 *Administration*

Day 0

On the first day of trial medication administration (Day 0):

- IV loading dose of 100 mg IMP
- 100 mg IMP SC twice daily (BD), approximately 12 hours apart
  - The first SC dose is given immediately following the IV loading dose
  - In the event that the first dose of IMP occurs after noon on Day 0, only one 100 mg SC dose will be given that day. Full BD dosing will commence the following day and continue as indicated below for Days 1-5.

Administration of the first dose of IMP will only be performed in the following circumstances:

- The time of administration of the first IMP dose falls no later than 36 hours following the time of first dose of IV corticosteroid.
- IV corticosteroid therapy remains ongoing at the time of administration of the first IMP dose (i.e. that a decision to discontinue corticosteroids or transition to oral steroids has not been made after the time of participant consent and before the time of the administration of the first IMP dose).
- A firm decision to start rescue therapy or perform emergency colectomy has not already been taken.

For peer review only - http://bmjopen.bmj.com/site/about/guidelines.xhtml

Day 1-5

During the days subsequent to the first dose of trial medication, the following dosing schedule will be followed:

- BD administration of 100 mg IMP SC
  - For those participants who received two 100 mg IMP SC doses on Day 0, no administration will occur on Day 5.
  - For those participants who received only one SC dose on Day 0, the administration of the final dose of 100 mg IMP SC will occur on Day 5.

Dosing will continue until the earliest of:
- 5 days (a total of 1 IV and 10 SC doses)
- Fulfilment of the treatment termination criteria specified in Section 10.5.

11.1.1.8 *Known drug reactions*

Known drug reactions for anakinra are summarised in Section 4.8 of the SmPC for anakinra (Kineret) approved by the MHRA for use in this trial.

Interactions between anakinra and other medicinal products have not been investigated in formal studies. In clinical trials, interactions between anakinra and other medicinal products (including nonsteroidal anti-inflammatory medicinal products, corticosteroids, and DMARDs) have not been observed.

Concurrent anakinra and TNF antagonist treatment

In a clinical trial with RA patients receiving background methotrexate, participants treated with anakinra and etanercept were observed to have a higher rate of serious infections (7%) and neutropenia than participants treated with etanercept alone and higher than observed in previous trials where anakinra was used alone. The concurrent use of anakinra with etanercept or any other TNF antagonist is not recommended.

Within the study, current treatment with anti-TNF-ɑ therapy or anti-TNF-ɑ discontinuation within previous 16 weeks is an exclusion criterion. Where rescue therapy with anti-TNF-ɑ therapy is required, anakinra will be discontinued with rapid elimination expected from the pharmacokinetic characteristics outlined in Section 7.2 above.

Cytochrome P450 Substrates

The formation of cytochrome p450 (CYP450) enzymes is suppressed by increased levels of cytokines (e.g., IL-1) during chronic inflammation. Thus, it may be expected that for an IL-1 receptor antagonist, such as anakinra, the formation of CYP450 enzymes could be normalized during treatment. This would be clinically relevant for CYP450 substrates with a narrow therapeutic index (e.g. warfarin and phenytoin). Upon start or end of anakinra treatment in patients on these types of medicinal products, it may be relevant to consider therapeutic monitoring of the effect or concentration of these products and the individual dose of the medicinal product may need to be adjusted.

Participants in IASO will be acutely unwell in an inpatient setting and only treated with anakinra for a short duration; routine therapeutic monitoring would be standard practice for appropriate concomitant drugs.

For peer review only - http://bmjopen.bmj.com/site/about/guidelines.xhtml

11.1.1.9 *Dose modifications*

No dosage modifications are permitted. The IMP course may be stopped before all 10 subcutaneous doses have been completed, as described in Section 10.5.

11.1.1.10 *Procedures for monitoring treatment compliance*

Participants are treated as inpatients with all IMP being administered by healthcare professionals. Compliance with treatment may be assessed by referring to medication administration records and any remaining unused IMP after treatment has been stopped, which should be returned to the site pharmacy for reconciliation.

11.1.1.11 *Placebo/Active comparator products*

Placebo to match anakinra.

11.2 Non Investigational Medicinal Products

IV methylprednisolone or hydrocortisone, as per local guidelines for ASUC standard care. As these form part of standard care, IV corticosteroids will not be provided to participating sites as part of the trial.

The trial pharmacy manual should be consulted for further details regarding nIMP procedures.

11.3 Concomitant Therapy

Participants will continue to be treated with routine concomitant medications for other co-morbidities as appropriate within their current clinical condition, with modifications e.g. Oral to IV switches, or temporary cessation as necessary. Appropriate supportive care may also be prescribed, including fluids, nutritional supplements and other medications as necessary.

11.4 Emergency unblinding

In the event of a valid medical or safety reason (e.g. in the case of an SAR where it is necessary for the investigator or treating health care professional to know which treatment the participant is receiving before the participant can be treated), the responsibility to break the treatment code resides solely with the treating clinician.

The online Sealed Envelope randomisation system will also be used for emergency unblinding. Appropriately trained and delegated site staff will be given the necessary access rights and permissions to access this facility. The name, contact details of the unblinder and reason for unblinding will be recorded within the Sealed Envelope system.

The unblinder will not be shown the treatment allocation on-screen. Instead the allocation will be sent by email or fax and should be printed and retained confidentially within the ISF. An email stating that an unblinding has taken place will be automatically sent to the coordination team for oversight purposes.

Wherever possible, members of the research team should remain blinded.

11.5 Accountability and dispensing

Sites will maintain a full accountability record documenting receipt, storage, dispensing, administration, return of unused IMP and used and unused IMP packaging as appropriate, as specified in the pharmacy manual.

For peer review only - http://bmjopen.bmj.com/site/about/guidelines.xhtml

There will no accountability records maintained for nIMPs listed in section 11.2.

12 Procedures and assessments

12.1 Participant identification

We will recruit inpatients with ASUC. Identification of potential participants will be undertaken by the clinical team. Consenting and screening of potential trial participants will be performed at each participating site by trial investigators, or by a suitably qualified and delegated member of the trial team.

All patients who are interested in learning more about the trial will be provided with the approved patient information sheet and be invited to consent to join the trial as soon as possible after admission. Anonymised data on all participants who are approached will be collated in accordance with Consolidated Standards of Reporting Trials (CONSORT) guidelines.

12.2 Consent

The Informed Consent form must be approved by the REC and must be in compliance with GCP, local regulatory requirements and legal requirements. The investigator or designee must ensure that each trial participant is fully informed about the nature and objectives of the trial and possible risks associated with their participation.

The investigator or designee will receive written informed consent from each participant before any trial-specific activity is performed. The informed consent form used for this trial and any change made during the course of this trial, must be prospectively approved by the REC. The investigator will retain the original of each participant signed informed consent form.

Should a participant require a verbal translation of the trial documentation by a locally approved interpreter/translator, it is the responsibility of the individual investigator to use locally approved translators.

Any new information which becomes available, which might affect the participant's willingness to continue participating in the trial will be communicated to the participant as soon as possible.

12.2.1 Sub-study Consent

A total of 40 individuals recruited at 4 centres will be asked to join an optional scientific sub-study cohort. These patients will be provided with the approved sub-study patient information sheet. As part of this they will be asked to consent to an additional flexible sigmoidoscopy at Day 3 (± 1 day) after the initiation of IMP treatment with biopsy collection. Participants can decline to participate in the sub-study but still participate in the main study.

Biopsies will be stored in pseudoanonymised form in compliance with the terms of the Human Tissue Act (2004) for the duration of the trial. If a participant withdraws consent from the scientific sub-study and requests that their samples do not undergo analysis, then any biopsy material not already analysed at this stage can be destroyed. Patients who initially consent to the sub-study but do not undergo the flexible sigmoidoscopy (e.g. due to withdrawal of consent or hospital discharge prior to the procedure) will be replaced until a total of 40 trial participants have undergone the procedure.

For peer review only - http://bmjopen.bmj.com/site/about/guidelines.xhtml

12.3 Screening evaluation

12.3.1 Screening Assessments

Trial specific assessments will only be conducted after participants have given written informed consent.

Screening will be performed based upon the inclusion/exclusion criteria outlined in Section 10. Eligibility will be confirmed by the trial investigator or suitably qualified and delegated member of the trial team. The tests required for screening all form part of standard clinical care, therefore no additional tests will be performed other than a urine β-human chorionic gonadotropin (β-hCG) test for females where there is a possibility of pregnancy.
The screening process will therefore involve:

- Review of the patient's medical record to confirm eligibility in line with the inclusion/exclusion criteria
- Urine β-hCG, for women of childbearing potential

12.3.2 Participant Randomisation

Participants will be randomised by a suitably trained and delegated member of the research team as described in section 10.3 and the randomisation manual

12.4 Baseline Assessments

The following activities will be performed and recorded at baseline:

- Medical history review
- Review of blood parameters (haemoglobin, total white blood cell, neutrophil, lymphocyte, monocyte, platelet, albumin and c-reactive protein) following a blood test performed as part of standard care
- 3 research blood samples (1 plasma tube, 1 nucleic acid tube, 1 genotyping tube) as described in the laboratory manual
- Concomitant medications review
- Demographics
- MTWSI assessment
- Stool sample collection
- EQ-5D and CUCQ-32 questionnaires (Appendix 4 – EQ-5D and CUCQ-32 Questionnaires)

12.5 Trial assessments

The below procedures will all occur during the inpatient admission. Participants will be managed in line with standard clinical guidelines for ASUC[2], as set out in the trial decision tree (Appendix 4 – Treatment decision tree for IASO trial
).

Wherever possible, research blood samples will be taken at the same time as a standard care blood samples. Safety monitoring in the trial will be conducted according to the steps described in Section 13. It should be noted that both Baseline and, subsequently, Day 0 may occur on the same calendar date.

Day 0

Prior to the first administration of IMP, the following assessments will be performed:

- Review of adverse events
- Recording of any changes to concomitant medication since the last assessment

For peer review only - http://bmjopen.bmj.com/site/about/guidelines.xhtml

- Confirmation that IMP administration can proceed, according to the following criteria:
    - The time of administration of the first IMP dose falls no later than 36 hours following the time of first dose of IV corticosteroid.
    - IV corticosteroid therapy remains ongoing at the time of administration of the first IMP dose (i.e. that a decision to discontinue corticosteroids or transition to oral steroids has not been made after the time of participant consent to participate in the trial and before the time of the administration of the first IMP dose).
    - A firm decision to start rescue therapy or perform emergency colectomy has not already been taken
- IMP administration

Trial assessments will be performed as normal in the event that a participant was randomised but did not receive IMP.

Day 1-5

During the days subsequent to the first dose of trial medication, the following activities will be recorded for each day up to and including Day 5:

- MTWSI assessment / review of standard care clinical MTWSI assessment domains[#]
- Recording of any changes to concomitant medication prescription since the last assessment
- Review of blood parameters (haemoglobin, total white blood cell, neutrophil, lymphocyte, monocyte, platelet, albumin and c-reactive protein) following blood tests performed as part of standard care
- 2 research blood samples (1 plasma tube, 1 nucleic acid tube)*
- Review of adverse events
- Medical or surgical (emergency colectomy) rescue therapy assessment

[#] *This will not be recorded for patients who have undergone colectomy*

**A minimum of 1 sample every 72 hours should be taken. Wherever possible every effort should be made to obtain the samples every day (up to 10 samples).*

Where possible, trial assessments will continue following treatment termination, with the exception of the withdrawal conditions described in Section 10.5.2. In the event of hospital discharge prior to Day 5, the above activities will not be recorded beyond the timepoint of discharge.

Day 3 (sub-study only) ± 1 day

In addition to the daily assessments described above for Days 1-5, a scientific sub-study group of 40 participants will undergo an additional optional flexible sigmoidoscopy (without bowel preparation) on Day 3 (± 1 day). This will be performed during the index inpatient admission.

Findings will be recorded according to a standardised endoscopic scoring system (Mayo) by blinded local and central assessors. Histology will be scored on up to a maximum of

For peer review only - http://bmjopen.bmj.com/site/about/guidelines.xhtml

6 biopsies taken from each of the rectum and sigmoid colon, according to validated scoring systems for colitis severity at the end of the trial [28, 29]' [30].

Where possible, endoscopic scores, text reports and photographic recordings of sigmoidoscopies will be sent to the central trial team for verification. In addition, where available, data collected as part of standard care (non sub-study) endoscopies will be used to supplement trial-specific data. These will be scored by the blinded central assessor.

__Day 5__

In addition to the daily assessments described above for Days 1-5, a stool sample will also be collected on Day 5 (± 1 day).

__Follow Up Period__

Following the participants' inpatient stay, the following assessments will occur:

- Day 10 (+3) following commencement of IMP treatment – Rescue therapy assessment*
- Day 10 (+3) following commencement of IMP treatment – AE monitoring, as described in Section 13
- Day 98 (+ 14) following commencement of IMP treatment – colectomy assessment†
- Approximately 3 months following commencement of IMP treatment – Participants will be contacted by post and asked to complete the EQ-5D and CUCQ-32 questionnaires.
- Approximately 6 months following commencement of IMP treatment – EQ-5D and CUCQ-32 questionnaires.

*If a participant proceeds to rescue therapy or colectomy during the admission, this will be recorded in the medical records and the CRF. In the event that a participant is discharged prior to Day 10 without having started rescue therapy, a member of the trial team will contact the participant on (or within 3 days following) Day 10 to confirm that the participant remains out of hospital and has not received rescue therapy elsewhere since discharge. If the participant is not contactable, local hospital records will be used to check for admission.

†If not previously recorded, a member of the trial team will contact the participant on Day 98 (+14) to confirm that the participant remains out of hospital and has not undergone a colectomy since discharge. If the participant is not contactable, local hospital records will be used to check for admission.

Participant compliance is expected to be high and loss-to-follow up minimal.

The 3 & 6 month questionnaires will be sent to the participants from the central research team based in Cambridge by post. If a participant fails to return the questionnaire a member of the local research team will contact the participant by telephone after 4 and 8 weeks. If required, questionnaires can be re-sent by the central research team as appropriate. Any 3-month questionnaires not received within 12 weeks of being sent to a participant will be marked as missing. However, unless

For peer review only - http://bmjopen.bmj.com/site/about/guidelines.xhtml

requested otherwise by the participant, the subsequent 6 month questionnaires will still be sent to the participant for completion.

For peer review only

For peer review only - http://bmjopen.bmj.com/site/about/guidelines.xhtml

12.6 Schedule of Assessments

**Assessments**	**Treatment phase**				**Follow up phase**			
	Screening	Baseline	Day 0	Assessment Days 1 - 5 (1)	Day 10 (+3 days)	Day 98 (+ 14 days)	~3 Months	~6 Months
Informed consent	✓							
Eligibility assessments	✓							
Urine β-hCG*	✓							
Randomisation	✓							
Medical history review		✓						
Socio-demographic data		✓						
Questionnaires (EQ-5D, CUCQ-32)		✓					✓	✓
Review of standard-care blood parameters		✓		✓				
Collection of 3 research blood samples		✓						
Collection of 2 research blood samples				✓				
Daily MTWSI assessment / review of standard care clinical MTWSI assessment domains#		✓		✓				
Stool Sample Collection		✓		✓(2)				
Concomitant medications (full list)		✓						
Concomitant medications (changes since last assessment)			✓	✓				
Review of adverse events			✓	✓	✓			
Confirmation of continued eligibility to receive IMP and that IMP administration falls within 36 hour window			✓					
Rescue therapy (medical or surgical) assessment				✓	✓(5)			
Colectomy assessment				✓		✓(5)		
**Sub-study-Only Assessments**								
Informed consent	✓							
Flexible sigmoidoscopy and ~12x pinch biopsy collection*				✓(3)				
**Administration Schedule**								
IMP administration – 100 mg IV			✓					
IMP administration – 100 mg SC BD			✓	✓(4)				

(1) – Assessments will continue until hospital discharge/withdrawal/death. Treatment will continue until hospital discharge/treatment termination criteria have been met
(2) – Assessment Day 5 only ± 1day
(3) – Assessment Day 3 only ± 1day
(4) – Dosing at Day 5 (representing the 10th BD SC dose) will only occur in the event that a participant received their 1st overall SC dose in the latter part of Day 0.
(5) – If information not already collected.
*for women of childbearing potential,
#MTWSI score will not be calculated for participants that have undergone colectomy

For peer review only - http://bmjopen.bmj.com/site/about/guidelines.xhtml

12.7 Long-Term Follow-up Assessments

Participant status following discharge, including readmissions and subsequent surgery may be assessed using patient-specific HES data from the NHS Digital, Data Access Request Service. Any data collection would start at 1 year post-commencement of IMP treatment and examination of outcomes via HES data would potentially continue for up to 5 years, depending on study outcomes. A yearly assessment of HES outcomes by the TSC would determine whether collection of HES data should continue.

12.8 End of Trial Participation

Participants will return to their normal standard of care as defined by their local physicians, following completion of trial treatment.

12.9 Trial restrictions

Live vaccinations should not be given during the treatment period. Live vaccinations can be given after the end of treatment period as soon as the participant is judged otherwise well enough to receive the vaccine.

Contraception use or abstinence will not be mandated as participants will be hospital in-patients with an acute severe medical condition. The drug is rapidly metabolised and will be fully eliminated from the circulation within 24 hours of drug cessation. The drug is not genotoxic. Therefore no longer term contraceptive measures are required in participants of either sex.

13 Assessment of Safety

13.1 Definitions

13.1.1 Adverse event (AE)

Any untoward medical occurrence in a participant or clinical trial participant administered a medicinal product and which does not necessarily have a causal relationship with this treatment.
An adverse event can therefore be any unfavourable and unintended sign (including an abnormal laboratory finding), symptom, or disease temporally associated with the use of an investigational medicinal product, whether or not considered related to the investigational medicinal product.

Please note: Recording of all adverse events must start from the point of Informed Consent regardless of whether a participant has yet received a medicinal product.

13.1.2 Adverse reaction to an investigational medicinal product (AR)

All untoward and unintended responses to an investigational medicinal product related to any dose administered. All adverse events judged by either the reporting investigator or the sponsor as having a reasonable causal relationship to a medicinal product qualify as adverse reactions. The expression reasonable causal relationship means to convey in general that there is evidence or argument to suggest a causal relationship.

13.1.3 Unexpected adverse reaction

An adverse reaction, the nature, or severity of which is not consistent with the applicable reference safety information (RSI).

When the outcome of the adverse reaction is not consistent with the applicable RSI this adverse reaction should be considered as unexpected.

For peer review only - http://bmjopen.bmj.com/site/about/guidelines.xhtml

The term "severe" is often used to describe the intensity (severity) of a specific event. This is not the same as "serious," which is based on participant /event outcome or action criteria.

13.1.4 Serious adverse event or serious adverse reaction (SAE / SAR)

Any untoward medical occurrence that at any dose:

- Results in death
- Is life-threatening
- Requires hospitalisation or prolongation of existing inpatients´ hospitalisation
- Results in persistent or significant disability or incapacity
- Is a congenital anomaly or birth defect.
- Is an important medical event - Some medical events may jeopardise the participant or may require an intervention to prevent one of the above characteristics/ consequences. Such events (hereinafter referred to as 'important medical events') should also be considered as 'serious'

Life-threatening in the definition of a serious adverse event or serious adverse reaction refers to an event in which the participant was at risk of death at the time of event; it does not refer to an event which hypothetically might have caused death if it were more severe.

13.1.5 Suspected Unexpected Serious Adverse Reaction (SUSAR)

A serious adverse reaction, the nature and severity of which is not consistent with the information set out in the Reference Safety Information

13.1.6 Reference Safety Information (RSI)

A list of medical events that defines which reactions are expected for the IMP within a given trial and thus determining which Serious Adverse Reactions (SARs) require expedited reporting.

For this trial the Reference Safety Information is: Section 4.8 of the SmPC for anakinra (Kineret), updated 31 March 2016 approved by the MHRA for use in this trial

13.2 Expected Adverse Reactions/Serious Adverse Reactions (AR /SARs)

All expected Adverse Reactions are listed in the latest MHRA approved version of the RSI as specified in section 13.1.6. This must be used when making a determination as to the expectedness of the adverse reaction. If the adverse reaction meets the criteria for seriousness, this must be reported as per section 13.5.

13.3 Expected Adverse Events/Serious Adverse Events (AE/SAE)

The following AEs are common and well-recognised side effects associated with administration of the IMP. They are generally not serious in nature and will not be recorded in the AE/AR Log as part of this trial:

- Injection site and mild skin reactions following SC injection of IMP (anakinra or matched placebo)

The following AEs, which may fall under the definition of SAEs as indicated in Section 13.1, are expected to occur commonly as a result of disease progression, rather than trial participation:

- Diarrhoea/worsening diarrhoea
- Abdominal pain/increased abdominal pain
- Rectal pain/worsening rectal pain

For peer review only - http://bmjopen.bmj.com/site/about/guidelines.xhtml

- Development of toxic megacolon
- Development of hypoalbuminaemia

The above signs and symptoms are expected during disease progression necessitating rescue therapy (medical or surgical). As these events are common and expected outcomes, they will be exempt from SAE reporting (as outlined in Section 13.5), however details will be collected in the CRF. In addition, the decision to prescribe medical rescue therapy or perform emergency colectomy will not need SAE reporting. All instances of medical and surgical rescue therapy will still be collected via the trial outcome data and provided to the iDMC for review.

13.4 Evaluation of adverse events

The Sponsor expects that adverse events are recorded from the point of Informed Consent regardless of whether a participant has yet received a medicinal product. Individual adverse events should be evaluated by the investigator or delegate. This includes the evaluation of its seriousness, and any relationship between the investigational medicinal product(s) and/or concomitant therapy and the adverse event (causality).

13.4.1 Assessment of seriousness

Seriousness is assessed against the criteria in section 13.1.4.This defines whether the event is an adverse event, serious adverse event or a serious adverse reaction

13.4.2 Assessment of causality

Definitely: A causal relationship is clinically/biologically certain. **This is therefore an Adverse Reaction**

Probably: A causal relationship is clinically / biologically highly plausible and there is a plausible time sequence between onset of the AE and administration of the investigational medicinal product and there is a reasonable response on withdrawal. **This is therefore an Adverse Reaction.**

Possibly: A causal relationship is clinically / biologically plausible and there is a plausible time sequence between onset of the AE and administration of the investigational medicinal product. **This is therefore an Adverse Reaction.**

Unlikely: A causal relation is improbable and another documented cause of the AE is most plausible. **This is therefore an Adverse Event.**

Unrelated: A causal relationship can be definitely excluded and another documented cause of the AE is most plausible. **This is therefore an Adverse Event.**

Unlikely and Unrelated causalities are considered NOT to be trial drug related
Definitely, Probably and Possibly causalities are considered to be trial drug related

A pre-existing condition must not be recorded as an AE or reported as an SAE unless the condition worsens during the trial and meets the criteria for reporting or recording in the appropriate section of the CRF.

13.4.3 Clinical assessment of severity

Mild: The participant is aware of the event or symptom, but the event or symptom is easily tolerated

Moderate: The participant experiences sufficient discomfort to interfere with or reduce his or her usual level of activity

For peer review only - http://bmjopen.bmj.com/site/about/guidelines.xhtml

Severe: Significant impairment of functioning; the subject is unable to carry out usual activities and / or the participant's life is at risk from the event.

13.4.4 Recording of adverse events

Adverse events and adverse reactions should be recorded in the medical notes and the appropriate section of the CRF. Serious Adverse Events and Serious Adverse Reactions should be reported to the sponsor as detailed in section 13.5.

13.5 Reporting serious adverse events

Each Principal Investigator needs to record all adverse events and report serious adverse events to the Chief Investigator using the trial specific SAE form within 24 hours of their awareness of the event.

The Chief Investigator is responsible for ensuring the assessment of all SAEs for expectedness and relatedness is completed and the onward notification of all SAEs to the Sponsor immediately but not more than 24 hours of first notification. The sponsor has to keep detailed records of all SAEs reported to them by the trial team.

The Chief Investigator is also responsible for prompt reporting of all serious adverse event findings to the MHRA if they could:

- Adversely affect the health of participants
- Impact on the conduct of the trial
- Alter the risk to benefit ratio of the trial
- Alter the competent authority's authorisation to continue the trial in accordance with Directive 2001/20/EC

The completed SAE form can be faxed or emailed. Details of where to report the SAE's can be found on the IASO SAE form and the front cover of the protocol. SAE information will be shared with the Sobi Drug Safety Department.

The IMP has a short half-life (<6h). No cumulative or late effects are anticipated. For this reason, recording of AEs and reporting of SAEs will be actively monitored up to Day 10 (+3 days). Following the end of the active monitoring period, investigators are still required to report and SARs or SUSARs of which they become aware.
Where a participant withdraws consent for further processing of data, this does not preclude the reporting of SARs and SUSARs which are required to continue being reported according to the protocol for regulatory purposes. All participants must be followed-up until SAE, SAR & SUSAR resolution in all instances.

13.6 Reporting of Suspected Unexpected Serious Adverse Reactions (SUSARs)

All suspected adverse reactions related to an investigational medicinal product (the tested IMP and comparators) which occur in the concerned trial, and that are both unexpected and serious (SUSARs) are subject to expedited reporting. Please see section 13.1.6 for the Reference Safety Information to be used in this trial.

13.6.1 Who should report and whom to report to?

The Sponsor delegates the responsibility of notification of SUSARs to the Chief Investigator. The Chief Investigator must report all the relevant safety information previously described, to the:

- Sponsor
- Competent authorities in the concerned member states (e.g. MHRA)
- Ethics Committee in the concerned member states

For peer review only - http://bmjopen.bmj.com/site/about/guidelines.xhtml

The Chief Investigator shall inform all investigators concerned of relevant information about SUSARs that could adversely affect the safety of participants.

13.6.2 When to report?

13.6.2.1 Fatal or life-threatening SUSARs

All parties listed in 13.6.1 must be notified as soon as possible but no later than **7 calendar days** after the trial team and Sponsor has first knowledge of the minimum criteria for expedited reporting.

In each case relevant follow-up information should be sought and a report completed as soon as possible. It should be communicated to all parties within an additional **8 calendar days**.

13.6.2.2 Non-fatal and non-life-threatening SUSARs

All other SUSARs and safety issues must be reported to all parties listed in 13.6.1 as soon as possible but no later than **15 calendar days** after first knowledge of the minimum criteria for expedited reporting. Further relevant follow-up information should be given as soon as possible.

13.6.3 How to report?

13.6.3.1 Minimum criteria for initial expedited reporting of SUSARs

Information on the final description and evaluation of an adverse reaction report may not be available within the required time frames for reporting. For regulatory purposes, initial expedited reports should be submitted within the time limits as soon as the minimum following criteria are met:
a) A suspected investigational medicinal product
b) An identifiable participant (e.g. trial participant code number)
c) An adverse event assessed as serious and unexpected, and for which there is a reasonable suspected causal relationship
d) An identifiable reporting source

and, when available and applicable:

- An unique clinical trial identification (EudraCT number or in case of non-European Community trials the sponsor's trial protocol code number)
- An unique case identification (i.e. sponsor's case identification number)

13.6.3.2 Follow-up reports of SUSARs

In case of incomplete information at the time of initial reporting, all the appropriate information for an adequate analysis of causality should be actively sought from the reporter or other available sources. Further available relevant information should be reported as follow-up reports.
In certain cases, it may be appropriate to conduct follow-up of the long-term outcome of a particular reaction.

13.6.3.3 Format of the SUSARs reports

Electronic reporting is the expected method for expedited reporting of SUSARs to the competent authority. The format and content as defined by the competent authority should be adhered to.

For peer review only - http://bmjopen.bmj.com/site/about/guidelines.xhtml

13.7 Pregnancy Reporting

Pregnancy reporting is only required for trial participants whilst they are receiving IMP and for up to 24 hours post their last dose.

14 Toxicity/Reaction Management

Anaphylaxis: intravenous chlorphenamine 10mg (Piriton), hydrocortisone 200mg, and rarely adrenaline, will be given in line with local procedures for anaphylaxis.

Injection site reactions are relatively common with both IMP and placebo, but can be minimised by warming the syringe before injection. If necessary, application of a cold pack to the injection site 2–3 min before and immediately after the injection can reduce acute pain associated with injections. Paracetamol may also be used to help with pain. Delayed skin reactions can be managed by topical application of 1% hydrocortisone or anti-histamine creams. It is recommended to alternate the injection sites to avoid recall reactions.

15 Evaluation of Results (Definitions and response/evaluation of outcome measures)

MTWSI will be recorded along with other patient reported outcomes and laboratory measures, as detailed in the CRF.

15.1 Response criteria

Treatment response according to MTWSI will be defined as 2 consecutive days with MTWSI <10.

16 Storage and Analysis of Samples

Blood samples will be collected and centrifuged according to the laboratory manual. Following centrifugation, plasma should be apportioned into clean, labelled tubes prior to storage in a -20°C to -80 freezer.

Nucleic acid and genotyping tubes will be labelled and placed in storage in a –20°C to -80 freezer.

Stool samples will be collected, handled and preserved according to the laboratory manual, prior to storage.

Biopsy samples (endoscopic sub-study only) will be collected from the rectum and sigmoid colon as detailed in section 12.5. From *each* location, up to 6 biopsies will be taken and distributed into storage reagents according to the laboratory manual.

The study coordinator will liaise with sites to coordinate batch shipment of samples in secure, protective packaging to the receiving lab at Addenbrooke's Hospital. On receipt, samples will be logged and stored in an approximately -80°C freezer prior to processing. Analysis will be performed using validated analytical methods in compliance with local standard operating procedures.

Unused samples will be stored for use in future approved research.

17 Statistics

17.1 Statistical methods

A statistical analysis plan will be prepared before the final database lock and will be approved by the TSC.

For peer review only - http://bmjopen.bmj.com/site/about/guidelines.xhtml

17.1.1 Primary analysis

We will report summary statistics on the primary and secondary endpoints according to treatment group. The primary analysis will consist of an estimate, 95% confidence interval and p-value of the absolute risk difference of the incidence rates of the need for medical or surgical rescue therapy within 10 days following the first administration of IV corticosteroids (primary endpoint) between the two treatment arms, using logistic regression to adjust for important baseline covariates. The primary efficacy analysis will be based upon upon the Full Analysis Population (Section 17.1.3).

The baseline characteristics will include:

- Prior diagnosis of IBD at the point of hospitalisation ('first presentation')
- Prior hospitalisation for ASUC
- Previous or current therapy with any of: immunomodulators (azathioprine, 6-mercaptopurine, 6-thioguanine, methotrexate, ciclosporin) biologics (anti-TNF-ɑ monoclonal antibodies, anti-adhesion molecule antibodies, other anti-cytokine antibodies) or oral janus kinase inhibitors
-
- Current or previous oral corticosteroid prescription(s) within 8 weeks prior to first dose of intravenous corticosteroids
- Demographics

Secondary endpoints will be compared in a similar regression model to estimate the treatment effect on an appropriate scale of comparison. This will include an assessment of the effects of treatment with anakinra on the endpoints described in Section 9.6.2.

All continuous variables will be summarised using the following descriptive statistics: n (non-missing sample size), mean, median, maximum, minimum and standard deviation. The frequency and percentages (based on the non-missing sample size) of observed levels will be reported. All summary tables will be structured with a column for each treatment in the order (Control, Anakinra) and will be annotated with the total population size relevant to that table/treatment, including any missing observations.

17.1.2 Secondary analyses

The following secondary analyses will be performed in the trial:

- Analysis of the Randomised Population (Section 17.1.3)
- Complier Average Causal Effect analysis to assess the influence of the amount of treatment received, as distinct from treatment assigned

To test the hypothesis that the clinical effects of anakinra in ASUC may differ between groups of patients with differing levels of prior inflammatory burden, pre-specified subgroup analyses, in the form of estimating treatment-covariate interactions, will be performed on the following baseline characteristics:

- Prior diagnosis of IBD at the point of hospitalisation ('first presentation')
- Prior hospitalisation for ASUC
- Naïvety to any of: immunomodulators (azathioprine, 6-mercaptopurine, 6-thioguanine, methotrexate, ciclosporin) biologics (anti-TNF-ɑ monoclonal antibodies, anti-adhesion molecule antibodies, other anti-cytokine antibodies) or oral janus kinase inhibitorsat the point of hospitalisation.
- Receipt of oral corticosteroids within 8 weeks prior to first dose of intravenous corticosteroids
- Cases of suspected or confirmed ASUC without evidence of CMV reactivation requiring treatment with anti-viral agents and without evidence of a significant GI pathogen
- Duration between 1st dose of IV corticosteroids to 1st dose of IMP.

For peer review only - http://bmjopen.bmj.com/site/about/guidelines.xhtml

17.1.3 Definition of populations

Populations to be analysed in the trial are as follows:

- Full Analysis Population: All randomised participants who receive at least one dose of IMP
- Randomised Population: All randomised participants, regardless of whether IMP was received
- Safety Population: All consenting participants

Any further populations may additionally be defined within the Statistical Analysis Plan

17.2 Interim analyses and criteria for the premature termination of the trial

Except in the case of a valid medical or safety reason, unblinding will only be performed as part of an interim analysis by a designated unblinded statistician. The unblinded statistican will be named in the IDMC charter and will provide outcome data to the iDMC. A blinded report from the iDMC will be made to the TSC and outcome data will not be shared at this stage. Final unblinding of the data to the trial statisticians and TSC will only be made after creation of a locked final analysis data set at the end of the trial.

As part of the interim analyses, the iDMC will examine the unblinded data and provide a recommendation to the TSC as to whether the trial should continue at the following two prespecified points, in addition to other timepoints detailed in the iDMC charter.

- After the feasibility stage (once the first 20 participants have completed the initial 10 days of follow up to report the primary endpoint). Assessment of feasibility will be according to the criteria set out in Section 17.2.1.
- After the first 100 participants have been recruited. Assessment for harm or futility will be according to the criteria set out in Section 17.2.2.

17.2.1 Feasibility study

After the first 20 participants have been recruited, and all completed a minimum of 10 days follow up (to the time of the primary endpoint), feasibility will be assessed. The trial will be regarded as feasible only if all of the following measures are met:

1) ≥10% of eligible participants are recruited (based upon use of screening logs to determine the percentage of eligible participants that are randomised in each site)
2) Adherence to dosing as per protocol (≥80% of participants received appropriately prescribed doses within any given 24h window)
3) Rescue therapy started on or before the 7th day following initial IV corticosteroid administration in ≥80% cases where required during the index admission (as determined by review of timing of rescue therapy administration relative to corticosteroid administration by trial investigators)
4) Maintenance of blinding: there should be no evidence of unnecessary unblinding of participants or investigators.
5) No serious safety concerns identified, especially with regard to infections or SUSARs.

17.2.2 Interim analysis

After the first 100 participants have completed Day 10 assessments, futility analysis will be performed to test the hypothesis that the reduction in absolute risk difference in the primary outcome is ≥10% using a 1-sided 2.5% significance test using logistic regression on the absolute risk reduction scale. If the hypothesis is rejected, i.e. the lower limit of a 95% 2-sided confidence interval for Treatment - Control is above -10%, then the iDMC will consider the recommendation for the study to stop early for futility. This is equivalent to spending 0.1% from the 15% total beta value set to control the

For peer review only - http://bmjopen.bmj.com/site/about/guidelines.xhtml

type 2 error under the alternative hypothesis (49% and 29% rates). If the study continues past the interim, then the final analysis will not be adjusted for the futility analysis, and standard p-values and confidence intervals will be reported.

The formal hypothesis test at the interim is non-binding and will be at the discretion of the iDMC to incorporate into the recommendation they reach. The iDMC will also review safety data, particularly relating to rescue therapy and colectomy rates and infection-related SAEs and advise the TSC accordingly.

The probability of the three possible outcomes (futile, late negative, late positive) from the interim and final analyses is shown in Figure 2, based on the true rates in the arms of the study.

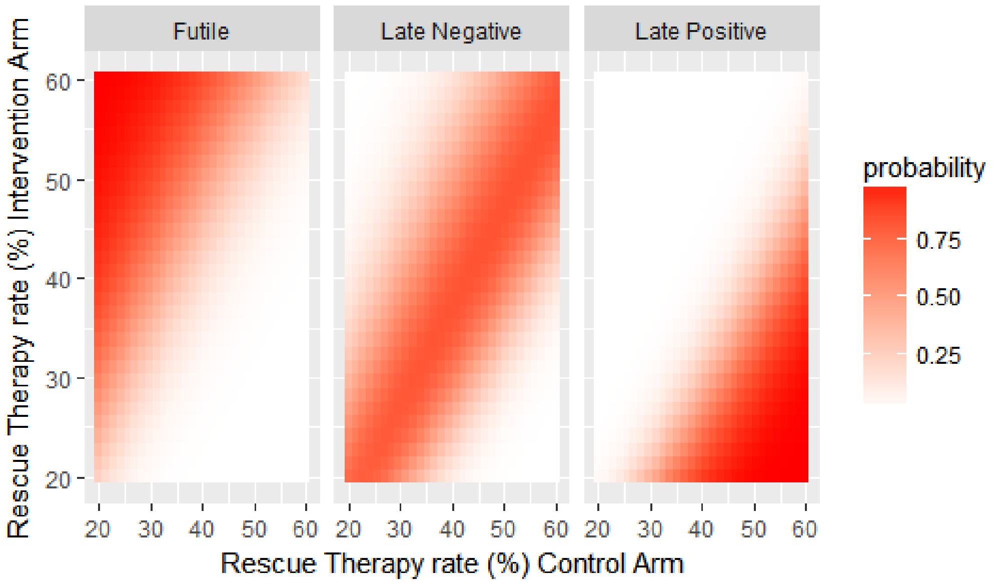

Figure 2. Probability of IASO trial outcomes

17.3 Number of Participants to be enrolled

17.3.1 Sample size calculation

We will recruit 214 patients (107 per group. This will give 85% power to detect a 20% absolute risk reduction (ARR) in the primary endpoint from a rate in the control group of 49% testing at the 5% significance level. Since the main trial observation is brief and performed in an inpatient setting, we expect negligible loss-to-follow up. The trial intervention is well tolerated and we expect low dropout rates.

We have based our estimate of the primary endpoint rate on data from the recently completed NIHR-funded CONSTRUCT trial[31]. This trial recruited 270 patients with ASUC across 52 UK sites between 2010-2013. In this trial, 49% of patients admitted with ASUC needed medical therapy (Prof J Williams, personal communication). Since there are no existing estimates of effect size for anakinra in ASUC, we chose to power IASO to detect a 20% ARR following protocol review by members of the British Society of Gastroenterology IBD clinical research group, who advised that this was the minimum effect size needed that would make a substantial difference to the patient population, and which would be broadly in line with effect sizes seen in other drugs for moderate/severe colitis[32].

For peer review only - http://bmjopen.bmj.com/site/about/guidelines.xhtml

The scientific sub-study based on testing cytokines will be performed on 40 participants. The choice of sample size is based on power studies in transcriptomic studies in UC[33] with adjustment for multiple testing, as well as pragmatic grounds of cost.

17.4 Procedure to account for missing or spurious data

Due to short and intensive period of follow up for the primary endpoint, we do not anticipate significant missing data. For the exploratory analyses of quality of life, which will require postal questionnaire responses some months after discharge, where we are unable to obtain missing data within 12 weeks of the 3- or 6-month timepoints, we will employ the standard approaches to management of missing quality of life data previously described in the CONSTRUCT trial[31].

For participants who withdraw consent to treatment, permission to continue to acquire data for outcome analysis will be sought. For those who do not consent to ongoing monitoring, including those who wish to withdraw entirely from the study, existing data acquired to the point of withdrawal will be included in the final study analysis, with missing data handled according to standard missing data methods, including missing-at-random (MAR) methods with sensitivity testing for deviation from MAR assumptions.

17.5 Definition of the end of the trial

For regulatory notification purposes, the end of trial will be the earliest of:

- The receipt of the final, returned 6 month quality of life questionnaires
- 6 months (+12 weeks) after the last participant entered the study (date of questionnaire censoring).

However, participants may enter a period of long-term follow-up via HES data examination and the trial would remain open to the Ethics Committee until the last participant's HES data capture has been completed.

18 Data handling and record keeping

18.1 CRF

All data will be transferred into a Case Report Form (CRF) which will be anonymised. All trial data in the CRF must be extracted from and be consistent with the relevant source documents. The CRFs must be completed, dated and signed by the investigator or designee in a timely manner. It remains the responsibility of the investigator for the timing, completeness, legibility and accuracy of the CRF pages. The CRF will be accessible to trial coordinators, data managers, the investigators, Clinical Trial Monitors, Auditors and Inspectors as required.

Completed originals of the CRFs should be posted, emailed or faxed to the trial coordination centre within within the timelines detailed in the CRF-completion guidelines.

The investigator will retain a copy of each completed CRF page at site. If faxed or emailed, they will retain the original. The investigator will also supply the trial coordination centre with any required, anonymised background information from the medical records as required.

The investigators must ensure that the CRFs and other trial-related documentation are sent to the trial coordination centre containing no participant identifiable data.

For peer review only - http://bmjopen.bmj.com/site/about/guidelines.xhtml

A separate form containing participant identifiable data necessary for the postage of quality of life questionnaires (name and address) and the capturing of HES data (NHS number and date of birth) will be sent to the central research team. Information contained within this form will be added to a secure database and the physical form kept in a limited-access, locked secure cabinet. Personal identifiable data kept for these purposes will be destroyed at the end of the trial.

All CRF pages must be clear, legible and completed in black ink. Any errors should be crossed with a single stroke so that the original entry can still be seen. Corrections should be inserted and the change dated and initialled by the investigator or designee. If it is not clear why the change has been made, an explanation should be written next to the change. Typing correction fluid must not be used.

18.2 Source Data

To enable peer review, monitoring, audit and/or inspection the investigator must agree to keep records of all participating participants (sufficient information to link records e.g., CRFs, hospital records and samples), all original signed informed consent forms and copies of the CRF pages.

In this trial source data include, but are not limited to:

- Patient medical records,
- Informed Consent Forms,
- Approach and Screening Logs,
- Results of clinical tests
- Questionnaires
- Worksheets for sample collection, processing, storage and shipment.

18.3 Data Protection & Participant Confidentiality

All investigators and trial site staff involved in this trial must comply with the requirements of the Data Protection Act 1998 and Trust Policy with regards to the collection, storage, processing, transfer and disclosure of personal information and will uphold the Act's core principles.

19 Data Monitoring Committee/Trial Steering Committee

Overall supervision of the trial will rest with the TSC. The TSC will be constituted in accordance with EME guidance. In particular, 66% of the TSC members will be required in order to achieve quorum; 75% of members voting must be independent; the TSC chair will be independent. A patient representative will serve as a member of the TSC. The TSC will meet in person or by teleconference with a minimum frequency of 1/year (more frequent meetings may be required at the discretion of the TSC chair) as fully detailed in the TSC Charter.

The TSC will consider recommendations from a fully independent iDMC. iDMC members and their host institutions will not be involved in the running of the trial. Meetings and running of the iDMC will be subject to a separate iDMC charter to be agreed by the iDMC chair.

The TMG will comprise investigators and individuals closely involved in the running of the trial. The TMG will meet more frequently to ensure that all practical details of the trial are progressing well.

During the trial, interim analyses of all SAEs will be provided regularly in strict confidence to the chair of the iDMC in accordance with the charter. In the light of these analyses and any other information considered relevant, the iDMC will advise the TSC

For peer review only - http://bmjopen.bmj.com/site/about/guidelines.xhtml

if, in their view, the randomised comparisons in the trial have provided both (i) "proof beyond reasonable doubt"[1] that for all, or some specific types of patients, use of anakinra is clearly indicated or clearly contraindicated; and (ii) evidence that might reasonably be expected to influence materially the patient management of many clinicians who are already aware of the results of other trials. The TSC will then decide whether to modify the trial, or to seek additional data. Unless this happens, the TSC, collaborators, trial participants and all trial staff (except those who provide the confidential analyses to the iDMC) will remain blind to the interim results on mortality and morbidity until the end of the trial.

20 Ethical & Regulatory considerations

Consent to participate in the scientific sub-study will be sought separately. Patients will be free to elect to participate in the main trial but decline the sub-study.

20.1 Ethical committee review

Before the start of the trial or implementation of any amendment we will obtain approval of the trial protocol, protocol amendments, informed consent forms and other relevant documents (e.g. GP information letters) from the REC and the HRA. All correspondence with the REC/HRA will be retained in the Trial Master File/Investigator Site File.

Annual reports will be submitted to the REC in accordance with national requirements. It is the Chief Investigator's responsibility to produce the annual reports as required.

20.2 Regulatory Compliance

The trial will not commence until a Clinical Trial Authorisation (CTA) is obtained from the MHRA. The protocol and trial conduct will comply with the Medicines for Human Use (Clinical Trials) Regulations 2004 and any relevant amendments.

Development Safety Update Reports (DSURs) will be submitted to the MHRA in accordance with national requirements. It is the Chief Investigators responsibility to produce the annual reports as required.

20.3 Protocol Amendments

Protocol amendments must be reviewed and agreement received from the Sponsor for all proposed amendments prior to submission to the HRA, REC and/or MHRA.

The only circumstance in which an amendment may be initiated prior to HRA, REC and/or MHRA approval is where the change is necessary to eliminate apparent, immediate risks to the participants (Urgent Safety Measures). In this case, accrual of new participants will be halted until the HRA, REC and/or MHRA approval has been obtained. Participating sites will be notified by email or telephone in the event of Urgent Safety Measures.

20.4 Peer Review

The proposed trial has been favourably reviewed by members of the British Society of Gastroenterology IBD Clinical Research Group.

1 Appropriate criteria of proof beyond reasonable doubt cannot be specified precisely, but in general a difference of at least three standard deviations in major morbidity or mortality in an interim analysis would be needed to justify halting, or modifying, the study prematurely. This criterion has the practical advantage that the exact number of interim analyses is of little importance.

For peer review only - http://bmjopen.bmj.com/site/about/guidelines.xhtml

20.5 Public and Patient Involvement

Patient involvement has been actively sought during the planning and preparation stage for this trial proposal, and will form a key part of the trial as it progresses. This is viewed as particularly important given the vital need to safeguard trial recruitment through an acceptable trial design, as well as through the provision of materials that will assist in timely recruitment under difficult conditions for the patients involved.

In December 2014, we started discussions about IASO with members of Crohn's & Colitis UK (CCUK). We invited a wide range of service users to a patient information evening, which we ran as a workshop around trial design. We subsequently established a 10-member patient steering group that has reviewed trial protocols and assisted with responses to the initial application reviews. In particular, this group have provided valuable insight into the acceptability of different dosing regimens and into ways to support patients during the consent process that has informed trial design.

The patient steering group helped to develop the trial protocol, as well as all patient-facing materials that will be used in the trial. A patient representative will serve as a member of the TSC. The patient steering group includes CCUK representatives, and will assist in the preparation and dissemination of plain language summaries of trial updates.

20.6 Declaration of Helsinki and Good Clinical Practice

The trial will be performed in accordance with the spirit and the letter of the declaration of Helsinki, the conditions and principles of Good Clinical Practice, the protocol and applicable local regulatory requirements and laws.

20.7 GCP Training

All trial staff must hold evidence of appropriate GCP training or undergo GCP training prior to undertaking any responsibilities on this trial. This training should be updated every 2 years or in accordance with your Trust's policy.

21 Sponsorship, Financial and Insurance

The trial is sponsored by Cambridge University Hospitals NHS Foundation Trust and University of Cambridge. The trial is funded as described in Table 1.

Table 1. Details of the funders of the IASO trial:

FUNDER(S)	FINANCIAL AND NON FINANCIAL SUPPORT GIVEN
EME programme (NIHR & MRC)	Financial support for all aspects of study
Swedish Orphan Biovitrum	Provision of study drug
Wellcome Trust Sanger Institute	Financial support and academic collaboration for sequencing work

Cambridge University Hospitals NHS Foundation Trust, as a member of the NHS Clinical Negligence Scheme for Trusts, will accept full financial liability for harm caused to participants in the clinical trial caused through the negligence of its employees and honorary contract holders. There are no specific arrangements for compensation should a participant be harmed through participation in the trial, but no-one has acted negligently.

The University of Cambridge will arrange insurance for negligent harm caused as a result of protocol design and for non-negligent harm arising through participation in the clinical trial.

For peer review only - http://bmjopen.bmj.com/site/about/guidelines.xhtml

22 Monitoring, Audit & Inspection

The investigator must make all trial documentation and related records available should an MHRA Inspection occur. Should a monitoring visit or audit be requested, the investigator must make the trial documentation and source data available to the Sponsor's representative. All participant data must be handled and treated confidentially.

The Sponsor's monitoring frequency will be determined by an initial risk assessment performed prior to the start of the trial. A detailed monitoring plan will be generated detailing the frequency and scope of the monitoring for the trial. Throughout the course of the trial, the risk assessment will be reviewed and the monitoring frequency adjusted as necessary.

Remote monitoring will be conducted for all participating sites. The scope and frequency of the monitoring will be determined by the risk assessment and detailed in the Monitoring Plan for the trial.

23 Protocol Compliance and Breaches of GCP

Prospective, planned deviations or waivers to the protocol are not allowed under the UK regulations on Clinical Trials and must not be used. Substantial protocol amendments would be sought from the MHRA and REC in the event of planned changes to eligibility criteria or restrictions.

Protocol deviations, non-compliances, or breaches are departures from the approved protocol. They can happen at any time, but are not planned. They must be adequately documented on the relevant forms and reported to the Chief Investigator and Sponsor immediately.

Deviations from the protocol which are found to occur constantly again and again will not be accepted and will require immediate action and could potentially be classified as a serious breach.

Any potential/suspected serious breaches of GCP must be reported immediately to the Sponsor without any delay.

24 Publications policy

Ownership of the data arising from this trial resides with the trial team. On completion of the trial the data will be analysed and tabulated and a Final Trial Report prepared.

Participating investigators do not have rights to publish any trial data.

This is a NIHR funded trial and the NIHR publishing guidelines will be followed.

Participants will be provided with a link to the study website in the participant information sheet. The website will publish a lay summary of the trial findings.

The NIHR and the Department of Health may publicise the outcome of NIHR-funded research studies through its website, in publications and in press releases where appropriate.

Authorship of final study outputs will be assigned in accordance with guidelines set out by the International Committee of Medical Journal Editors.

For peer review only - http://bmjopen.bmj.com/site/about/guidelines.xhtml

25 Appendices

25.1 Appendix 1 – Authorisation of Participating Sites

25.1.1 Required Documentation

Prior to initiating a participating site, the following documentation is required;

- PI and other key trial team staff CV (signed and dated) and GCP certificate
- Competent Authority approval (HRA, REC, MHRA)
- Local R & D capability and capacity approval
- Participating Site Agreement executed, including pharmacy participating site agreement
- Patient Information Sheets and consent forms on local headed paper
- Protocol signed and dated by PI
- Delegation of Authority Log
- Confirmation of randomisation system training

25.1.2 Procedure for initiating/opening a new site

When all the regulatory paperwork is in place, prior to site opening, an initiation meeting will take place, either face-to-face or via a teleconference. This will be led by the clinical trial coordinator or delegate with as many of the local team present as is practicable. This initiation meeting constitutes training for the trial and it is therefore imperative that all members of the trial team who will be involved in the trial are represented at the meeting. A log of attendees will be completed during the meeting. The presentation slides will be provided to the site in advance of the meeting. A trial initiation form will be completed for each site initiation meeting. Copies of all initiation documentation must be retained in the Investigator Site File (ISF) and TMF.

The sponsor's regulatory green light procedure will be followed. Following the green light, the initial supply of IMP will be ordered for shipment to the site on the authorisation of the coordinating centre coordinator. Following confirmation of receipt of the IMP at site, the site will be opened for recruitment and the randomisation system opened to that site.

25.1.3 Principal Investigator Responsibilities

The PI has overall responsibility for the conduct of the trial at the participating site.

In particular, the PI has responsibilities which include (but are not limited to):

- Ensuring the appropriate approvals are sought and obtained
- Continuing oversight of the trial
- Ensuring the trial is conducted according to the protocol
- Ensuring consent is received in accordance with the protocol and national requirements
- Ensuring that the ISF is accurately maintained
- Delegation of activities to appropriately trained staff (this must be documented on the Delegation of Authority Log)
- Providing protocol or specialised training to new members of the trial team and ensuring that if tasks are delegated, the member of staff is appropriately trained and qualified
- Appropriate attendance at the initiation meeting
- Ensuring appropriate attendance at the TSC/IDMC meetings if required and ensuring appropriate safety information is made available to the coordinating centre team in advance of the meetings

For peer review only - http://bmjopen.bmj.com/site/about/guidelines.xhtml

- Dissemination of important safety or trial-related information to all stakeholders at the participating site
- Safety reporting within the timelines and assessment of causality and expectedness of all SAEs

For peer review only

For peer review only - http://bmjopen.bmj.com/site/about/guidelines.xhtml

25.2 Appendix 2 – Safety Reporting Flow Chart

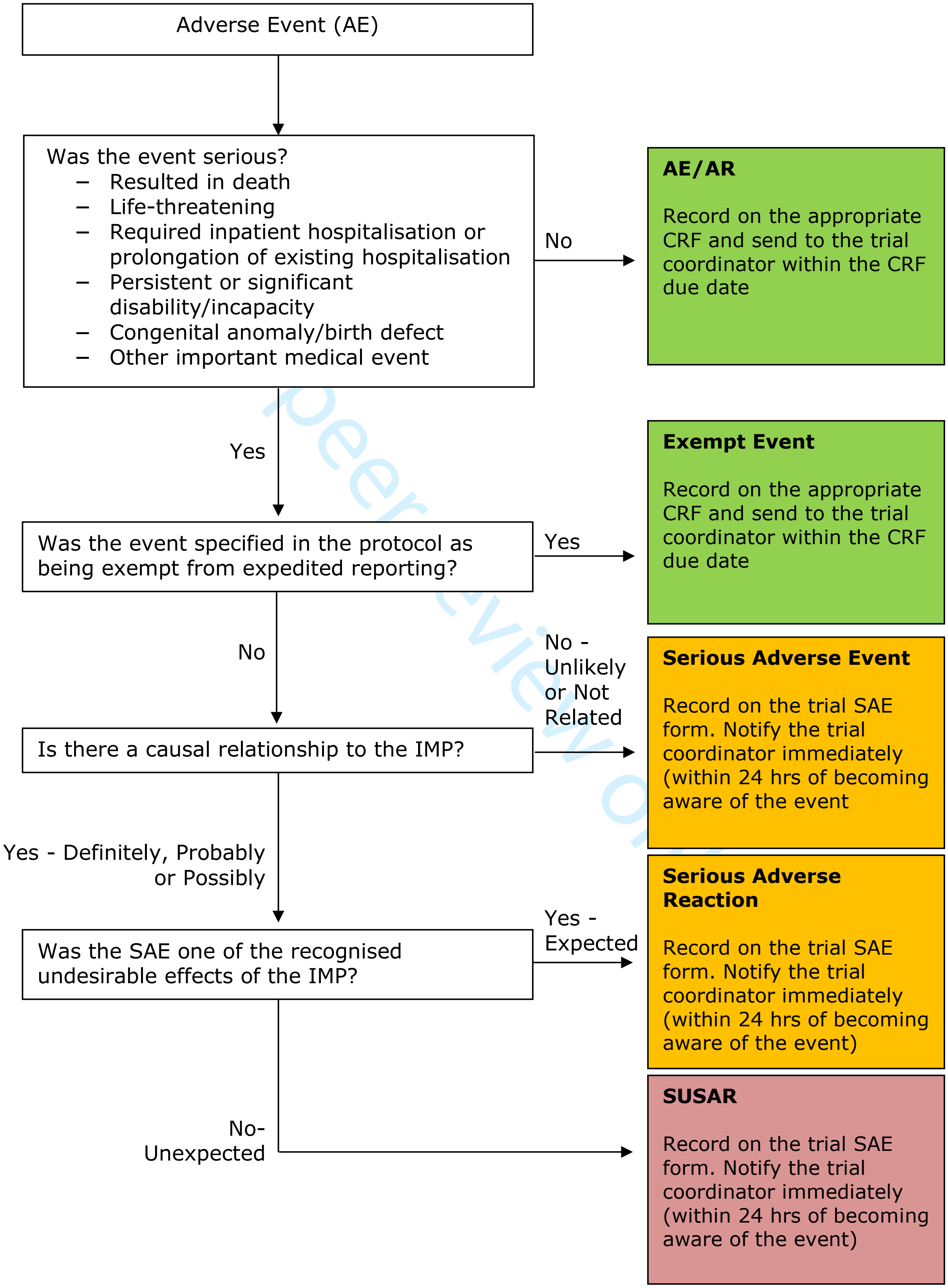

For peer review only - http://bmjopen.bmj.com/site/about/guidelines.xhtml

25.3 Appendix 3 – Amendment History

See page 2

25.4 Appendix 4 – EQ-5D and CUCQ-32 Questionnaires

EQ-5D

Under each heading, please tick the ONE box that best describes your health TODAY.

MOBILITY

I have no problems in walking about ❑
I have slight problems in walking about ❑
I have moderate problems in walking about ❑
I have severe problems in walking about ❑
I am unable to walk about ❑

SELF-CARE

I have no problems washing or dressing myself ❑
I have slight problems washing or dressing myself ❑
I have moderate problems washing or dressing myself ❑
I have severe problems washing or dressing myself ❑
I am unable to wash or dress myself ❑

USUAL ACTIVITIES *(e.g. work, study, housework, family or leisure activities)*

I have no problems doing my usual activities ❑
I have slight problems doing my usual activities ❑
I have moderate problems doing my usual activities ❑
I have severe problems doing my usual activities ❑
I am unable to do my usual activities ❑

PAIN / DISCOMFORT

I have no pain or discomfort ❑
I have slight pain or discomfort ❑
I have moderate pain or discomfort ❑
I have severe pain or discomfort ❑
I have extreme pain or discomfort ❑

ANXIETY / DEPRESSION

I am not anxious or depressed ❑
I am slightly anxious or depressed ❑
I am moderately anxious or depressed ❑
I am severely anxious or depressed ❑
I am extremely anxious or depressed ❑

For peer review only - http://bmjopen.bmj.com/site/about/guidelines.xhtml

™

- We would like to know how good or bad your health is TODAY.
- This scale is numbered from 0 to 100.
- 100 means the best health you can imagine.
  0 means the worst health you can imagine.
- Mark an X on the scale to indicate how your health is TODAY.
- Now, please write the number you marked on the scale in the box below.

YOUR HEALTH TODAY = [ ]

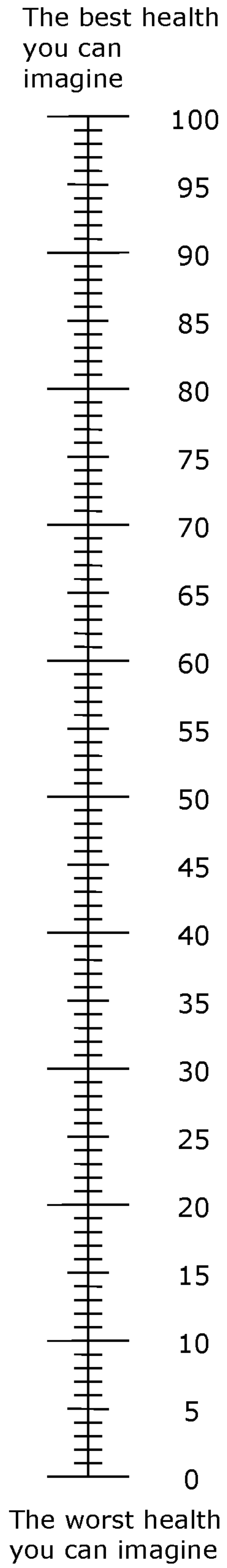

For peer review only - http://bmjopen.bmj.com/site/about/guidelines.xhtml

CUCQ-32

The following questions ask for your views about your bowel problem and how it has affected your life over the **last two weeks**. Please answer all the questions. If you are unsure about how to answer any question, just give the best answer you can. Do not spend too much time answering, as your first thoughts are likely to be the most accurate. If you do not wish to answer any of these questions, please leave it blank and complete the details of the question and reason(s) why it was not answered.

1. On how many days over the last two weeks have you had loose or runny bowel movements? …………………… days

2. On how many days in the last two weeks have you noticed blood in your stools?
…………………… days

3. On how many days over the last two weeks have you felt tired?
……………………. days

4. In the last two weeks have you felt frustrated?
a) No, not at all
b) Yes, some of the time
c) Yes, most of the time
d) Yes, all of the time

5. In the last two weeks, has your bowel condition prevented you from carrying out your work or other normal activities?
a) No, not at all
b) Yes, some of the time
c) Yes, most of the time
d) Yes, all of the time

6. On how many days over the last two weeks have you opened your bowels more than three times a day?
………………… days

7. On how many days over the last two weeks have you felt full of energy?
……………….. days

8. In the last two weeks did your bowel condition prevent you from going out socially?
a) No, not at all
b) Yes, some of the time
c) Yes, most of the time
d) Yes, all of the time

9. On how many days over the last two weeks have your bowels opened accidentally? …………………….. days

10. On how many days over the last two weeks have you felt generally unwell?
……………. days

11. In the last two weeks have you felt the need to keep close to a toilet?
a) No, not at all

For peer review only - http://bmjopen.bmj.com/site/about/guidelines.xhtml

b) Yes, some of the time
c) Yes, most of the time
d) Yes, all of the time

12. In the last two weeks, has your bowel condition affected your leisure or sports activities?
a) No, not at all
b) Yes, some of the time
c) Yes, most of the time
d) Yes, all of the time

13. On how many days over the last two weeks have you felt pain in your abdomen?
……….. days

14. On how many nights over the last two weeks have you been unable to sleep well (days if you are a shift worker)?
………………… nights (or days)

15. On how many nights in the last two weeks have you had to get up to use the toilet because of your bowel condition after you have gone to bed?
………… nights

16. In the last two weeks have you felt depressed?
a) No, not at all
b) Yes, some of the time
c) Yes, most of the time
d) Yes, all of the time

17. In the last two weeks have you had to avoid attending events where there was no toilet close at hand?
a) No, not at all
b) Yes, some of the time
c) Yes, most of the time
d) Yes, all of the time

18. On how many days over the last two weeks, have you had a problem with large amounts of wind?
………………….. days

19. On how many days over the last two weeks have you felt off your food?
…………….. days

20. Many patients with bowel problems have worries about their illness. How often during the last two weeks have you felt worried?
a) No, not at all
b) Yes, some of the time
c) Yes, most of the time
d) Yes, all of the time

21. On how many days over the last two weeks has your abdomen felt bloated?
……………… days

22. In the last two weeks have you felt relaxed?

For peer review only - http://bmjopen.bmj.com/site/about/guidelines.xhtml
For peer review only

a) No, not at all
b) Yes, some of the time
c) Yes, most of the time
d) Yes, all of the time

23. In the last two weeks have you been embarrassed by your bowel problem?
a) No, not at all
b) Yes, some of the time
c) Yes, most of the time
d) Yes, all of the time

24. On how many days over the last two weeks have you wanted to go back to the toilet immediately after you thought you had emptied your bowels?
………………… days

25. In the last two weeks have you felt upset?
a) No, not at all
b) Yes, some of the time
c) Yes, most of the time
d) Yes, all of the time

26. On how many days over the last two weeks have you had to rush to the toilet?
…………. days

27. In the last two weeks have you felt angry as a result of your bowel problem?
a) No, not at all
b) Yes, some of the time
c) Yes, most of the time
d) Yes, all of the time

28. In the last two weeks, has your sex life been affected by your bowel problem?
a) No, not at all
b) Yes, some of the time
c) Yes, most of the time
d) Yes, all of the time

29. On how many days over the last two weeks have you felt sick?
………….. days

30. In the last two weeks have you felt irritable?
a) No, not at all
b) Yes, some of the time
c) Yes, most of the time
d) Yes, all of the time

31. In the last two weeks have you felt lack of sympathy from others?
a) No, not at all
b) Yes, some of the time
c) Yes, most of the time
d) Yes, all of the time

32. In the last two weeks have you felt happy?
a) No, not at all

For peer review only - http://bmjopen.bmj.com/site/about/guidelines.xhtml
For peer review only

b) Yes, some of the time
c) Yes, most of the time
d) Yes, all of the time

If you did not complete any of these questions, please record the question number(s) below and, if possible, give a reason why it was not completed.

For peer review only - http://bmjopen.bmj.com/site/about/guidelines.xhtml

25.5 Appendix 4 – Treatment decision tree for IASO trial

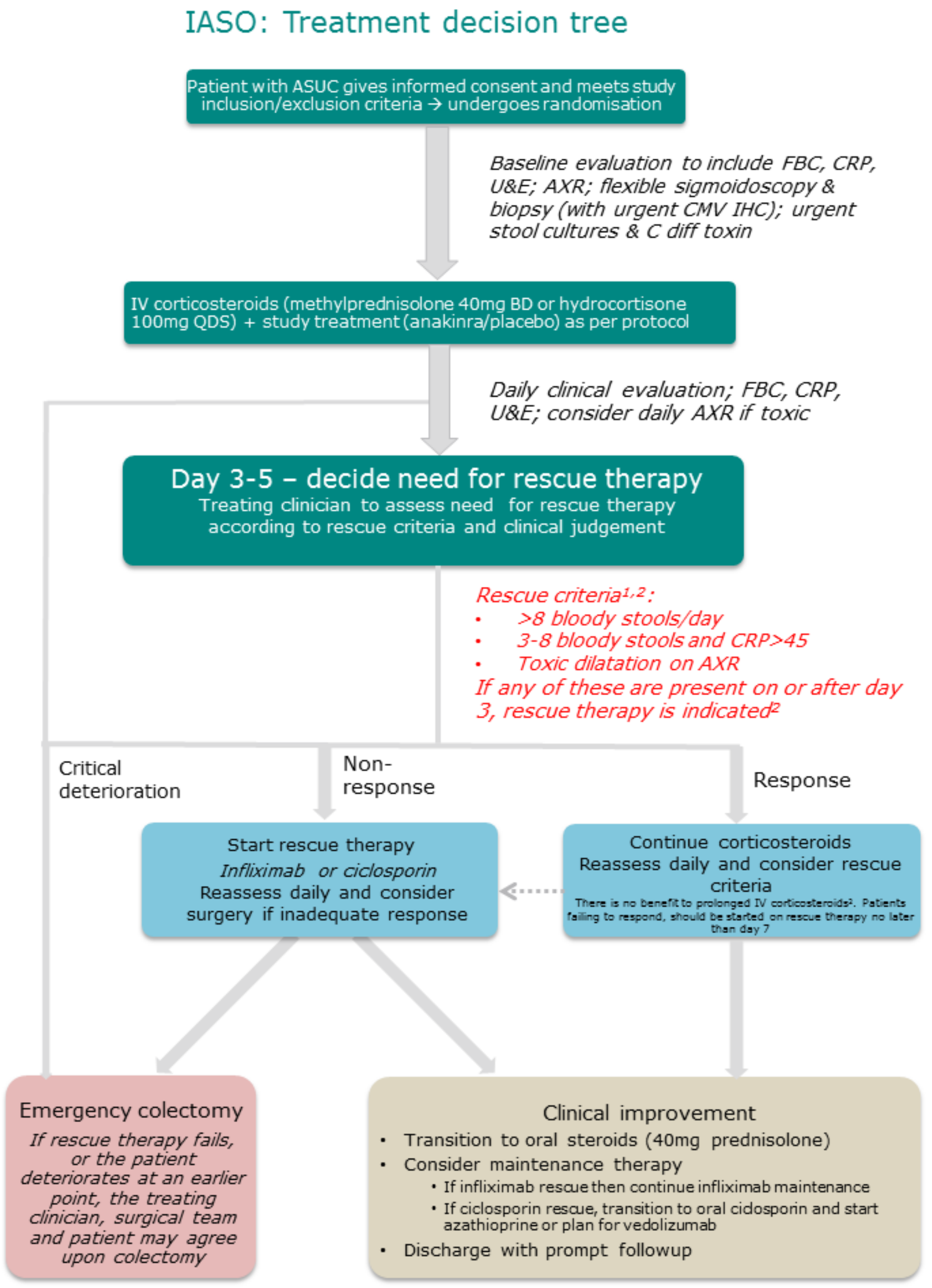

1. Travis SL *et al.* *Gut* 1996;**38**:905-910
2. Harbord M *et al.* *J Crohn's Colitis* 2017 **DOI:** https://doi.org/10.1093/ecco-jcc/jjx009

For peer review only - http://bmjopen.bmj.com/site/about/guidelines.xhtml

26 REFERENCES

1. NELA_project_team, *First patient report of the National Emergency Laparotomy Audit*. 2015, London: RCoA London.
2. Dignass, A., et al., *Second European evidence-based consensus on the diagnosis and management of ulcerative colitis part 1: definitions and diagnosis.* J Crohns Colitis, 2012. **6**(10): p. 965-90.
3. Dinarello, C.A., *Interleukin-1 in the pathogenesis and treatment of inflammatory diseases.* Blood, 2011. **117**(14): p. 3720-32.
4. Planell, N., et al., *Transcriptional analysis of the intestinal mucosa of patients with ulcerative colitis in remission reveals lasting epithelial cell alterations.* Gut, 2013. **62**(7): p. 967-76.
5. Hendel, J., et al., *A simple filter-paper technique allows detection of mucosal cytokine levels in vivo in ulcerative colitis. Interleukin-1 and interleukin-1-receptor antagonist.* Dig Dis Sci, 1996. **41**(9): p. 1775-9.
6. Ludwiczek, O., et al., *Imbalance between interleukin-1 agonists and antagonists: relationship to severity of inflammatory bowel disease.* Clin Exp Immunol, 2004. **138**(2): p. 323-9.
7. Dinarello, C.A., A. Simon, and J.W. van der Meer, *Treating inflammation by blocking interleukin-1 in a broad spectrum of diseases.* Nat Rev Drug Discov, 2012. **11**(8): p. 633-52.
8. Cominelli, F. and T.T. Pizarro, *Interleukin-1 and interleukin-1 receptor antagonist in inflammatory bowel disease.* Aliment Pharmacol Ther, 1996. **10 Suppl 2**: p. 49-53; discussion 54.
9. Opal, S.M., et al., *Confirmatory interleukin-1 receptor antagonist trial in severe sepsis: a phase III, randomized, double-blind, placebo-controlled, multicenter trial. The Interleukin-1 Receptor Antagonist Sepsis Investigator Group.* Crit Care Med, 1997. **25**(7): p. 1115-24.
10. Jiang, Y., et al., *A multicenter, double-blind, dose-ranging, randomized, placebo-controlled study of recombinant human interleukin-1 receptor antagonist in patients with rheumatoid arthritis: radiologic progression and correlation of Genant and Larsen scores.* Arthritis Rheum, 2000. **43**(5): p. 1001-9.
11. de Luca, A., et al., *IL-1 receptor blockade restores autophagy and reduces inflammation in chronic granulomatous disease in mice and in humans.* Proc Natl Acad Sci U S A, 2014. **111**(9): p. 3526-31.
12. Bersudsky, M., et al., *Non-redundant properties of IL-1alpha and IL-1beta during acute colon inflammation in mice.* Gut, 2014. **63**(4): p. 598-609.
13. Yang, B.B., S. Baughman, and J.T. Sullivan, *Pharmacokinetics of anakinra in subjects with different levels of renal function.* Clin Pharmacol Ther, 2003. **74**(1): p. 85-94.
14. Arend, W.P. and B.P. Coll, *Interaction of recombinant monocyte-derived interleukin 1 receptor antagonist with rheumatoid synovial cells.* Cytokine, 1991. **3**(5): p. 407-13.
15. Carty, E., et al., *Measurement of in vivo rectal mucosal cytokine and eicosanoid production in ulcerative colitis using filter paper.* Gut, 2000. **46**(4): p. 487-92.
16. Langereis, J.D., et al., *Steroids induce a disequilibrium of secreted interleukin-1 receptor antagonist and interleukin-1beta synthesis by human neutrophils.* Eur Respir J, 2011. **37**(2): p. 406-15.

For peer review only

For peer review only - http://bmjopen.bmj.com/site/about/guidelines.xhtml

17. Truelove, S.C. and L.J. Witts, *Cortisone in ulcerative colitis; final report on a therapeutic trial.* Br Med J, 1955. **2**(4947): p. 1041-8.
18. Lichtiger, S., et al., *Cyclosporine in severe ulcerative colitis refractory to steroid therapy.* N Engl J Med, 1994. **330**(26): p. 1841-5.
19. Jarnerot, G., et al., *Infliximab as rescue therapy in severe to moderately severe ulcerative colitis: a randomized, placebo-controlled study.* Gastroenterology, 2005. **128**(7): p. 1805-11.
20. Laharie, D., et al., *Ciclosporin versus infliximab in patients with severe ulcerative colitis refractory to intravenous steroids: a parallel, open-label randomised controlled trial.* Lancet, 2012. **380**(9857): p. 1909-15.
21. Irvine, E.J., *Review article: patients' fears and unmet needs in inflammatory bowel disease.* Aliment Pharmacol Ther, 2004. **20 Suppl 4**: p. 54-9.
22. Turner, D., et al., *Response to corticosteroids in severe ulcerative colitis: a systematic review of the literature and a meta-regression.* Clin Gastroenterol Hepatol, 2007. **5**(1): p. 103-10.
23. Galea, J., et al., *Reduction of inflammation after administration of interleukin-1 receptor antagonist following aneurysmal subarachnoid hemorrhage: results of the Subcutaneous Interleukin-1Ra in SAH (SCIL-SAH) study.* J Neurosurg, 2017: p. 1-9.
24. Lee, J.C., et al., *Gene expression profiling of CD8+ T cells predicts prognosis in patients with Crohn disease and ulcerative colitis.* J Clin Invest, 2011. **121**(10): p. 4170-9.
25. EuroQol, G., *EuroQol--a new facility for the measurement of health-related quality of life.* Health Policy, 1990. **16**(3): p. 199-208.
26. Alrubaiy, L., et al., *Development of a short questionnaire to assess the quality of life in Crohn's disease and ulcerative colitis.* J Crohns Colitis, 2015. **9**(1): p. 66-76.
27. Kaiser, C., et al., *Injection-site reactions upon Kineret (anakinra) administration: experiences and explanations.* Rheumatol Int, 2012. **32**(2): p. 295-9.
28. Geboes, K., et al., *A reproducible grading scale for histological assessment of inflammation in ulcerative colitis.* Gut, 2000. **47**(3): p. 404-9.
29. Marchal-Bressenot, A., et al., *Development and validation of the Nancy histological index for UC.* Gut, 2015.
30. Mosli, M.H., et al., *Development and validation of a histological index for UC.* Gut, 2015.
31. Seagrove, A.C., et al., *Randomised controlled trial. Comparison Of iNfliximab and ciclosporin in STeroid Resistant Ulcerative Colitis: Trial design and protocol (CONSTRUCT).* BMJ Open, 2014. **4**(4): p. e005091.
32. Rutgeerts, P., et al., *Infliximab for induction and maintenance therapy for ulcerative colitis.* N Engl J Med, 2005. **353**(23): p. 2462-76.
33. Boland, B.S., et al., *Validated gene expression biomarker analysis for biopsy-based clinical trials in ulcerative colitis.* Aliment Pharmacol Ther, 2014. **40**(5): p. 477-85.

For peer review only - http://bmjopen.bmj.com/site/about/guidelines.xhtml

SPIRIT

STANDARD PROTOCOL ITEMS: RECOMMENDATIONS FOR INTERVENTIONAL TRIALS

SPIRIT 2013 Checklist: Recommended items to address in a clinical trial protocol and related documents*

**Section/item**	**Item No**	**Description**	**Page in Summary Manuscript (and full protocol if specified)**
**Administrative information**			
Title	1	Descriptive title identifying the study design, population, interventions, and, if applicable, trial acronym	p.1
Trial registration	2a	Trial identifier and registry name. If not yet registered, name of intended registry	p.1
	2b	All items from the World Health Organization Trial Registration Data Set	p.1, 4-5, 12-13
Protocol version	3	Date and version identifier	p.2
Funding	4	Sources and types of financial, material, and other support	p.13
Roles and responsibilities	5a	Names, affiliations, and roles of protocol contributors	p.13
	5b	Name and contact information for the trial sponsor	p.13 (contact information in full protocol – Sponsor oversight carried out by Cambridge Clinical Trials Unit)

For peer review only

	5c	Role of study sponsor and funders, if any, in study design; collection, management, analysis, and interpretation of data; writing of the report; and the decision to submit the report for publication, including whether they will have ultimate authority over any of these activities	p. 12-13 (Sponsorship & Funding)<br><br>In full protocol: Sponsor oversight of adverse events (p.35), approval of amendments (p.44), monitoring (p.46).<br><br>Full sponsor review of the trial, including the protocol, was performed prior to submitting for ethics, HRA and MHRA approval.
	5d	Composition, roles, and responsibilities of the coordinating centre, steering committee, endpoint adjudication committee, data management team, and other individuals or groups overseeing the trial, if applicable (see Item 21a for data monitoring committee)	p.11 (roles of the committees)<br>Composition covered in full protocol p.43-44
**Introduction**			
Background and rationale	6a	Description of research question and justification for undertaking the trial, including summary of relevant studies (published and unpublished) examining benefits and harms for each intervention	p.2-3
	6b	Explanation for choice of comparators	p.2-3
Objectives	7	Specific objectives or hypotheses	p.3

For peer review only

Trial design	8	Description of trial design including type of trial (eg, parallel group, crossover, factorial, single group), allocation ratio, and framework (eg, superiority, equivalence, noninferiority, exploratory)	p.4

Methods: Participants, interventions, and outcomes

Study setting	9	Description of study settings (eg, community clinic, academic hospital) and list of countries where data will be collected. Reference to where list of study sites can be obtained	p.4 (information about study site locations not featured in protocol but can be viewed at http://www.isrctn.com/ISRCTN43717130)
Eligibility criteria	10	Inclusion and exclusion criteria for participants. If applicable, eligibility criteria for study centres and individuals who will perform the interventions (eg, surgeons, psychotherapists)	p.4-5
Interventions	11a	Interventions for each group with sufficient detail to allow replication, including how and when they will be administered	p.6 (extensive details covered in full protocol Section 11)
	11b	Criteria for discontinuing or modifying allocated interventions for a given trial participant (eg, drug dose change in response to harms, participant request, or improving/worsening disease)	p.6
	11c	Strategies to improve adherence to intervention protocols, and any procedures for monitoring adherence (eg, drug tablet return, laboratory tests)	p.11
	11d	Relevant concomitant care and interventions that are permitted or prohibited during the trial	p.4-6

Outcomes	12	Primary, secondary, and other outcomes, including the specific measurement variable (eg, systolic blood pressure), analysis metric (eg, change from baseline, final value, time to event), method of aggregation (eg, median, proportion), and time point for each outcome. Explanation of the clinical relevance of chosen efficacy and harm outcomes is strongly recommended	p.5-6 (further information in full protocol p.20-21)
Participant timeline	13	Time schedule of enrolment, interventions (including any run-ins and washouts), assessments, and visits for participants. A schematic diagram is highly recommended (see Figure)	p.4 + Figure 1
Sample size	14	Estimated number of participants needed to achieve study objectives and how it was determined, including clinical and statistical assumptions supporting any sample size calculations	p.10
Recruitment	15	Strategies for achieving adequate participant enrolment to reach target sample size	p.7 (identification of participants)<br>p.11-12 interim analyses demonstrate formal testing procedure for assessing the feasibility of the trial including recruitment

Methods: Assignment of interventions (for controlled trials)

Allocation:

Sequence generation	16a	Method of generating the allocation sequence (eg, computer-generated random numbers), and list of any factors for stratification. To reduce predictability of a random sequence, details of any planned restriction (eg, blocking) should be provided in a separate document that is unavailable to those who enrol participants or assign interventions	p.7 (stratification) Allocation sequence generation method not specified in the protocol – the randomisation and concealment lists for the trial are generated by the provider of the randomisation system (Sealed Envelope)
Allocation concealment mechanism	16b	Mechanism of implementing the allocation sequence (eg, central telephone; sequentially numbered, opaque, sealed envelopes), describing any steps to conceal the sequence until interventions are assigned	p.7
Implementation	16c	Who will generate the allocation sequence, who will enrol participants, and who will assign participants to interventions	p.7 (identification and enrolment). Full protocol (p.22) details who will be able to randomise patients<br><br>Allocation sequence generation method not specified in the protocol – the randomisation and concealment lists for the trial are generated by the provider of the randomisation system (Sealed Envelope)
Blinding (masking)	17a	Who will be blinded after assignment to interventions (eg, trial participants, care providers, outcome assessors, data analysts), and how	p.7 (full details in full protocol Section 10.4, p.22)

	17b	If blinded, circumstances under which unblinding is permissible, and procedure for revealing a participant's allocated intervention during the trial	Full protocol Section 11.4 (p.26)
**Methods: Data collection, management, and analysis**			
Data collection methods	18a	Plans for assessment and collection of outcome, baseline, and other trial data, including any related processes to promote data quality (eg, duplicate measurements, training of assessors) and a description of study instruments (eg, questionnaires, laboratory tests) along with their reliability and validity, if known. Reference to where data collection forms can be found, if not in the protocol	p.7-9<br>p.12 (use of case report forms; location is not specified in protocol)
	18b	Plans to promote participant retention and complete follow-up, including list of any outcome data to be collected for participants who discontinue or deviate from intervention protocols	p.12 (missing data)
Data management	19	Plans for data entry, coding, security, and storage, including any related processes to promote data quality (eg, double data entry; range checks for data values). Reference to where details of data management procedures can be found, if not in the protocol	p.12 (data management)<br>More information available in full protocol Section 18 (p.42-43)

Statistical methods	20a	Statistical methods for analysing primary and secondary outcomes. Reference to where other details of the statistical analysis plan can be found, if not in the protocol	p.10-12<br>More information available in full protocol Section 17 (p.38-41)
	20b	Methods for any additional analyses (eg, subgroup and adjusted analyses)	p.11 (secondary analyses)<br>More information available in full protocol Section 17 (p.38-41)
	20c	Definition of analysis population relating to protocol non-adherence (eg, as randomised analysis), and any statistical methods to handle missing data (eg, multiple imputation)	p.10 (Patient Population)<br>p.12 (Missing Data)
**Methods: Monitoring**			
Data monitoring	21a	Composition of data monitoring committee (DMC); summary of its role and reporting structure; statement of whether it is independent from the sponsor and competing interests; and reference to where further details about its charter can be found, if not in the protocol. Alternatively, an explanation of why a DMC is not needed	p.11<br>Composition covered in full protocol p.43-44
	21b	Description of any interim analyses and stopping guidelines, including who will have access to these interim results and make the final decision to terminate the trial	p.11 (interim analyses)
Harms	22	Plans for collecting, assessing, reporting, and managing solicited and spontaneously reported adverse events and other unintended effects of trial interventions or trial conduct	p.9 (safety monitoring)<br>More information available in full protocol Section 13 (p.33-38)

Auditing	23	Frequency and procedures for auditing trial conduct, if any, and whether the process will be independent from investigators and the sponsor	Information available in full protocol Section 22 (p.46)
**Ethics and dissemination**			
Research ethics approval	24	Plans for seeking research ethics committee/institutional review board (REC/IRB) approval	p.12 (Approvals)
Protocol amendments	25	Plans for communicating important protocol modifications (eg, changes to eligibility criteria, outcomes, analyses) to relevant parties (eg, investigators, REC/IRBs, trial participants, trial registries, journals, regulators)	See full protocol Section 20.3, p.44
Consent or assent	26a	Who will obtain informed consent or assent from potential trial participants or authorised surrogates, and how (see Item 32)	p.7 (Participant Identification & Informed Consent)
	26b	Additional consent provisions for collection and use of participant data and biological specimens in ancillary studies, if applicable	This is covered in the consent form for the trial
Confidentiality	27	How personal information about potential and enrolled participants will be collected, shared, and maintained in order to protect confidentiality before, during, and after the trial	p.12 (data management) More information in full protocol Section 18.1, p.43
Declaration of interests	28	Financial and other competing interests for principal investigators for the overall trial and each study site	p.14

For peer review only

Access to data	29	Statement of who will have access to the final trial dataset, and disclosure of contractual agreements that limit such access for investigators	p.13 (data sharing)
Ancillary and post-trial care	30	Provisions, if any, for ancillary and post-trial care, and for compensation to those who suffer harm from trial participation	See full trial protocol Section 21 (p.45)
Dissemination policy	31a	Plans for investigators and sponsor to communicate trial results to participants, healthcare professionals, the public, and other relevant groups (eg, via publication, reporting in results databases, or other data sharing arrangements), including any publication restrictions	p.13 (dissemination plan)
	31b	Authorship eligibility guidelines and any intended use of professional writers	p.13 (dissemination plan)
	31c	Plans, if any, for granting public access to the full protocol, participant-level dataset, and statistical code	p.13 (dissemination plan)<br>p.13 (data sharing)
**Appendices**			
Informed consent materials	32	Model consent form and other related documentation given to participants and authorised surrogates	Separate document; not included with the trial protocol
Biological specimens	33	Plans for collection, laboratory evaluation, and storage of biological specimens for genetic or molecular analysis in the current trial and for future use in ancillary studies, if applicable	See full trial protocol (Section 16, p.38)

*It is strongly recommended that this checklist be read in conjunction with the SPIRIT 2013 Explanation & Elaboration for important clarification on the items. Amendments to the protocol should be tracked and dated. The SPIRIT checklist is copyrighted by the SPIRIT

For peer review only - http://bmjopen.bmj.com/site/about/guidelines.xhtml

Group under the Creative Commons "Attribution-NonCommercial-NoDerivs 3.0 Unported" license.

For peer review only

BMJ Open

Trial Summary & Protocol for a phase II randomised placebo controlled double blinded trial of Interleukin 1 blockade in Acute Severe Colitis – the IASO Trial

Journal:	*BMJ Open*
Manuscript ID	bmjopen-2018-023765.R1
Article Type:	Protocol
Date Submitted by the Author:	21-Sep-2018
Complete List of Authors:	Thomas, Martin ; Cambridge Clinical Trials Unit<br>Bayliss, Carrie; Cambridge Clinical Trials Unit<br>Bond, Simon; Cambridge Clinical Trials Unit; MRC Biostatistics Unit<br>Dowling, Francis; Cambridge Clinical Trials Unit<br>Galea, James; University of Manchester<br>Jairath, Vipul; Department of Medicine, Division of Gastroenterology, Western University; Division of Epidemiology and Biostatistics, Western University<br>Lamb, Christopher; Institute of Cellular Medicine, Newcastle University; Newcastle Upon Tyne Hospitals NHS Foundation Trust, Department of Gastroenterology<br>Probert, Chris; Institue of Translational Medicine, University of Liverpool<br>Timperley-Preece, Elizabeth<br>Watson, Alastair; University of East Anglia<br>Whitehead, Lynne; Addenbrooke's Hospital<br>Williams, John; Swansea University Medical School<br>Parkes, M; Addenbrooke's Hospital; Department of Medicine, University of Cambridge<br>Kaser, Arthur; Department of Medicine, University of Cambridge<br>Raine, Tim; Addenbrooke's Hospital; Department of Medicine, University of Cambridge
**Primary Subject Heading**:	Gastroenterology and hepatology
Secondary Subject Heading:	Immunology (including allergy)
Keywords:	GASTROENTEROLOGY, Inflammatory bowel disease < GASTROENTEROLOGY, IMMUNOLOGY, Immunology < BASIC SCIENCES

SCHOLARONE™
Manuscripts

Trial Summary & Protocol for a phase II randomised placebo controlled double blinded trial of Interleukin 1 blockade in Acute Severe Colitis – the IASO Trial

Thomas MG (Correspondence: martin.thomas@addenbrookes.nhs.uk), Bayliss C, Bond S, Dowling F, Galea J, Jairath V, Lamb CA, Probert C, Timperley-Preece E, Watson A, Whitehead L, Williams JG, Parkes M, Kaser A, Raine T

Author Affiliations

Thomas, Martin; Cambridge Clinical Trials Unit

Bayliss, Carrie; Cambridge Clinical Trials Unit

Bond, Simon; Cambridge Clinical Trials Unit; MRC Biostatistics Unit

Dowling, Francis; Cambridge Clinical Trials Unit

Galea, James; University of Manchester

Jairath, Vipul; Department of Medicine, Division of Gastroenterology,

Western University; Division of Epidemiology and Biostatistics, Western University

Lamb, Christopher; Institute of Cellular Medicine, Newcastle University;

Newcastle Upon Tyne Hospitals NHS Foundation Trust, Department of Gastroenterology

Probert, Chris; Institute of Translational Medicine, University of Liverpool

Timperley-Preece, Elizabeth

Watson, Alastair; University of East Anglia

Whitehead, Lynne; Addenbrooke's Hospital

Williams, John; Swansea University Medical School

Parkes, Miles; Addenbrooke's Hospital; Department of Medicine, University of Cambridge

Kaser, Arthur; Department of Medicine, University of Cambridge

Raine, Tim; Addenbrooke's Hospital; Department of Medicine, University of Cambridge

Abstract

Introduction: Acute severe ulcerative colitis (ASUC) is a severe manifestation of ulcerative colitis (UC) that warrants hospitalisation. Despite significant advances in therapeutic options for UC and in the medical management of steroid-refractory ASUC, the initial treatment paradigm has not changed since 1955 and is based on the use of intravenous (IV) corticosteroids. This treatment is successful in approximately 50% of patients but failure of this and subsequent medical therapy still occurs, with colectomy rates of up to 40% reported. The Interleukin 1 (IL-1) blockade in Acute Severe Colitis (IASO) trial aims to investigate whether antagonism of IL-1 signalling using anakinra in addition to IV corticosteroid treatment can improve outcomes in patients with ASUC.

Methods and Analysis: IASO is a phase 2, multi-centre, two-arm (parallel group), randomised (1:1), placebo-controlled, double-blinded trial of short-duration anakinra in ASUC. Its primary outcome will be the incidence of medical (e.g. infliximab/ciclosporin) or surgical rescue therapy (colectomy) within 10 days following the commencement of IV corticosteroid therapy. Secondary outcomes will include disease activity, time to clinical response, time to rescue

For peer review only - http://bmjopen.bmj.com/site/about/guidelines.xhtml

therapy, colectomy incidence by day 98 post IV corticosteroids, and safety. The trial aims to recruit 214 patients across 20 sites in the UK.

Ethics and Dissemination: The trial has received approval from the Cambridge Central Research Ethics Committee (Ref. 17/EE/0347), the Health Research Authority (Ref: 201505) and Clinical Trials Authorisation from the Medicines and Healthcare products Regulatory Agency. We plan to present trial findings at scientific conferences and publish in high-impact peer-reviewed journals.

Trial Registrations: ISRCTN: 43717130 (12/10/2017); EudraCT: 2017-001389-10 (30/03/2017)

Strengths and limitations of this study

- A multicentre, UK-wide trial of 214 participants at 20 sites
- The largest first-line treatment study ever conducted in this patient group
- Potential for a step-change and improved outcomes in acutely ill patients, including cost-savings for the NHS
- Pragmatic trial with wide eligibility criteria

For peer review only

For peer review only - http://bmjopen.bmj.com/site/about/guidelines.xhtml

Introduction

Background & Rationale

Ulcerative colitis (UC) is an inflammatory condition of the colon associated with diarrhoea, rectal bleeding and abdominal pain. UC affects around 120,000 people in the UK.[1] UC tends to follow a relapsing/remitting course with periods of comparative health interspersed with unpredictable acute 'flares'. These may be severe enough to mandate hospital admission. For patients with UC in the UK, annual hospitalisation rates with acute severe UC (ASUC) are around 3% (3,600 individuals). 20-40% of these patients will require emergency surgical removal of the colon (colectomy) during the same hospital admission.[2,3] A recent UK audit of emergency surgery in colitis showed mortality rates of 4% at 90 days.[4] Additionally, surgery carries significant morbidity associated with the need for at least a temporary stoma, as well as long-term complications (both physical [e.g. adhesional disease] and psychological) and complications of subsequent procedures (e.g. pelvic nerve damage with ileal-pouch anal anastomosis or completion proctectomy).

Treatment for ASUC involves high dose intravenous (IV) corticosteroids and is standardised worldwide.[5] After 3-5 days, patients who do not exhibit an improvement may be considered for medical 'rescue' therapy with further immunosuppression using either infliximab or ciclosporin. Those patients who subsequently fail to respond to medical treatment, or patients with fulminant colitis at presentation, will then proceed to surgery, typically within the space of 4-10 days of rescue therapy initiation. This is usually performed on the same admission with a subtotal colectomy and creation of an end ileostomy.

The initial management of ASUC has changed little since the seminal trial of IV corticosteroids in 1955.[6] Despite significant advances in therapeutic options and drug development for ambulatory patients with UC, patients with ASUC are typically excluded from the eligibility criteria of standard clinical trials for patients with moderate-severe UC. Clinical trials that have compared infliximab or ciclosporin as medical rescue treatments for ASUC have required patients to fail to respond to treatment with up to 5 days of intravenous steroids.[7–9] In the absence of reliable early prognostic markers, it may be appealing to treat all patients presenting with ASUC with immunosuppressive treatments normally reserved for rescue therapy rather than corticosteroids alone. The barriers for this approach include the toxicity of such treatment [10–13] as well as the financial burden incurred.[14] There is still therefore an unmet need to improve the management of ASUC by doing something at the point of initial treatment, rather than waiting for treatment failure.

One of the key mediators of colonic inflammation in ASUC is interleukin-1 (IL-1) which plays a pivotal role in local activation of neutrophils and a number of downstream inflammatory mediators.[15] The IL-1 axis has been repeatedly identified as a therapeutic target in UC.[16–18] IL-1 antagonism occurs naturally via the ubiquitous IL-1 receptor antagonist (IL-1Ra). A recombinant form of this naturally-occurring anti-inflammatory, anakinra (Kineret®), has been used for the treatment of patients with rheumatoid arthritis and crypopyrin associated periodic syndromes (CAPS) with good effect.[19–22] Anakinra has a very good safety profile in many disease states including RA, CAPS, severe sepsis and intracranial haemorrhage at standard subcutaneous (SC) dosing regimens and using high dose IV administration regimens.[21,23,24]

Our hypothesis is that antagonism of the IL-1 axis with anakinra is an effective way of abrogating the early inflammatory response seen in ASUC and leads to improved clinical outcomes.

There is evidence to suggest that anakinra co-treatment with corticosteroids may be beneficial, given that corticosteroids suppress the production of IL-1Ra to a greater extent than its active family members – IL-1α and IL-1β.[25] Moreover, while recent research has seen a trend towards antagonism of IL-1 using monoclonal antibodies,[17] anakinra may offer some advantages. Firstly, its relative short half-life (4-6h) compared with antibodies allows early institution of further immunosuppression therapy if rescue therapy is required. Secondly,

For peer review only - http://bmjopen.bmj.com/site/about/guidelines.xhtml

anakinra antagonises both IL-1α and IL-1β, while monoclonal antibodies are specific to either form. Finally, given that the patent for anakinra is now expired, the cost of the product is much less than that of the monoclonal antibody alternatives[26,27]. Given this evidence, antagonism of IL-1 mediated inflammation early in the course of inflammation, in conjunction with standard corticosteroid treatment, is a promising therapeutic avenue in patients with ASUC.

Aims & Objectives

IASO aims to test whether anakinra can reduce the need for medical or surgical rescue therapy in patients with ASUC when given alongside IV corticosteroids. The trial also aims to assess the effects of anakinra on disease biology and bowel inflammation in addition to assessing drug safety, and longer-term health and patient-reported quality of life outcomes. Finally, the trial will seek to advance scientific understanding of ASUC through the collection and analysis of a large range of biological samples, including blood and tissue transcriptomic analysis, as well as microbial analysis. As part of this work, we will attempt to validate a transcriptional predictor of outcomes in UC that we have previously reported.[28]

Primary Objective

To compare the clinical effects of anakinra with placebo when given in addition to current standard care in patients with ASUC.

Secondary Objective

To compare the effects of anakinra with placebo when given in addition to current standard care to patients with ASUC in terms of:

- Safety
- Need for colectomy
- Patient-reported outcomes
- Endoscopic and histologic evidence of treatment effects
- Evidence of disruption of the IL-1 signalling pathway

This article summarises the approved trial protocol in use at the time of publication (version 2.0 dated 25 May 2018). The full version of the protocol can be found in the supplementary appendix. The most current version of the protocol will be made available online at https://www.journalslibrary.nihr.ac.uk/programmes/eme/1420102/#/.

For peer review only

For peer review only - http://bmjopen.bmj.com/site/about/guidelines.xhtml

Methods

Trial Design & Flowchart

IASO is a multi-centre, two arm (parallel group), randomised, placebo-controlled, double-blinded trial. The trial will also feature a sub-study specifically examining the endoscopic effects of treatment with anakinra in comparison with placebo. A summary of the trial design is shown in *Figure 1*.

[Figure 1]

Figure 1. IASO Trial Flowchart.

**Data collection for the primary endpoint and the colectomy-specific secondary endpoint is performed at days measured relative to the start of IMP treatment. However, the subsequent data analysis will assess these endpoints using time relative to the earlier start of IV corticosteroid treatment.*

Centres

IASO is a multi-centre study in UK NHS acute hospitals. We plan to include approximately 20 centres in the trial.

All sites will recruit, treat and follow up participants equally, but a subset of sites will also recruit participants into the optional endoscopic sub-study involving one additional flexible sigmoidoscopy with biopsy collection.

Trial duration

Participants' involvement in the trial will last approximately 6 months. This includes an initial treatment period of approximately 5–7 days, during which screening and an investigational medicinal product (IMP) treatment course of up to 5 days will be completed. Review of participant outcomes following trial participation may continue for up to 5 years using Hospital Episode Statistics (HES) data, without any additional burden to the participant. Any HES data collection would start at 1 year post-commencement of IMP treatment and examination of outcomes via HES data would potentially continue for up to 5 years, depending on study outcomes. A yearly assessment of HES outcomes by the TSC would determine whether collection of HES data should continue.

Participant Selection

Inclusion Criteria

To be included in the trial the participant must:

- Be aged 16–80 years inclusive
- Have given written informed consent to participate
- Be hospitalised with clinically confirmed or suspected ASUC and a modified Truelove and Witts severity index (MTWSI)[7] score ≥11
- Have a requirement for treatment with IV corticosteroids in the judgement of the treating clinician, with the possibility to receive a first dose of IMP within 36 hours of commencement of IV corticosteroids

There are no validated clinical scoring systems for ASUC. We selected the MTWSI (also known as the Lichtiger index) with a cut-off of ≥11 since this aligns with the score used in index studies of ciclosporin in ASUC [7], as well as in the CySIF and CONSTRUCT trials comparing ciclosporin and infliximab [9,29]. The components of the MTWSI include those parameters that are relevant to clinical decision making for initial recognition of ASUC and for determination of the need for rescue therapy (see supplementary online material).

For peer review only - http://bmjopen.bmj.com/site/about/guidelines.xhtml

Exclusion Criteria

The presence of any of the following will preclude participant inclusion:

- Pregnant or breast-feeding women
- Oral corticosteroid dosing for a duration of 8 weeks or more immediately prior to commencement of IV corticosteroid dosing
- History of severe hepatic impairment (e.g. Child-Pugh = Grade C)
- Moderate or Severe renal impairment (estimated glomerular filtration rate [eGFR] <60ml/min/1.73m^2)
- Neutropenia (neutrophil count <1.5x10^9/l)
- Previous treatment with anakinra for any indication
- Documented hypersensitivity to the active substance or to any of the excipients or to E. coli derived proteins; latex allergy
- Evidence (from blood cultures etc) or clinical suspicion of systemic infection*
- Current or previous cytomegalovirus (CMV) infection requiring treatment with anti-viral agents
- Current treatment with anti-TNF-α therapy or anti-TNF-α discontinuation within previous 16 weeks
- A history of pulmonary TB infection
- Any absolute contraindication to IV corticosteroid
- History of malignancy (with the exception of non-melanoma skin cancer) or colonic dysplasia
- Rectal therapy in previous 14 days (sub-study exclusion only)
- Receipt of another IMP as part of a CTIMP within the previous 16 weeks

*Concurrent prescription of antibiotics to cover for the possibility of GI infection whilst awaiting stool culture results, or the possibility of bacterial translocation relating to severe colitis, is not an exclusion criterion where the physician suspects ulcerative colitis is the most likely diagnosis.

Outcome Measures

Primary Outcome

The primary outcome of the trial will be the incidence of medical (i.e. infliximab or ciclosporin) or surgical rescue therapy (colectomy) within 10 days following the commencement of intravenous corticosteroid therapy.

For the purposes of the primary endpoint analysis, the time point of the start of rescue therapy will be as follows:

- Medical rescue therapy: Date and approximate time of first treatment administration
- Surgical rescue therapy: Date and approximate time of start of surgery

Secondary Outcomes

1. Incidence of colectomy within 98 days following commencement of IV corticosteroid therapy.

For peer review only - http://bmjopen.bmj.com/site/about/guidelines.xhtml

2. Burden of disease activity, measured by daily MTWSI scores over Days 1-5 after initial IMP administration.
3. Time to clinical response (defined as 2nd consecutive day with MTWSI<10)*
4. Time to medical or surgical rescue therapy, measured according to the time after the first dose of IV corticosteroids until the time that rescue therapy occurs (using definitions as set out in the primary endpoint). Data will be captured up to the same time point as the primary endpoint.
5. Incidence of serious adverse events (SAEs), measured until Day 10 (+3) following commencement of IMP treatment.

*Time to clinical response is measured during the IMP treatment phase only

Exploratory Outcomes

1. Patient-reported quality of life (EuroQol five dimensions; EQ-5D[30] and Crohn's and ulcerative colitis questionnaire-32; CUCQ-32[31]) at baseline and at 3 and 6 months following commencement of IMP treatment.

   The EQ-5D is a widely used generic health-related quality of life measure featuring self-assessment questions distributed across 5 domains. The CUCQ-32 is an Inflammatory Bowel Disease (IBD)-specific questionnaire which captures information related to a patient's wellbeing during their daily routine. Both questionnaires have been validated[32,33].
2. Endoscopic response at Day 3 (± 1 day) (via standardised endoscopic scoring completed by a local and a central blinded assessor) following commencement of IMP treatment (endoscopic sub-study group only)

In addition to the above exploratory endpoints, the trial will also perform further exploratory analyses related to the identification of biomarkers and treatment response predictors, as well as characterisation of the stool microbial response to anakinra. Finally, patient readmission and colectomy data (e.g. via HES) may be examined to investigate the impact of treatment in the longer term.

Trial Treatment

For the purpose of this trial, the following are considered the IMP:

- Anakinra solution for injection
- Placebo to match anakinra solution for injection

IMP is supplied in commercial pre-filled syringe configurations (100mg/0.67ml), packed into trial-specific participant kits containing 11 pre-filled syringes. The syringes and kits have blinded labelling and are identified by unique pack (kit) numbers. IMP will be stored in accordance with the requirements of the SmPC for Anakinra.[34]

Participants will receive an initial loading dose of 100 mg IMP given intravenously (IV) within 36 hours of first IV corticosteroids. This will be followed by 100 mg SC IMP given twice daily, over a maximum of 5 days (maximum of 10 doses).

IV methylprednisolone or hydrocortisone, as per local guidelines for ASUC standard care, will be administered to participants as non-IMPs. Participants will continue to be treated with routine concomitant medications for other co-morbidities as appropriate within their current clinical condition, with modifications (e.g. oral to IV switches), or temporary cessation as necessary. Appropriate supportive care may also be prescribed, including fluids, nutritional supplements and other medications as necessary.

For peer review only - http://bmjopen.bmj.com/site/about/guidelines.xhtml

Participants will return to their normal standard of care as defined by their local physicians, following completion of trial treatment.

Treatment Termination & Trial Withdrawal

Participants may choose to withdraw from treatment or the full study at any stage without prejudice to standard clinical care.

Clinicians should cease treatment with the study drug according to their judgement or under any of the following criteria:

- Withdrawal of consent for treatment administration
- Development of renal impairment (eGFR <60 ml/min/1.73m^2) or severe hepatic impairment (Child-Pugh grade C)
- Development of neutropenia (neutrophil count <1.5x10^9/l)
- Detection of a significant GI pathogen in stool specimen
- Sufficient recovery to allow for cessation of IV steroids
- Hospital discharge due to recovery
- Decision to commence medical or surgical rescue therapy
- CMV reactivation necessitating treatment with anti-viral agents (in the judgement of treating clinicians)
- The development of a SAE necessitating termination of IMP treatment.

Where possible, trial assessments will continue following treatment termination.

In addition to the treatment termination criteria, the following criteria would result in participant withdrawal from the trial, with no further assessments being performed:

- Withdrawal of consent for further assessments and data collection
- Death

Randomisation & Stratification

All patients screened for the trial will be assigned a unique participant ID number. Suitable participants will subsequently be randomised (1:1) to drug:placebo using an online randomisation system accessible via password-protected access. Randomisation will be stratified for 2 variables:

- Previous or current therapy with any of: immunomodulators (azathioprine, 6-mercaptopurine, 6-thioguanine, methotrexate, ciclosporin) biologics (anti-TNF-α monoclonal antibodies, anti-adhesion molecule antibodies, other anti-cytokine antibodies) or oral janus kinase inhibitors
- Current or previous oral corticosteroid prescription(s) within 8 weeks prior to first dose of intravenous corticosteroids

Blinding

Trial participants, research teams and Site pharmacies will be blinded to the treatment group for the duration of the trial.

For peer review only - http://bmjopen.bmj.com/site/about/guidelines.xhtml

Procedures & Assessments

Participant Identification & Informed Consent

We will recruit inpatients with ASUC. Identification of potential participants will be undertaken by the clinical team with subsequent consenting and trial-specific procedures carried out by the research team.

A total of 40 individuals will be asked to join an optional scientific sub-study cohort. Participants can decline to participate in the sub-study but still participate in the main study.

Anonymised data on all participants who are approached will be collated in accordance with Consolidated Standards of Reporting Trials (CONSORT) guidelines.

Screening

The tests required for screening all form part of standard clinical care, therefore no additional tests will be performed other than a urine β-human chorionic gonadotropin (β-hCG) test for females where there is a possibility of pregnancy.

Baseline

The following activities will be performed and recorded at baseline:

- Medical history review
- Review of blood parameters (haemoglobin, total white blood cell, neutrophil, lymphocyte, monocyte, platelet, albumin and C-reactive protein) following a blood test performed as part of standard care
- 3 research blood samples (1 plasma tube, 1 nucleic acid tube, 1 genotyping tube) as described in the laboratory manual
- Concomitant medications review
- Demographics
- MTWSI assessment
- Stool sample collection
- EQ-5D and CUCQ-32 questionnaires

Randomisation & Post-Baseline Procedures

Following baseline data collection, participants will be randomised and allocated treatment as described above.

Prior to the first administration of IMP (Day 0), the following assessments will be performed:

- Review of adverse events (AEs)
- Recording of any changes to concomitant medication since the last assessment
- Confirmation that IMP administration can proceed, according to the following criteria:

    The time of administration of the first IMP dose falls no later than 36 hours following the time of first dose of IV corticosteroid.

    IV corticosteroid therapy remains ongoing at the time of administration of the first IMP dose (i.e. that a decision to discontinue corticosteroids or transition to oral steroids has not been made after the time of participant consent to participate in the trial and before the time of the administration of the first IMP dose).

    A firm decision to start rescue therapy or perform emergency colectomy has not already been taken

For peer review only - http://bmjopen.bmj.com/site/about/guidelines.xhtml

During the days subsequent to the first dose of trial medication, the following activities will be recorded for daily up to and including Day 5 following IMP treatment initiation:

- MTWSI assessment / review of standard care clinical MTWSI assessment domains#
- Recording of any changes to concomitant medication prescription since the last assessment
- Review of blood parameters (haemoglobin, total white blood cell, neutrophil, lymphocyte, monocyte, platelet, albumin and C-reactive protein) following blood tests performed as part of standard care
- 2 research blood samples (1 plasma tube, 1 nucleic acid tube)*
- Review of AEs
- Medical or surgical (emergency colectomy) rescue therapy assessment
- Stool sample collection on Day 5 (± 1 day) following commencement of IMP.

#Not recorded for patients who have undergone colectomy

*A minimum of 1 sample every 72 hours should be taken. Wherever possible every effort should be made to obtain the samples every day (up to 10 samples).

Sub-Study-Only Assessments

In addition to the daily assessments described above for Days 1-5, the scientific sub-study group will undergo an additional optional flexible sigmoidoscopy (without bowel preparation) on Day 3 (± 1 day) following commencement of IMP. This will be performed during their index inpatient admission.

Findings will be recorded according to a standardised endoscopic scoring system (Mayo)[35] by local and central blinded assessors. Histology will be scored on up to a maximum of 6 biopsies taken from each of the rectum and sigmoid colon, according to validated scoring systems for colitis severity at the end of the trial.[36–38]

Where possible, endoscopic scores, written summary reports and photographic recordings (video or static images) of sigmoidoscopies will be sent to the central trial team for verification of local scoring. In addition, where available, data collected as part of standard care (non-sub-study) endoscopies will be used to supplement trial-specific data. These will be scored by the blinded central assessor.

Follow-Up

Following the participants' inpatient stay, the following assessments will occur:

- Day 10 (+3) following commencement of IMP treatment – Rescue therapy assessment*
- Day 10 (+3) following commencement of IMP treatment – AE monitoring
- Day 98 (+14) following commencement of IMP treatment – Colectomy assessment†
- Approximately 3 and 6 months following commencement of IMP treatment – EQ-5D and CUCQ-32 questionnaires.

*In the event that a participant is discharged prior to Day 10 without having started rescue therapy, a member of the trial team will contact the participant on days Day 10 (+3 days) to confirm that the participant remains out of hospital and has not received rescue therapy elsewhere since discharge. If the participant is not contactable, local hospital records will be checked for admission.

†If not previously recorded, a member of the trial team will contact the participant on Day 98 (+14) to confirm that the participant remains out of hospital and has not undergone a

For peer review only - http://bmjopen.bmj.com/site/about/guidelines.xhtml

colectomy since discharge. If the participant is not contactable, local hospital records will be checked for admission.

Participant status following discharge, including readmissions and subsequent surgery may be assessed using patient-specific HES data from the NHS Digital, Data Access Request Service. Any data collection would start at 1 year post-commencement of IMP treatment and examination of outcomes via HES data would potentially continue for up to 5 years, depending on study outcomes.

Safety Monitoring & Reporting

This section summarises the safety monitoring and reporting processes for the trial. Full details of the safety processes can be found in the comprehensive protocol included as part of the supplementary appendix.

AEs will be recorded from the point of Informed Consent. Standard regulatory definitions for assessing AE seriousness will be used.[39]

As the IMP has a short half-life (<6h) no cumulative or late effects are anticipated. Therefore, recording of AEs and reporting of SAEs will be actively monitored up to Day 10 (+3 days). Following the end of the active monitoring period, investigators are still required to report any Serious Adverse Reactions (SARs) or Suspected Unexpected Serious Adverse Reactions (SUSARs) of which they become aware.

Pregnancy reporting is only required for trial participants whilst they are receiving IMP and for up to 24 hours post their last dose.

Patient and Public Involvement

Patient involvement was actively sought during the planning and preparation stage for this trial, and will form a key part of the trial as it progresses.

A 10-member patient steering group assisted with trial design in an early workshop, reviewed previous versions of the trial protocol and assisted with responses to the initial funding application review. The group provided valuable insight into the acceptability of different dosing regimens and into ways to support patients during the consent process. The patient information leaflet was reviewed by the group. To ensure that the views of the wider patient community were sought, an online questionnaire regarding willingness to participate in inpatient research studies in colitis was circulated to members of Crohn's and Colitis UK and responses used to inform trial design.

A patient representative will serve as a member of the TSC.

Statistics & Data Management

Sample Size

We plan to include approximately 214 participants (107 per group) in the trial with 40 participants also included in the optional endoscopic sub-study. Recruitment of 214 patients to the main trial will give 85% power to detect a 20% absolute risk reduction (ARR) in the primary endpoint from a rate in the control group of 49% testing at the 5% significance level.

We have based our estimate of the primary endpoint rate on data from the recently completed NIHR-funded CONSTRUCT trial.[40] This trial recruited 270 patients with ASUC across 52 UK sites between 2010–2013. In CONSTRUCT, 49% of patients admitted with ASUC needed escalation to medical rescue therapy following treatment with corticosteroids (Prof J Williams, personal communication). Since there are no existing estimates of effect size for anakinra in ASUC, we chose to power IASO to detect a 20% ARR following protocol review by members of the British Society of Gastroenterology IBD Clinical Research Group, who advised that this was

For peer review only

For peer review only - http://bmjopen.bmj.com/site/about/guidelines.xhtml

the minimum effect size needed that would make a substantial difference to the patient population, and which would be broadly in line with effect sizes seen in other drugs for moderate to severe colitis.[41–43]

The scientific sub-study based on testing cytokines will be performed on 40 participants. The choice of sample size is based on power studies in transcriptomic studies in UC[44] with adjustment for multiple testing, as well as pragmatic grounds of cost.

Patient Population

Populations to be analysed in the trial are as follows:

- Full Analysis Population: All randomised participants who receive at least one dose of IMP
- Randomised Population: All randomised participants, regardless of whether IMP was received
- Safety Population: All consenting participants

Any further populations may additionally be defined within the Statistical Analysis Plan.

Analyses

Primary Analysis

We will report summary statistics on the primary and secondary endpoints according to treatment group. The primary analysis will consist of an estimate, 95% confidence interval and p-value of the absolute risk difference of the incidence rates of the need for medical or surgical rescue therapy within 10 days following the first administration of IV corticosteroids between the two treatment arms, using logistic regression to adjust for important baseline covariates. The primary efficacy analysis will be based upon the Full Analysis Population.

The baseline characteristics will include:

- Prior diagnosis of IBD at the point of hospitalisation (‘first presentation’)
- Prior hospitalisation for ASUC
- Previous or current therapy with any of: immunomodulators (azathioprine, 6-mercaptopurine, 6-thioguanine, methotrexate, ciclosporin) biologics (anti-TNF-α monoclonal antibodies, anti-adhesion molecule antibodies, other anti-cytokine antibodies) or oral janus kinase inhibitors
- Current or previous oral corticosteroid prescription(s) within 8 weeks prior to first dose of intravenous corticosteroids
- Demographics

Secondary endpoints will be compared in a similar regression model to estimate the treatment effect on an appropriate scale of comparison. This will include an assessment of the effects of treatment with anakinra on the secondary endpoints.

Secondary Analyses

The following secondary analyses will be performed in the trial:

- Analysis of the Randomised Population
- Complier Average Causal Effect analysis to assess the influence of the amount of treatment received, as distinct from treatment assigned

To test the hypothesis that the clinical effects of anakinra in ASUC may differ between groups of patients with differing levels of prior inflammatory burden, pre-specified subgroup analyses, in

For peer review only - http://bmjopen.bmj.com/site/about/guidelines.xhtml

the form of estimating treatment-covariate interactions, will be performed on the following baseline characteristics:

- Prior diagnosis of IBD at the point of hospitalisation ('first presentation')
- Prior hospitalisation for ASUC
- Naïvety to any of: immunomodulators (azathioprine, 6-mercaptopurine, 6-thioguanine, methotrexate, ciclosporin) biologics (anti-TNF-α monoclonal antibodies, anti-adhesion molecule antibodies, other anti-cytokine antibodies) or oral janus kinase inhibitors at the point of hospitalisation.
- Receipt of oral corticosteroids within 8 weeks prior to first dose of intravenous corticosteroids
- Cases of suspected or confirmed ASUC without evidence of CMV reactivation requiring treatment with anti-viral agents and without evidence of a significant GI pathogen
- Duration between 1st dose of IV corticosteroids to 1st dose of IMP.

Interim Analyses

As part of planned interim analyses, an independent Data Monitoring Committee (iDMC) will examine unblinded data and provide a recommendation to a Trial Steering Committee (TSC) as to whether the trial should continue at two pre-specified points detailed below.

After the first 20 participants have been recruited, and all completed a minimum of 10 days follow up (to the time of the primary endpoint), trial feasibility will be assessed. The trial will be regarded as feasible only if all of the following measures are met:

1. ≥10% of eligible participants are recruited (based upon use of screening logs to determine the percentage of eligible participants that are randomised in each site)
2. Adherence to dosing as per protocol (≥80% of participants received appropriately prescribed doses within any given 24h window)
3. Rescue therapy started on or before the 7th day following initial IV corticosteroid administration in ≥80% cases where required during the index admission (as determined by review of timing of rescue therapy administration relative to corticosteroid administration by trial investigators)
4. Maintenance of blinding: there should be no evidence of unnecessary unblinding of participants or investigators.
5. No serious safety concerns identified, especially with regard to infections or SUSARs.

After the first 100 participants have completed Day 10 assessments, futility analysis will be performed to test the hypothesis that the reduction in absolute risk difference in the primary outcome is ≥10% using a 1-sided 2.5% significance test using logistic regression on the absolute risk reduction scale. If the hypothesis is rejected, i.e. the lower limit of a 95% 2-sided confidence interval for Treatment - Control is above -10%, then the iDMC will consider the recommendation for the study to stop early for futility. This is equivalent to spending 0.1% from the 15% total beta value set to control the type 2 error under the alternative hypothesis (49% and 29% rates).[45] If the study continues past the interim, then the final analysis will not be adjusted for the futility analysis, and standard p-values and confidence intervals will be reported.

Data Management

All outcome data will be transferred onto a Case Report Form (CRF) which will be anonymised. Data captured on CRFs will subsequently be stored in a central database prior to analysis.

For peer review only - http://bmjopen.bmj.com/site/about/guidelines.xhtml

Personal identifiable data kept for the purposes of questionnaire postage and HES-data capture will be destroyed at the end of the trial.

Missing Data

Due to short and intensive period of follow up for the primary endpoint, we do not anticipate significant missing data. Where we are unable to obtain missing data within 12 weeks of the 3- or 6-month time points, we will employ the standard approaches to management of missing quality of life data previously described in the CONSTRUCT trial (e.g. participant death at questionnaire completion will be recorded as an EQ-5D score of 0 and match minimum-observed scores for other quality of life domains).[40]

For participants who withdraw consent to treatment, permission to continue to acquire data for outcome analysis will be sought. For those who do not consent to ongoing monitoring, including those who wish to withdraw entirely from the study, existing data acquired to the point of withdrawal will be included in the final study analysis, with missing data handled according to standard missing data methods, including missing-at-random (MAR) methods with sensitivity testing for deviation from MAR assumptions.

Trial End

For regulatory notification purposes, the end of trial will be the earliest of:

- The receipt of the final, returned 6-month quality of life questionnaires
- 6 months (+12 weeks) after the last participant entered the study (date of questionnaire censoring).

However, participants may enter a period of long-term follow-up via HES data examination and the trial would remain open to the Ethics Committee until the last participant's HES data capture has been completed.

Ethics & Dissemination

IASO has received ethical (17/EE/0347) and Health Research Authority approval. The trial is being carried out under a Clinical Trial Authorisation from the MHRA.

This is an NIHR funded trial and the NIHR publishing guidelines will be followed. Authorship of final study outputs will be assigned in accordance with guidelines set out by the International Committee of Medical Journal Editors. Plain language summaries of key trial outputs will be prepared in conjunction with the patient steering group and published on the trial website. This website is listed in the patient information leaflet for the benefit of all trial participants.

Sponsorship & Funding

The trial is sponsored by Cambridge University Hospitals NHS Foundation Trust and University of Cambridge.

This project is funded by the Efficacy and Mechanism Evaluation (EME) Programme, an MRC and NIHR partnership.* The views expressed in this publication are those of the author(s) and not necessarily those of the MRC, NHS, NIHR or the Department of Health.

*The EME Programme is funded by the MRC and NIHR, with contributions from the CSO in Scotland and NISCHR in Wales and the HSC R&D Division, Public Health Agency in Northern Ireland.

The trial drug is being provided by Swedish Orphan Biovitrum.

The Wellcome Trust Sanger Institute are providing financial support and academic collaboration for sample sequencing.

For peer review only - http://bmjopen.bmj.com/site/about/guidelines.xhtml

Data Sharing

Anonymised datasets generated during and/or analysed during the trial will be stored in a publically available repository. Following completion of the trial and the analysis, it is anticipated that de-identified research data including sequencing data will be made available for other researchers. This will include use of database repositories such as Gene Expression Omnibus (GEO, https://www.ncbi.nlm.nih.gov/geo/), as well as sharing of participant-level datasets that underlie the results reported in published articles. Any clinical data will be shared with researchers who provide a methodologically sound proposal in order to achieve aims in the approved proposal.

Author Contributions

The full protocol was written by Martin Thomas, Tim Raine, Arthur Kaser, Carrie Bayliss and Francis Dowling. Sections related to Statistics and Data Analysis were written by Simon Bond. The Trial Treatment Section was written by Lynne Whitehead.

The study design was conceived by Tim Raine with intellectual input provided by Arthur Kaser, Miles Parkes, Alastair Watson, Simon Bond, Vipul Jairath, John Williams, Christopher Probert, James Galea, Elizabeth Timperley-Preece and Christopher Lamb.

Acknowledgements

The authors would like to thank the Cambridge Clinical Trials Unit for its support during the setup and conduct of the trial. The authors would also like to thank the patient steering group for its assistance with the trial design and for its ongoing involvement in the trial.

Christopher Lamb is a Clinical Lecturer supported by the NIHR.

Competing Interests

The authors have no competing interests to declare.

Implications of Pragmatic Trial Design [*supplementary text box*]

In designing the IASO trial, we have followed a "pragmatic" approach to trial design. This aims to test our intervention under conditions as close to real-life routine practice as possible and so produce results that can be generalised and applied in routine practice settings. Another important aspect to consider was that, since the intervention targets an early molecule in the inflammatory cascade, and since there are already well-established options for rescue therapy, we wanted to test the intervention as close as possible to the point of recognition of a patient by a treating physician as having ASUC. If the intervention is shown to have a positive outcome, then the generalisation of the trial findings might include consideration of initiation of anakinra dosing at the same time as intravenous corticosteroids i.e. as part of initial therapy.

For these reasons, we aimed to keep our inclusion criteria as broad as possible and minimise exclusion criteria. Thus, in essence, almost any patient with ASUC capable of giving informed consent can participate in IASO. The key criterion is the recognition of ASUC by a physician and the judgement that intravenous corticosteroids are needed. The use of the MTWSI serves as a 'sense-check' on this physician judgement to ensure that patients with too low a burden of inflammation or symptoms are not included. We considered and rejected other inclusion criteria that might be typically seen in 'explanatory' trials in IBD, such as the use of video-endoscopy with local or centralised scoring, or faecal calprotectin measurement. Although local assessment of the mucosa is widely used in the assessment of patients with ASUC, in many centres

For peer review only - http://bmjopen.bmj.com/site/about/guidelines.xhtml

intravenous corticosteroids may be started before this point. Furthermore, the use of centralised scoring would introduce delays into participant screening to the extent that randomisation and dosing would be impossible to achieve prior to the point where rescue therapy should be considered. The use of physician judgement as the main arbiter for inclusion does risk the inclusion of patients who subsequently turn out to have, for example, Crohn's colitis or even infectious colitis, just as does happen in real life with decisions around corticosteroid initiation. We can, of course, obtain endoscopic data as well as clinical and histologic data where available and these are indeed built into our analysis plans. But ultimately, IASO seeks to address whether a physician encountering a patient who he or she believes to have ASUC, *on the basis of the evidence available*, can and should prescribe anakinra in addition to corticosteroids. In this regard, more detailed inclusion criteria would not produce a generalizable result, whilst jeopardising timely patient identification and trial recruitment.

References

1 Molodecky NA, Soon IS, Rabi DM, *et al.* Increasing incidence and prevalence of the inflammatory bowel diseases with time, based on systematic review. *Gastroenterology* 2012;**142**:46–54 e42; quiz e30. doi:10.1053/j.gastro.2011.10.001

2 Lynch RW, Lowe D, Protheroe a, *et al.* Outcomes of rescue therapy in acute severe ulcerative colitis: data from the United Kingdom inflammatory bowel disease audit. *Aliment Pharmacol Ther* 2013;**38**:935–45. doi:10.1111/apt.12473

3 Dinesen LC, Walsh AJ, Protic MN, *et al.* The pattern and outcome of acute severe colitis. *J Crohn's Colitis* 2010;**4**:431–7. doi:10.1016/j.crohns.2010.02.001

4 NELA Project Team. Table 36. Third patient report of the National Emergency Laparotomy Audit. Published Online First: 2017.http://www.nela.org.uk/reports

5 Harbord M, Eliakim R, Bettenworth D, *et al.* Third European evidence-based consensus on diagnosis and management of ulcerative colitis. Part 2: Current management. *J Crohn's Colitis* 2017;**11**:769–84. doi:10.1093/ecco-jcc/jjx009

6 Truelove SC, Witts LJ. Cortisone in Ulcerative Colitis Final Report on a Therapeutic Trial. *Br Med J* 1955;**2**:1041–8. doi:10.1136/bmj.2.4947.1041

7 Lichtiger S, Present DH, Kornbluth A, *et al.* Cyclosporine in Severe Ulcerative Colitis Refractory to Steroid Therapy. *N Engl J Med* 1994;**330**:1841–5. doi:10.1056/NEJM199406303302601

8 Järnerot G, Hertervig E, Friis-Liby I, *et al.* Infliximab as rescue therapy in severe to moderately severe ulcerative colitis: A randomized, placebo-controlled study. *Gastroenterology* 2005;**128**:1805–11. doi:10.1053/j.gastro.2005.03.003

9 Williams JG, Alam MF, Alrubaiy L, *et al.* Infliximab versus ciclosporin for steroid-resistant acute severe ulcerative colitis (CONSTRUCT): a mixed methods, open-label, pragmatic randomised trial. *Lancet Gastroenterol Hepatol* 2016;**1**:15–24. doi:10.1016/S2468-1253(16)30003-6

10 Lee J. Use of Antioxidants to Prevent Cyclosporine A Toxicity. *Toxicol Res* 2010;**26**:163–70. doi:10.5487/TR.2010.26.3.163

11 Mason J. The pathophysiology of Sandimmune (cyclosporine) in man and animals. *Pediatr Nephrol* 1990;**4**:686–704.

12 de Mattos AM, Olyaei AJ, Bennett WM. Nephrotoxicity of immunosuppressive drugs: long-term consequences and challenges for the future. Am. J. Kidney Dis. 2000;**35**:333–46.

13 Nankivell BJ, Borrows RJ, Fung CL-S, *et al.* The natural history of chronic allograft nephropathy. *N Engl J Med* 2003;**349**:2326–33. doi:10.1056/NEJMoa020009

For peer review only - http://bmjopen.bmj.com/site/about/guidelines.xhtml

For peer review only

14 National Institute for Health and Care Excellence. Costing statement: Ulcerative colitis Implementing the NICE guidance on infliximab, adalimumab and golimumab for treating moderately to severely active ulcerative colitis after the failure of conventional therapy (TA329). 2015. *https://www.nice.org.uk/guidance/ta329/resources/costing-statement-pdf-428356477*

15 Dinarello CA. Interleukin-1 in the pathogenesis and treatment of inflammatory diseases. *Blood* 2011;**117**:3720–32. doi:10.1182/blood-2010-07-273417

16 Planell N, Lozano JJ, Mora-Buch R, *et al.* Transcriptional analysis of the intestinal mucosa of patients with ulcerative colitis in remission reveals lasting epithelial cell alterations. *Gut* 2013;**62**:967–76. doi:10.1136/gutjnl-2012-303333

17 Dinarello CA, Simon A, van der Meer JWM. Treating inflammation by blocking interleukin-1 in a broad spectrum of diseases. *Nat Rev Drug Discov* 2012;**11**:633–52. doi:10.1038/nrd3800

18 Cominelli F, Pizarro TT. Interleukin-1 and interleukin-1 receptor antagonist in inflammatory bowel disease. *Aliment Pharmacol Ther* 1996;**10 Suppl 2**:49–53; discussion 54.http://www.ncbi.nlm.nih.gov/pubmed/8899101

19 Cohen S, Hurd E, Cush J, *et al.* Treatment of rheumatoid arthritis with anakinra, a recombinant human interleukin-1 receptor antagonist, in combination with methotrexate: results of a twenty-four-week, multicenter, randomized, double-blind, placebo-controlled trial. *Arthritis Rheum* 2002;**46**:614–24. doi:10.1002/art.10141

20 Cohen SB, Moreland LW, Cush JJ, *et al.* A multicentre, double blind, randomised, placebo controlled trial of anakinra (Kineret), a recombinant interleukin 1 receptor antagonist, in patients with rheumatoid arthritis treated with background methotrexate. *Ann Rheum Dis* 2004;**63**:1062–8. doi:10.1136/ard.2003.016014

21 Kullenberg T, Löfqvist M, Leinonen M, *et al.* Long-term safety profile of anakinra in patients with severe cryopyrin-associated periodic syndromes. *Rheumatol (United Kingdom)* 2016;**55**:1499–506. doi:10.1093/rheumatology/kew208

22 Bresnihan B, Alvaro-Gracia JM, Cobby M, *et al.* Treatment of rheumatoid arthritis with recombinant human interleukin-1 receptor antagonist. *Arthritis Rheum* 1998;**41**:2196–204. doi:10.1002/1529-0131(199812)41:12<2196::AID-ART15>3.0.CO;2-2

23 Opal SM, Fisher CJ, Dhainaut JF, *et al.* Confirmatory interleukin-1 receptor antagonist trial in severe sepsis: a phase III, randomized, double-blind, placebo-controlled, multicenter trial. The Interleukin-1 Receptor Antagonist Sepsis Investigator Group. *Crit Care Med* 1997;**25**:1115–24. doi:10.1097/00003246-199707000-00010

24 Fleischmann R, Tesser J, Schiff MH, *et al.* Safety of extended treatment with anakinra in patients with rheumatoid arthritis. *Ann Rheum Dis* 2006;**65**:1006–12. doi:10.1136/ard.2005.048371

25 Langereis JD, Oudijk EJD, Schweizer RC, *et al.* Steroids induce a disequilibrium of secreted interleukin-1 receptor antagonist and interleukin-1Beta synthesis by human neutrophils. *Eur Respir J* 2011;**37**:406–15. doi:10.1183/09031936.00170409

26 British National Formulary. Canakinumab - Solution for injection. *https://bnf.nice.org.uk/medicinal-forms/canakinumab.html*

27 British National Formulary. Anakinra - Solution for injection. *https://bnf.nice.org.uk/medicinal-forms/anakinra.html*

28 Lee JC, Lyons P a, McKinney EF, *et al.* Gene expression profiling of CD8+ T cells predicts prognosis in patients with Crohn disease and ulcerative colitis. *J Clin Invest* 2011;**121**:4170–9. doi:10.1172/JCI59255

29 Laharie D, Bourreille A, Branche J, *et al.* Ciclosporin versus infliximab in patients with severe ulcerative colitis refractory to intravenous steroids: a parallel, open-label randomised controlled trial. *Lancet (London, England)* 2012;**380**:1909–15.

For peer review only - http://bmjopen.bmj.com/site/about/guidelines.xhtml

doi:10.1016/S0140-6736(12)61084-8

30 EuroQol - a new facility for the measurement of health-related quality of life. *Health Policy (New York)* 1990;**16**:199–208. doi:10.1016/0168-8510(90)90421-9

31 Alrubaiy L, WY C, Dodds P, *et al.* Development of a short questionnaire to assess the quality of life in Crohn's disease and ulcerative colitis. *J Crohns Colitis* 2015;**9**:66–76. doi:10.1093/ecco-jcc/jju005

32 Stark RG, Reitmeir P, Leidl R, *et al.* Validity, reliability, and responsiveness of the EQ-5D in inflammatory bowel disease in Germany. *Inflamm Bowel Dis* 2010;**16**:42–51. doi:10.1002/ibd.20989

33 Hutchings HA, Alrubiay L, Watkins A, *et al.* Validation of the Crohn's and Ulcerative Colitis questionnaire in patients with acute severe ulcerative colitis. *United Eur Gastroenterol J* 2017;**5**:571–8. doi:10.1177/2050640616671627

34 European Medicines Agency. Kineret (Anakinra) Summary of Product Characteristics. Available at: https://www.medicines.org.uk/emc/product/559.

35 Schroeder KW, Tremaine WJ, Ilstrup DM. Coated Oral 5-Aminosalicylic Acid Therapy for Mildly to Moderately Active Ulcerative Colitis. *N Engl J Med* 1987;**317**:1625–9. doi:10.1056/NEJM198712243172603

36 Geboes K, Riddell R, Öst A, *et al.* A reproducible grading scale for histological assessment of inflammation in ulcerative colitis. *Gut* 2000;**47**:404–9. doi:10.1136/gut.47.3.404

37 Marchal-Bressenot A, Salleron J, Boulagnon-Rombi C, *et al.* Development and validation of the Nancy histological index for UC. *Gut* 2017;**66**:43–9. doi:10.1136/gutjnl-2015-310187

38 Mosli MH, Feagan BG, Zou G, *et al.* Development and validation of a histological index for UC. *Gut* 2017;**66**:50–8. doi:10.1136/gutjnl-2015-310393

39 European Medicines Agency. NOTE FOR GUIDANCE ON CLINICAL SAFETY DATA MANAGEMENT: DEFINITIONS AND STANDARDS FOR EXPEDITED REPORTING (CPMP/ICH/377/95). Available at: http://www.ema.europa.eu/docs/en_GB/document_library/Scientific_guideline/2009/09/WC500002749.pdf. 1995.

40 Williams JG, Alam MF, Alrubaiy L, *et al.* Comparison Of iNfliximab and ciclosporin in STeroid Resistant Ulcerative Colitis: pragmatic randomised Trial and economic evaluation (CONSTRUCT). *Health Technol Assess* 2016;**20**:1–320. doi:10.3310/hta20440

41 Rutgeerts P, Sandborn WJ, Feagan BG, *et al.* Infliximab for Induction and Maintenance Therapy for Ulcerative Colitis. *N Engl J Med* 2005;**353**:2462–76. doi:10.1056/NEJMoa050516

42 Feagan BG, Rutgeerts P, Sands BE, *et al.* Vedolizumab as Induction and Maintenance Therapy for Ulcerative Colitis. *N Engl J Med* 2013;**369**:699–710. doi:10.1056/NEJMoa1215734

43 Sandborn WJ, Su C, Sands BE, *et al.* Tofacitinib as Induction and Maintenance Therapy for Ulcerative Colitis. *N Engl J Med* 2017;**376**:1723–36. doi:10.1056/NEJMoa1606910

44 Boland BS, Boyle DL, Sandborn WJ, *et al.* Validated gene expression biomarker analysis for biopsy-based clinical trials in ulcerative colitis. *Aliment Pharmacol Ther* 2014;**40**:477–85. doi:10.1111/apt.12862

45 DeMets DL, Lan KK. Interim analysis: the alpha spending function approach. *Stat Med* 1994;**13**:1341–6.

For peer review only

For peer review only - http://bmjopen.bmj.com/site/about/guidelines.xhtml

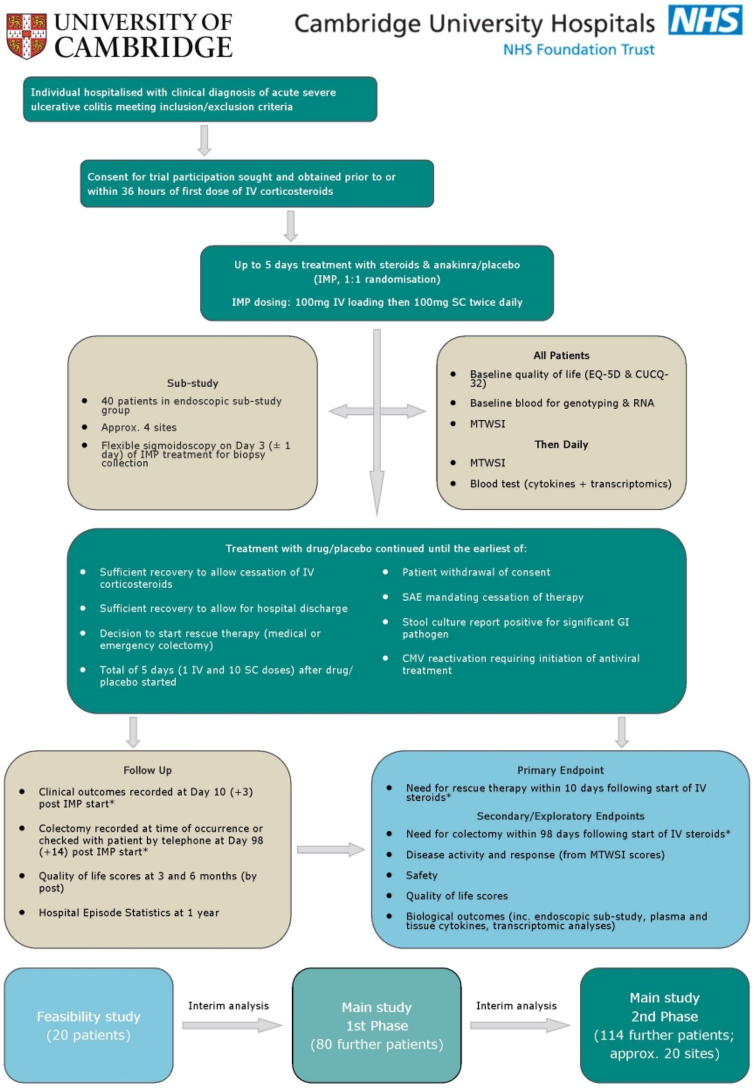

Figure 1. IASO Trial Flowchart. *Data collection for the primary endpoint and the colectomy-specific secondary endpoint is performed at days measured relative to the start of IMP treatment. However, the subsequent data analysis will assess these endpoints using time relative to the earlier start of IV corticosteroid treatment.CMV: Cytomegalovirus, CUCQ-32: Crohn's and ulcerative colitis questionnaire-32, EQ-5D: Euroqol 5 Dimensions, GI: Gastrointestinal, IMP: Investigational medicinal product, IV: Intravenous, MTWSI: Modified Truelove and Witt Severity Index, RNA: Ribonucleic Acid, SAE: Serious Adverse Event , SC: Subcutaneous

209x297mm (300 x 300 DPI)

Clinical Trial Protocol

IASO

Trial Title:	A phase II randomised placebo controlled double blinded trial of **I**nterleukin 1 blockade in **A**cute **S**evere C**o**litis
Protocol Number:	CCTU0165
EudraCT Number:	2017-001389-10
ISRCTN Number:	43717130
Investigational Product:	Anakinra (Kineret®)
Protocol Version:	2.0

Chief Investigator:	Professor Arthur Kaser
CI Address:	Department of Medicine, Box 157, Addenbrooke's Hospital, Cambridge CB2 0QQ, UK
Telephone:	01223 331130
Trial Sponsor:	Cambridge University Hospitals NHS Foundation Trust and the University of Cambridge
Trial Funder:	National Institute for Health Research and Medical Research Council
SAE Reporting:	Cambridge Clinical Trials Unit (CCTU)
	Clinical Trial Coordinator: Dr Martin Thomas
	Telephone: 01223 254 920
	Fax: 01223 254247
	Email: add-tr.iaso@nhs.net

Cambridge University Hospitals NHS
NHS Foundation Trust

UNIVERSITY OF CAMBRIDGE

MRC Medical Research Council

Funded & supported by NIHR

For peer review only - http://bmjopen.bmj.com/site/about/guidelines.xhtml

For peer review only

1 Protocol Signatures:

I give my approval for the attached protocol entitled "A phase II randomised placebo controlled double blinded trial of **I**nterleukin 1 blockade in **A**cute **S**evere C**o**litis" version 2.0, dated 25 May 2018.

Chief Investigator

Name: Professor Arthur Kaser

Signature: ______________________________

Date: ____________________

Trial Statistician

Name: Dr Simon Bond

Signature: ______________________________

Date: ____________________

Site Signatures

I have read the attached protocol entitled "A phase II randomised placebo controlled double blinded trial of **I**nterleukin 1 blockade in **A**cute **S**evere C**o**litis" version 2.0, dated 25 May 2018and agree to abide by all provisions set forth therein.

I agree to comply with the conditions and principles of Good Clinical Practice as outlined in the European Clinical Trials Directives 2001/20/EC and 2005/28/EC, the Medicines for Human Use (Clinical Trials) Regulations 2004 (SI 2004/1031) and any subsequent amendments of the clinical trial regulations, the Sponsor's SOPs, and other regulatory requirements as amended.

Name: ______________________________

Signature: ______________________________

Date: ____________________

2 Protocol and amendment history

Amendment No.	Protocol version no.	Date	Details of changes made
1	1.1	18 September 2017	Modified Section 11.4 and changed units/value of eGFR as part of the response to the MHRA review.
2	1.2	13 October 2017	ISRCTN number added, emc website link removed from Section 11.1.1.8

For peer review only - http://bmjopen.bmj.com/site/about/guidelines.xhtml

For peer review only

3	2.0	25 May 2018	Update to the number of centres in the trial (Section 9.2)

For peer review only

For peer review only - http://bmjopen.bmj.com/site/about/guidelines.xhtml

3 Trial Management Committee(s) and Protocol Contributors

Chief Investigator	Prof Arthur Kaser, Department of Medicine, Box 157, Addenbrooke's Hospital, Cambridge CB2 0QQ, UK<br>01223 331130<br>ak729@cam.ac.uk
Lead Investigator	Dr Tim Raine, Department of Medicine, Box 157, Addenbrooke's Hospital, Cambridge CB2 0QQ, UK<br>01223 331130<br>tr223@cam.ac.uk
Trial Coordinator	Dr Martin Thomas, Cambridge Clinical Trials Unit, Addenbrooke's Hospital, Cambridge CB2 0QQ, UK<br>01223 254920<br>martin.thomas@addenbrookes.nhs.uk
Statistician	Dr Simon Bond Cambridge, Cambridge Clinical Trials Unit,<br>Addenbrooke's Hospital, Cambridge CB2 0QQ, UK<br>01223 596475<br>simon.bond@addenbrookes.nhs.uk
Trials Pharmacist	Dr Lynne Whitehead, Central Pharmacy, Addenbrooke's Hospital, Cambridge CB2 0QQ, UK<br>01223 216057<br>lynne.whitehead@addenbrookes.nhs.uk
Protocol Contributors	Dr Miles Parkes, Department of Medicine, Box 157, Addenbrooke's Hospital, Cambridge CB2 0QQ, UK<br><br>Prof Alastair Watson, Department of Medicine, University of East Anglia, Norwich NR4 7TJ, UK<br><br>Dr Christopher Lamb, Institute of Cellular Medicine, Newcastle University, Newcastle upon Tyne NE2 4HH, UK<br><br>Prof John Williams, Swansea University College of Medicine,<br>, Swansea SA2 8PP, UK<br><br>Prof Chris Probert, Department of Gastroenterology, University of Liverpool, Liverpool L69 3GE, UK<br><br>Dr Vipul Jairath, Nuffield Department of Medicine, University of Oxford, Headington, Oxford OX3 7BN, UK<br><br>Mr James Galea, University of Manchester, Salford Royal NHS Foundation Trust, Salford M6 8HD, UK
DMC chair	Dr Peter Irving, St Thomas' Hospital, Westminster Bridge Road, London, SE1 7EH, UK<br>+44 (0)207 188 2499<br>peter.irving@kcl.ac.uk
TSC chair	Dr Oliver Brain, John Radcliffe Hospital, Headley Way, Headington, Oxford OX3 9DU, UK<br>+44 (0)1865 228755<br>Oliver.Brain@ouh.nhs.uk

For peer review only - http://bmjopen.bmj.com/site/about/guidelines.xhtml

TSC Patient representative	Harriet Boswell
Central Blinded Edoscopy Assessor	Dr Tim Raine, Department of Medicine, Box 157, Addenbrooke's Hospital, Cambridge CB2 0QQ, UK<br>01223 331130<br>tr223@cam.ac.uk

For peer review only

For peer review only - http://bmjopen.bmj.com/site/about/guidelines.xhtml

Table of Contents

1 Protocol Signatures: 2
2 Protocol and amendment history 2
3 Trial Management Committee(s) and Protocol Contributors 4
Table of Contents 6
4 Abbreviations 8
5 Trial Synopsis 9
6 Trial Flow Chart 14
7 Introduction 15
7.1 Background 15
7.2 Clinical Data 15
8 Rationale for Trial 17
9 Trial Design 18
9.1 Statement of Design 18
9.2 Number of Centres 19
9.3 Number of Participants 19
9.4 Participants Trial Duration 19
9.5 Trial Objectives 19
9.6 Trial Outcome Measures 20
10 Selection and withdrawal of participants 21
10.1 Inclusion Criteria 21
10.2 Exclusion Criteria 21
10.3 Patient Trial ID and Treatment Assignment 22
10.4 Method of Blinding 22
10.5 Participant Withdrawal Criteria 22
11 Trial Treatments 23
11.1 Treatment Summary 23
11.2 Non Investigational Medicinal Products 26
11.3 Concomitant Therapy 26
11.4 Emergency unblinding 26
11.5 Accountability and dispensing 26
12 Procedures and assessments 27
12.1 Participant identification 27
12.2 Consent 27
12.3 Screening evaluation 28
12.4 Baseline Assessments 28
12.5 Trial assessments 28
12.6 Schedule of Assessments 32
12.7 Long-Term Follow-up Assessments 33
12.8 End of Trial Participation 33
12.9 Trial restrictions 33
13 Assessment of Safety 33
13.1 Definitions 33
13.2 Expected Adverse Reactions/Serious Adverse Reactions (AR /SARs) 34
13.3 Expected Adverse Events/Serious Adverse Events (AE/SAE) 34
13.4 Evaluation of adverse events 35
13.5 Reporting serious adverse events 36
13.6 Reporting of Suspected Unexpected Serious Adverse Reactions (SUSARs) 36
13.7 Pregnancy Reporting 38
14 Toxicity/Reaction Management 38
15 Evaluation of Results (Definitions and response/evaluation of outcome measures) 38

For peer review only - http://bmjopen.bmj.com/site/about/guidelines.xhtml

15.1 Response criteria 38
16 Storage and Analysis of Samples 38
17 Statistics 38
17.1 Statistical methods 38
17.2 Interim analyses and criteria for the premature termination of the trial 40
17.3 Number of Participants to be enrolled 41
17.4 Procedure to account for missing or spurious data 42
17.5 Definition of the end of the trial 42
18 Data handling and record keeping 42
18.1 CRF 42
18.2 Source Data 43
18.3 Data Protection & Participant Confidentiality 43
19 Data Monitoring Committee/Trial Steering Committee 43
20 Ethical & Regulatory considerations 44
20.1 Ethical committee review 44
20.2 Regulatory Compliance 44
20.3 Protocol Amendments 44
20.4 Peer Review 44
20.5 Public and Patient Involvement 45
20.6 Declaration of Helsinki and Good Clinical Practice 45
20.7 GCP Training 45
21 Sponsorship, Financial and Insurance 45
22 Monitoring, Audit & Inspection 46
23 Protocol Compliance and Breaches of GCP 46
24 Publications policy 46
25 Appendices 47
25.1 Appendix 1 – Authorisation of Participating Sites 47
25.2 Appendix 2 – Safety Reporting Flow Chart 49
25.3 Appendix 3 – Amendment History 50
25.4 Appendix 4 – EQ-5D and CUCQ-32 Questionnaires 50
25.5 Appendix 4 – Treatment decision tree for IASO trial 56
26 REFERENCES 57

For peer review only

For peer review only - http://bmjopen.bmj.com/site/about/guidelines.xhtml

4 Abbreviations

AE	Adverse Event
APR	Annual Progress Report
AR	Adverse Reaction
ARR	Absolute Risk Reduction
ASUC	Acute Severe Ulcerative Colitis
BD	Twice Daily
β-hCG	β-Human Chorionic Gonadotropin
BSG	British Society of Gastroenterology
BSG IBD CRG	BSG IBD Clinical Research Group
CAPS	Cryopyrin-Associated Periodic Syndromes
CCTU	Cambridge Clinical Trials Unit
CCUK	Crohn's & Colitis UK
CI	Chief Investigator
CMV	Cytomegalovirus
CONSORT	Consolidated Standards of Reporting Trials
CRF	Case Report Form
CRP	C-Reactive Protein
CTA	Clinical Trial Authorisation
CTIMP	Clinical Trial of Investigational Medicinal Product
CUCQ-32	Crohn's and ulcerative colitis questionnaire-32
CYP450	Cytochrome p450
DIBD	Developmental International Birth Date
DSUR	Development Safety Update Report
eGFR	Estimated Glomerular Filtration Rate
EME	Efficacy and Mechanism Evaluation
EQ-5D	EuroQol five dimensions
EudraCT	European Clinical Trials Database
GCP	Good Clinical Practice
GI	Gastrointestinal
GMP	Good Manufacturing Practice
HES	Hospital Episode Statistics
HRA	Health Research Authority
IB	Investigator Brochure
IBD	Inflammatory Bowel Disease
IL-1	Interleukin-1 (including A and B variants)
IL-1R	Interleukin-1 Receptor
IL-1RA	Interleukin-1 Receptor Antagonist
IL-6	Interleukin-6
IL-10	Interleukin-10
iDMC	Independent Data Monitoring Committee
IMP	Investigational Medicinal Product
ISF	Investigator Site File
ISRCTN Trials	International Standard Randomised Controlled Number
IV	Intravenous
MAR	Missing-at-random
MHRA	Medicines and Healthcare products Regulatory Agency
MRC	Medical Research Council
mRNA	Messenger Ribonucleic Acid
MTWSI	Modified Truelove and Witts Severity Index

For peer review only

For peer review only - http://bmjopen.bmj.com/site/about/guidelines.xhtml

NHS R&D	National Health Service Research & Development
NIHR	National Institute for Health Research
NIMP	Non-Investigational Medicinal Product
PI	Principal Investigator
PK	Pharmacokinetic
RA	Rheumatoid Arthiritis
RSI	Reference Safety Information
R&D	Research & Development
RCT	Randomised Control Trial
REC	Research Ethics Committee
SAE	Serious Adverse Event
SAR	Serious Adverse Reaction
SC	Subcutaneous
SOP	Standard Operating Procedure
SmPC	Summary of Product Characteristics
SUSAR	Suspected Unexpected Serious Adverse Reaction
TB	Tuberculosis
TMG	Trial Management Group
TSC	Trial Steering Committee
TMF	Trial Master File
TNF-α	Tumour Necrosis Factor α
UC	Ulcerative Colitis
UK	United Kingdom

5 Trial Synopsis

Title of clinical trial	A phase II randomised placebo controlled double blinded trial of Interleukin 1 blockade in Acute Severe Colitis
Sponsor name	Cambridge University Hospitals NHS Foundation Trust and the University of Cambridge
EudraCT number	2017-001389-10
Medical condition or disease under investigation	Acute severe ulcerative colitis
Purpose of clinical trial	To evaluate whether anakinra is an effective treatment for patients with acute severe ulcerative colitis
Primary objective	To compare the clinical effects of anakinra with placebo when given in addition to current standard care in patients with acute severe colitis.
Secondary objective (s)	To compare the effects of anakinra with placebo when given in addition to current standard care to patients with acute severe colitis in terms of:<br>• Safety<br>• Need for colectomy<br>• Patient reported outcomes<br>• Endoscopic and histologic evidence of treatment effects<br>• Evidence of disruption of the IL-1 signalling pathway

For peer review only - http://bmjopen.bmj.com/site/about/guidelines.xhtml

Trial Design	Phase II, Randomised, placebo controlled, double blind
Primary Outcome Measure	Need for medical or surgical rescue therapy within 10 days following commencement of intravenous corticosteroid therapy.
Secondary Outcome Measures	• Need for colectomy within 98 days following commencement of intravenous corticosteroid therapy<br>• Disease activity<br>• Time to clinical response<br>• Time to rescue therapy (medical or surgical)<br>• Incidence of serious adverse events<br><br>Exploratory Outcome Measures<br><br>• Patient-reported quality of life (EQ-5D[25] and CUCQ-32[26]) at baseline and at 3 and 6 months following commencement of IMP treatment<br>• Centrally- and locally-scored endoscopic response at Day 3 (± 1 day) following commencement of IMP treatment (endoscopic sub-study group only)
Planned Sample Size	214 patients (subject to satisfactory progress through feasibility and interim analyses)<br>40 patients will participate in the endoscopic sub-study
Summary of eligibility criteria	Inclusion Criteria:<br>• Patients aged 16–80 inclusive<br>• Hospitalised patients with clinically confirmed or suspected ASUC and a MTWSI score ≥11<br>• Requirement for treatment with IV corticosteroids in the judgement of the treating clinician, with the possibility to receive a first dose of IMP within 36 hours of commencement of IV corticosteroids<br>Exclusion Criteria<br>• Pregnant or breast-feeding women<br>• Oral corticosteroid dosing for a duration of 8 weeks or more immediately prior to commencement of IV corticosteroid dosing<br>• History of severe hepatic impairment (e.g. Child-Pugh = Grade C)<br>• Moderate or severe renal impairment (eGFR <60ml/min/1.73m^2)<br>• Neutropenia (neutrophil count <1.5x10^9/l)<br>• Previous treatment with anakinra for any indication<br>• Documented hypersensitivity to the active substance or to any of the excipients or to

For peer review only - http://bmjopen.bmj.com/site/about/guidelines.xhtml

	E. coli derived proteins; latex allergy<br>• Evidence (from blood cultures etc) or clinical suspicion of systemic infection*<br>• Current or previous cytomegalovirus (CMV) infection requiring treatment with anti-viral agents<br>• Current treatment with anti-TNF-α therapy or anti-TNF-α discontinuation within previous 16 weeks<br>• A history of pulmonary TB infection<br>• Any absolute contraindication to IV corticosteroid<br>• History of malignancy (with the exception of non-melanoma skin cancer) or colonic dysplasia<br>• Rectal therapy in previous 14 days (sub-study exclusion only)<br>• Receipt of another IMP as part of a CTIMP within the previous 16 weeks<br>*concurrent prescription of antibiotics to cover the possibility of GI infection whilst awaiting stool culture results, or the possibility of bacterial translocation relating to severe colitis, is not an exclusion criterion where the physician suspects ulcerative colitis is the most likely diagnosis.
Investigational medicinal product and dosage	100 mg Anakinra and matched placebo<br><br>Single 100mg intravenous loading dose followed by 100mg subcutaneous dose twice daily (up to 10 doses)
NIMPs	IV methylprednisolone or hydrocortisone, as per local guidelines for ASUC standard care.
Route(s) of administration	Intravenous and subcutaneous
Maximum duration of treatment of a participant	5 days (1 IV dose, 10 SC doses)
Procedures: Screening & enrolment	Identification of potential trial participants will be performed by the clinical care team. Consenting and screening of potential participants will be performed at each participating site by trial investigators, or by a suitably qualified and delegated member of the trial team.<br><br>A total of 40 participants recruited at 4 centres will be asked to join an optional scientific sub-study cohort. Participants can decline to participate in the sub-study but still participate in the main study.<br><br>Screening will be performed based upon the eligibility criteria and will involve:<br>• Review of the inclusion/exclusion criteria

For peer review only - http://bmjopen.bmj.com/site/about/guidelines.xhtml

	• Urine β-hCG, if necessary
Procedures: Baseline	The following activities will be performed and recorded at baseline:<br>• Medical history review<br>• Review of routine blood parameters<br>• 3 research blood samples (1 plasma tube, 1 nucleic acid tube, 1 genotyping tube)<br>• Concomitant medications review<br>• Demographics<br>• MTWSI Assessment / review of standard care MTWSI assessments<br>• Stool sample collection<br>• EQ-5D and CUCQ-32 questionnaires
Procedures: Treatment period	Administration of IMP as indicated in the dosing schedule.<br><br>Daily activities following initial dosing:<br>• MTWSI Assessment / review of standard care MTWSI assessments<br>• Recording of any changes to concomitant medication prescription since the last assessment<br>• Review of blood parameters following blood tests performed as part of standard care<br>• 2 research blood samples (1 plasma tube, 1 nucleic acid tube)<br>• Review of adverse events<br>• Medical or surgical (emergency colectomy) rescue therapy assessment<br><br>In addition to the daily data collection described above for Days 1-5, a stool sample will be collected on Day 5 (± 1 day).<br><br>As part of the sub-study participants will be undergo an additional flexible sigmoidoscopy (without bowel preparation) and biopsy sampling on Day 3 (± 1 day) post commencement of IMP treatment.
Procedures: Follow Up period	Following the participants' inpatient stay, unless specified otherwise (See Section 12.5), assessments will occur at the following timepoints:<br>• Day 10 (+3) – Rescue therapy assessment & AE review<br>• Day 98 (+ 14) – colectomy assessment<br>• Approximately 3 and 6 months following commencement of IMP treatment – participants will be contacted by post and asked to complete the EQ-5D and CUCQ-32 questionnaires.

For peer review only - http://bmjopen.bmj.com/site/about/guidelines.xhtml

Procedures: End of trial	Participant status during following discharge, including readmissions and subsequent surgery may be assessed using patient-specific HES and equivalent data from NHS Digital. Data collection would start at 1 year post-commencement of IMP treatment and possible examination of outcomes via HES data would potentially continue for up to 5 years, depending on study outcomes.
Procedures for safety monitoring during trial	The DMC and TSC will periodically review safety data during the trial.
Criteria for withdrawal of participants	<u>Treatment termination criteria</u><br><br>Participants may withdraw from treatment or the full study at any time without prejudice to their clinical care. In addition, responsible clinicians should cease treatment with the study drug according to their judgement or under any of the following criteria:<br>• Development of renal impairment (eGFR rate <60 ml/min/1.73m^2) or severe hepatic impairment (Child Pugh grade C)<br>• Development of neutropenia (neutrophil count <1.5x10^9/l)<br>• Detection of a significant GI pathogen in stool specimen<br>• Sufficient recovery to allow for cessation of IV steroids<br>• Hospital discharge due to recovery<br>• Decision to commence medical or surgical rescue therapy<br>• CMV reactivation necessitating treatment with anti-viral agents (in the judgement of treating clinicians)<br>• The development of a SAE necessitating termination of IMP treatment.<br><br><u>Trial withdrawal criteria</u><br>In addition to the treatment termination criteria, the following criteria would result in participant withdrawal from the trial, with no further assessments being performed:<br>• Withdrawal of consent to collect data<br>• Death

For peer review only - http://bmjopen.bmj.com/site/about/guidelines.xhtml

6 Trial Flow Chart

UNIVERSITY OF CAMBRIDGE

Cambridge University Hospitals NHS
NHS Foundation Trust

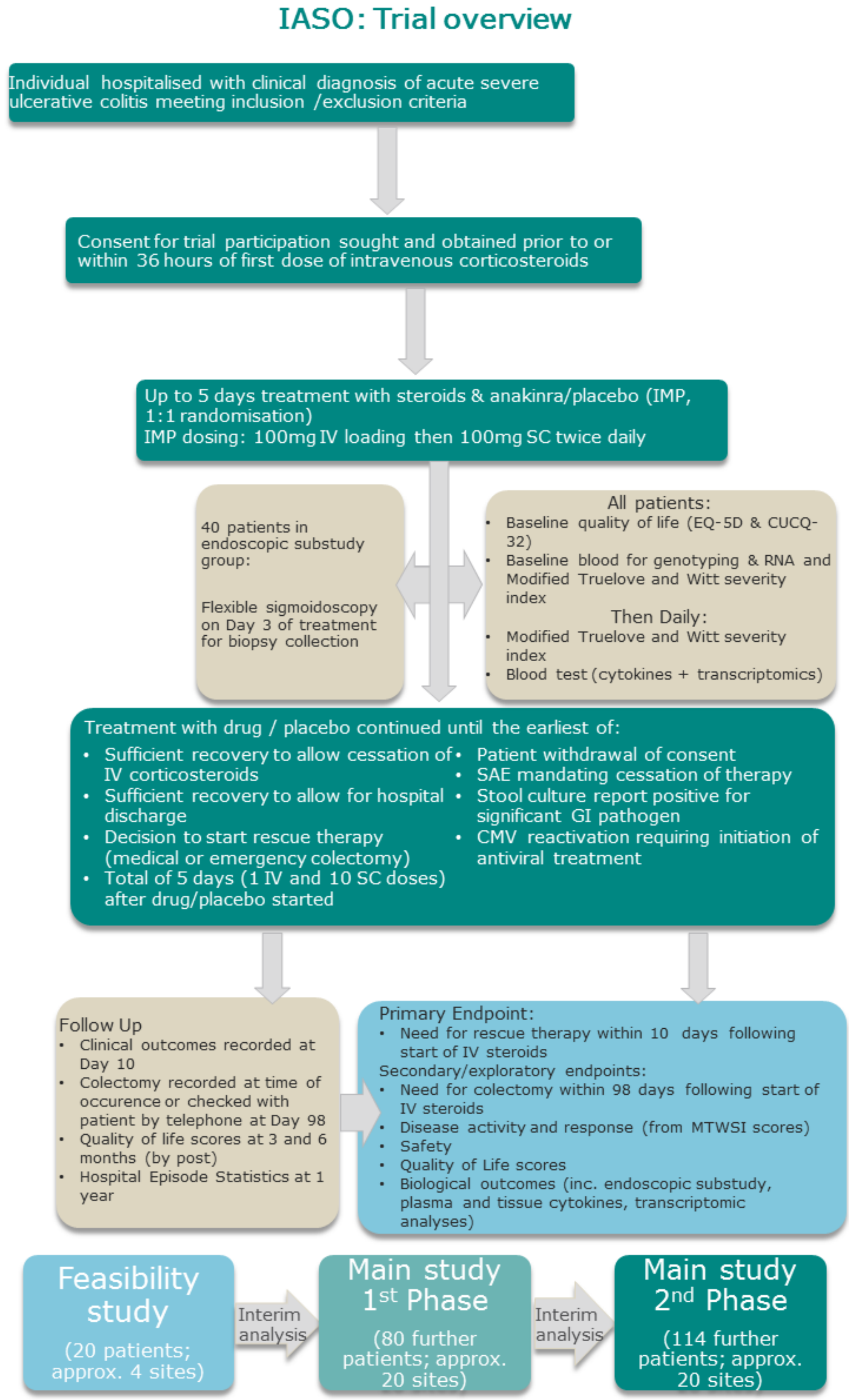

For peer review only - http://bmjopen.bmj.com/site/about/guidelines.xhtml

7 Introduction

7.1 Background

Acute severe ulcerative colitis carries a burden of morbidity and mortality with limited treatment options

Ulcerative colitis (UC) is an inflammatory condition of the colon associated with diarrhoea, rectal bleeding and abdominal pain. UC affects around 120,000 people in the UK. UC tends to follow a relapsing/remitting course with periods of comparative health interspersed with unpredictable acute 'flares'. These may be severe enough to mandate hospital admission: Annual UK hospitalisation rates with acute severe UC (ASUC) are around 3% (3600 individuals) per annum, with 20-40% of patients requiring emergency surgical removal of the colon (colectomy) during the same hospital admission. A recent UK audit of emergency surgery in colitis showed significant mortality rates[1]. Additionally, surgery carries significant morbidity associated with the need for at least a temporary external drain for the faecal material through the skin (stoma), as well as long-term physical and psychological complications.

The care of patients with ASUC follows standardised pathways throughout Europe and much of the rest of the world[2]. Patients meeting diagnostic criteria for ASUC are started on high dose intravenous (IV) corticosteroids. After 3-5 days, patients who are not meeting criteria for improvement may be considered for 'rescue' therapy with further immunosuppression using either infliximab or ciclosporin. Infliximab is costly, and although ciclosporin is a cheaper drug, it requires monitoring and is associated with complications that significantly increase the overall cost. Those patients who fail to respond to medical treatment will then proceed to a colectomy usually performed within 30 days of admission, with removal of most of the colon, and creation of a stoma.

Colonic inflammation in ASUC is characterised by neutrophilic infiltration and high levels of interleukin-1

Colonic inflammation in ASUC is marked by infiltration of neutrophils. These express high levels of a range of inflammatory cytokines, including tumour necrosis factor-α (TNF-α, the molecular target of infliximab) and interleukin-1 (IL-1). IL-1 plays a pivotal role in driving neutrophil activation and a number of downstream inflammatory mediators[3]. High levels of IL-1 are found in inflamed colonic tissue from patients with ASUC[4], and the concentration of IL-1 in the inflamed bowel correlates with endoscopic measurements of severity[5, 6]. The IL-1 axis has been repeatedly identified as a therapeutic target in UC, but this hypothesis has yet to lead to a published clinical trial[4, 7, 8]. The receptor for IL-1 is blocked by an antagonist known as anakinra, a recombinant protein similar to the endogenous protein known as IL-1 receptor antagonist (IL-1RA).

7.2 Clinical Data

Anakinra is a recombinant protein that blocks IL-1 receptor signalling

Anakinra is a low cost drug that has been studied in a number of different indications, but there are no published trials of its use in UC. During the 1990s a clinical trials programme in patients with sepsis culminated in phase III studies that found no evidence of benefit, but importantly, no evidence of an adverse safety signal (and importantly, no evidence of worsening of infectious complications) despite the drug being given at high doses to critically ill patients with systemic infections[9]. The drug is currently licensed for use in patients with rheumatoid arthritis following successful clinical studies that again showed a favourable safety profile[10]. More recent studies have used anakinra in a wide range of indications and doses. Anakinra has recently been used successfully to treat 2 patients with inflammation of the colon related to

For peer review only - http://bmjopen.bmj.com/site/about/guidelines.xhtml

chronic granulomatous disease[11], as well as in a number of animal models of colitis[11, 12].

Anakinra has a short half-life when given IV, but can achieve stable plasma concentrations with subcutaneous (SC) dosing[13]. Using a pharmacokinetic (PK) model informed by tissue microdialysis data and serum measurements in patients receiving anakinra in the context of a clinical trial, we have modelled a dosing regimen for anakinra based upon rapid IV loading (100 mg) then 100 mg twice daily SC doses (Figure 1).

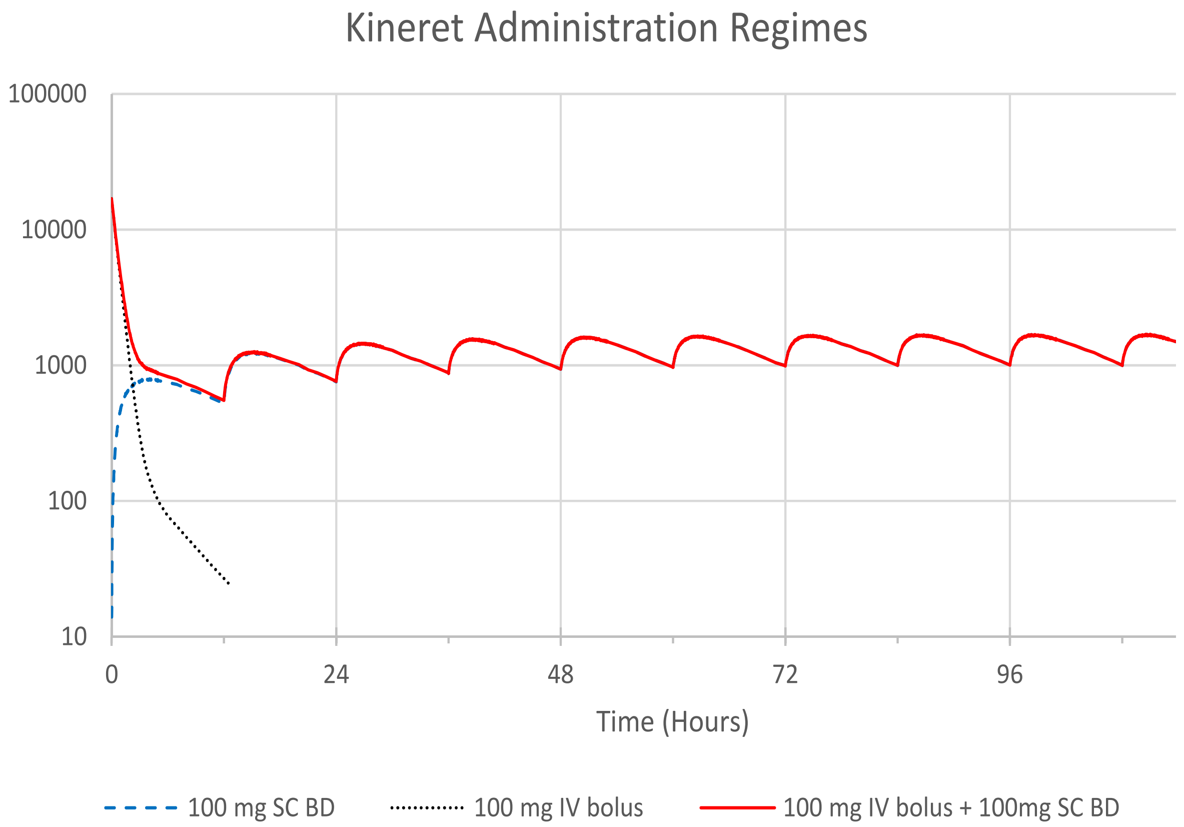

Figure 1. The IASO trial dosing strategy rapidly achieves stable anakinra tissue concentrations in the therapeutic range. (Predicted concentrations of anakinra from simulation data from pharmacokinetic model using trial population data, courtesy Dr J Galea)

We estimate this achieves tissue concentrations of anakinra around 1000ng/ml. Since IL-1 receptor signalling can be stimulated by receptor occupancy levels as low as 2%, large excess concentrations of anakinra are needed to block signalling[14]. Based upon published data demonstrating tissue IL-1 concentrations in UC, our chosen dosing strategy should achieve colonic tissue anakinra concentrations sufficient to antagonise IL-1 signalling and hence reduce or prevent neutrophil activation[5, 15]. This may lead to improvements in patient health at early timepoints following admission.

As part of routine clinical practice for ASUC, treatment with corticosteroids reduces the secretion of both IL-1 and IL-1RA by neutrophils. However, the effect on IL-1RA is greater than on IL-1, skewing the IL-1:IL-1RA ratio towards greater IL-1 availability[16]. This suggests a potential for synergy between corticosteroids and anakinra to reduce total IL-1 levels whilst also rebalancing the IL-1:IL-1RA ratio in favour of the anti-inflammatory IL-1RA.

Monoclonal antibodies are currently in development to target IL-1 signalling. However, in comparison to these, anakinra has a number of advantages:

For peer review only - http://bmjopen.bmj.com/site/about/guidelines.xhtml

- Cessation of drug therapy is followed by rapid elimination of the drug from the plasma over a matter of hours, whereas monoclonal antibodies will take several weeks for elimination increasing the risks in the event of a serious adverse event (SAE) or when giving rescue therapy.
- Anakinra blocks the IL-1 receptor (IL-1R) and therefore antagonises the effects of both forms of biologically active IL-1: IL-1A and IL-1B; anti-IL-1 monoclonal antibodies by their nature must be specific for either IL-1A or IL-1B.
- Finally, anakinra is affordable in contrast to the costs associated with monoclonal antibody development and production.

A trial of anakinra in ASUC is needed now

The initial management of ASUC has changed little since 1955 when, in an early randomised placebo-controlled trial, Truelove and Witts demonstrated that IV corticosteroids reduce the need for colectomy[17]. Despite significant advances in the management of ambulatory patients with UC, patients with ASUC have largely been excluded from clinical studies and the initial therapeutic management of patients after hospitalisation has scarcely changed in 60 years. Major therapeutic advances have been restricted to the development of 'rescue' therapy for those failing treatment with IV corticosteroids. These have been performed in small cohorts (a study of just 20 patients randomised to placebo or IV ciclosporin and a study of 84 patients randomised to infliximab or placebo[18, 19]). The use of 'rescue' therapies has undoubtedly advanced care and is currently necessary in around 50% of patients ([20] and Prof John Williams personal communication from CONSTRUCT trial data). For those receiving rescue therapy, colectomy rates at 3 months remain around 50% (CONSTRUCT trial data). Avoiding surgery, particularly emergency surgery, and reducing the impact of hospitalisation with ASUC has been identified by patients as a key priority[21].

With current standards of care, there is a difficult balance to be struck between the risks of giving expensive or toxic rescue therapy to those who may not need it, versus the risks of delaying effective medical treatment for those who will fail corticosteroid therapy alone. The question therefore arises as to whether we can improve the management of ASUC by doing something different right from the point of initial treatment, rather than waiting for treatment failure? The ideal therapy to test in this regard would be safe, cheap and potentially efficacious. To reduce risk during trials, the therapy should also be capable of being given alongside or even potentially synergistic with steroid therapy. In all these regards, anakinra shows strong potential.

8 Rationale for Trial

A randomised, placebo controlled trial of anakinra in patients with ASUC offers an opportunity to understand whether this drug can benefit patients by impacting upon outcomes in a clinically meaningful manner. Although no formal health economic analysis is planned given the scale and limitations of the trial, the low cost of the trial intervention, and the high cost of the outcomes we are trying to prevent (rescue therapy, prolonged admission, surgery) mean that there is also a strong possibility for potential health economic benefits to arise from a successful trial outcome.

Although anakinra has never been used previously in ASUC, we have adopted an approach of proceeding to an RCT with due consideration of alternatives and with some important safety measures. An alternative would have been to administer the drug on an open-label basis in the first instance. However, interpretation of open-label data without a matched placebo group would be problematic in a condition showing heterogeneity in outcomes between reported studies[22]. Furthermore, anakinra already has a proven safety record when given to large numbers patients, including those on immunosuppressants and with systemic infections. Finally, any patients given

For peer review only - http://bmjopen.bmj.com/site/about/guidelines.xhtml

open-label anakinra would not be able to contribute data to a subsequent RCT due to the absence of blinding. Instead, this trial proceeds directly to a placebo controlled RCT with a planned series of early analyses that will test trial feasibility and detect any evidence of futility (see details below). This allows all patients to contribute data to the final analysis, whilst reducing trial duration, costs and risks to patients.

This trial will involve exposure to anakinra at a dose considerably lower than that used in large scale phase III trials in sepsis (up to 10mg/kg/hr intravenous – i.e. 16.8g/day for a 70kg patient vs the 200mg/day dose in this trial). Despite being performed in critically ill patients, including those with systemic infections, these studies reported no significant adverse safety profile. It should be noted that the licensed route of administration of anakinra is by daily SC injection, whilst in this trial we will use a loading dose given as an IV bolus, followed by twice daily SC injections. Nevertheless, in addition to the much higher IV doses used in the previous phase III sepsis studies mentioned, anakinra has also been used at a range of concentrations given by both intravenous and subcutaneous routes in a number of other trials with a favourable safety profile[9, 10], including inpatient studies adopting a similar twice daily SC injection schedule[23].

In accordance with standard Clinical Trial of Investigational Medicinal Product (CTIMP) design, staff at participating sites will be Good Clinical Practice (GCP) trained, receive trial specific training and be subject to GCP inspections. Day-to-day trial management will be supported by the Trial Management Group (TMG). The trial will be overseen by trial steering committee (TSC) supported by an independent data monitoring committee (iDMC). Due to the potential for a combined immunosuppressant effect of treatment with corticosteroids, anakinra and then rescue therapy, the iDMC will be specifically asked to monitor for infection-related SAEs.

Anakinra will be used in addition to the current standard of care. No currently effective treatments will be withheld or delayed.

Despite the robust scientific rationale and supporting animal data, there are no published reports of the use of anakinra in ASUC. Therefore, the study design will include a series of interim analyses performed in an unblinded manner by the data monitoring committee which will be designed to detect any evidence of (a) harm and (b) futility.

The first interim analysis, designed to assess any major safety signals, will be performed after the first 20 participants have been recruited (feasibility study). A further interim analysis is scheduled after the first 100 participants have been recruited, and is designed to assess for both safety and any evidence of futility. The independent data monitoring committee will be asked to assess the likelihood of the trial producing a negative outcome, and will advise the TSC on the need for early trial termination.

9 Trial Design

9.1 Statement of Design

This phase II trial comprises a two-arm (parallel group), randomised, placebo-controlled, multi-centre, double-blinded study with pre-specified interim analysis for evidence of harm or futility.

A sub-study will also investigate endoscopic response to treatment in both trial arms.

For peer review only - http://bmjopen.bmj.com/site/about/guidelines.xhtml

9.2 Number of Centres

IASO will be a multi-centre study based in UK NHS acute hospitals.
We plan to include approximately 20 centres in the trial.

All sites will recruit, treat and follow up participants equally, but a subset of sites will also recruit participants into the optional endoscopic sub-study involving one additional flexible sigmoidoscopy with biopsy collection.

9.3 Number of Participants

We plan to include approximately 214 participants in the trial with 40 participants also included in the optional endoscopic sub-study.

Further details regarding the sample size justifications can be found in Section 17.3.1.

9.4 Participants Trial Duration

Participants' involvement in the trial will last approximately 6 months. This includes an initial treatment period of approximately 5–7 days, during which screening and an investigational medicinal product treatment course of up to 5 days will be completed. After this, there will be a follow up period of approximately 6 months.

Review of participant outcomes following trial participation may continue for up to 5 years using Hospital Episode Statistics (HES) data, without any additional burden to the participant.

9.5 Trial Objectives

IASO is a trial to evaluate whether anakinra is an effective treatment for patients with ASUC. The trial will test whether anakinra can reduce the need for medical or surgical rescue therapy and improve outcomes including colectomy in patients with ASUC when given alongside corticosteroids. The primary aim is to compare the clinical effects of anakinra with placebo when given in addition to current standard care. The secondary aims include assessing the effects of the intervention on disease biology and bowel inflammation, as well as drug safety, and effects on longer term health and patient reported quality of life. The trial also aims to generate scientific data to permit analysis of plasma and tissue markers of response (both to anakinra and to steroids), in particular showing the effects of IL-1 blockade on the mucosal immune system. The trial will seek to validate a transcriptional predictor of outcomes in UC that we have previously reported[24].

9.5.1 Primary objective

The primary objective of this trial is to compare the clinical effects of anakinra with placebo when given in addition to current standard care in patients with acute severe colitis.

9.5.2 Secondary objectives

To compare the effects of anakinra with placebo when given in addition to current standard care to patients with acute severe colitis in terms of:

- Safety
- Need for colectomy
- Patient reported outcomes
- Endoscopic and histologic evidence of treatment effects
- Evidence of disruption of the IL-1 signalling pathway

For peer review only - http://bmjopen.bmj.com/site/about/guidelines.xhtml

9.6 Trial Outcome Measures

9.6.1 Primary outcome measure

Incidence of medical or surgical rescue therapy within 10 days following the commencement of intravenous corticosteroid therapy.

Need for rescue therapy reflects failure to respond to initial treatment and is judged against clinical criteria between 3-5 days after commencement of IV corticosteroid therapy[2] (see Appendix 5 – Treatment decision tree for IASO trial). In line with international guidelines, the initiation of rescue therapy should not be delayed in those with clinical need. A reduction in the need for rescue therapy will reflect evidence of beneficial effects of the intervention. Although rescue therapy may be initiated as early as 3 days following commencement of intravenous corticosteroids, some clinicians may choose to delay this decision to allow more time for a clinical response. The outcome measure will therefore be the proportion of participants in each group who have started rescue therapy within 10 days following the commencement of intravenous corticosteroids.

For the purposes of the primary endpoint analysis, the timepoint of the start of rescue therapy will be as follows:

- Medical rescue therapy: Date and approximate time of first treatment administration
- Surgical rescue therapy: Date and approximate time of start of surgery

9.6.2 Secondary outcome measures

1. *Incidence of Colectomy within 98 days following commencement of IV corticosteroid therapy.*

2. *Burden of disease activity, measured by daily modified Truelove and Witts severity index (MTWSI)[18] scores over Days 1-5 after initial IMP administration.*

3. *Time to clinical response (defined as 2nd consecutive day with MTWSI<10)*

4. *Time to medical or surgical rescue therapy, measured according to the time after the first dose of IV corticosteroids until the time that rescue therapy occurs (using definitions as set out in primary endpoint). Data will be captured up to the same timepoint as the primary endpoint.*

5. *Incidence of SAEs, measured until Day 10 (+3) following* commencement of IMP treatment.

9.6.3 Exploratory outcome measure

1. Patient-reported quality of life (EQ-5D[25] and CUCQ-32[26]) at baseline and at 3 and 6 months following commencement of IMP treatment
2. Endoscopic response at Day 3 (± 1 day) (via standardised endoscopic scoring completed by a local and a central blinded assessor) following commencement of IMP treatment (endoscopic sub-study group only)

In addition to the above exploratory endpoints, the trial will also aim to collect data to perform further exploratory analyses. These will include data related to investigating

For peer review only - http://bmjopen.bmj.com/site/about/guidelines.xhtml

biomarkers of ASUC (gene and inflammatory cytokine expression), predictors of response (based on IL-1 signalling as well as biochemical and haematological parameters [e.g. haemoglobin, total white blood cell, neutrophil, lymphocyte, monocyte, platelet, albumin and c-reactive protein levels]) and stool microbial characterisation at baseline and at Day 5 (± 1 day).

Furthermore, data from the endoscopic substudy will be investigated to determine the histological response to treatment in addition to determining the expression of inflammatory mediators (e.g. tissue IL-1A, IL-1B, IL-1RA, TNF-α, IL-6, IL-10 levels) at Day 3 (± 1 day) following commencement of IMP treatment. Finally, patient readmission and colectomy data (e.g. via HES) may be examined to investigate the impact of treatment in the longer term.

10 Selection and withdrawal of participants

10.1 Inclusion Criteria

To be included in the trial the participant must:

- Be aged 16–80 years inclusive
- Have given written informed consent to participate
- Be hospitalised with clinically confirmed or suspected ASUC and a MTWSI score ≥11
- Have a requirement for treatment with IV corticosteroids in the judgement of the treating clinician, with the possibility to receive a first dose of IMP within 36 hours of commencement of IV corticosteroids

10.2 Exclusion Criteria

The presence of any of the following will preclude participant inclusion:

- Pregnant or breast-feeding women
- Oral corticosteroid dosing for a duration of 8 weeks or more immediately prior to commencement of IV corticosteroid dosing
- History of severe hepatic impairment (e.g. Child-Pugh = Grade C)
- Moderate or Severe renal impairment (estimated glomerular filtration rate [eGFR] <60ml/min/1.73m^2)
- Neutropenia (neutrophil count <1.5x10^9/l)
- Previous treatment with anakinra for any indication
- Documented hypersensitivity to the active substance or to any of the excipients or to E. coli derived proteins; latex allergy
- Evidence (from blood cultures etc) or clinical suspicion of systemic infection*
- Current or previous cytomegalovirus (CMV) infection requiring treatment with anti-viral agents
- Current treatment with anti-TNF-α therapy or anti-TNF-α discontinuation within previous 16 weeks
- A history of pulmonary TB infection
- Any absolute contraindication to IV corticosteroid
- History of malignancy (with the exception of non-melanoma skin cancer) or colonic dysplasia
- Rectal therapy in previous 14 days (sub-study exclusion only)
- Receipt of another IMP as part of a CTIMP within the previous 16 weeks

*concurrent prescription of antibiotics to cover for the possibility of GI infection whilst awaiting stool culture results, or the possibility of bacterial translocation relating to severe colitis, is not an exclusion criterion where the physician suspects ulcerative colitis is the most likely diagnosis.

For peer review only - http://bmjopen.bmj.com/site/about/guidelines.xhtml

10.3 Patient Trial ID and Treatment Assignment

All patients screened for the trial will be assigned a unique participant ID number. Suitable participants will subsequently be randomised (1:1) to drug:placebo using an online randomisation system accessible via password-protected access. Randomisation will be stratified for:

- Previous or current therapy with any of: immunomodulators (azathioprine, 6-mercaptopurine, 6-thioguanine, methotrexate, ciclosporin) biologics (anti-TNF-α monoclonal antibodies, anti-adhesion molecule antibodies, other anti-cytokine antibodies) or oral janus kinase inhibitors
- Current or previous oral corticosteroid prescription(s) within 8 weeks prior to first dose of intravenous corticosteroids

Access to the web-based randomisation system (Sealed Envelope) will be via individual user accounts provided to the principal investigator (PI) and suitably trained and delegated members of the research team at each site as appropriate. Immediate allocation of treatment will be performed, with documentation of the decision in a confirmatory email. The system will allocate the participant a treatment code which will relate uniquely to a supply of IMP (complete pack for up to 5 days of treatment) that is held at that trial site.

10.4 Method of Blinding

Trial participants and research teams will be blinded to the treatment group for the duration of the trial. Site pharmacies will also be blinded. The physical appearance of the IMPs is matched and the IMPs will be presented in identical packaging. SC administration of the trial drug has been associated with injection site reactions. The incidence of reactions is reduced by pre-warming the pre-filled syringe prior to injection by holding the syringe in the hand, which will form part of the administration instructions. It is important to note that injection site reactions also occur with the placebo, since, in part, the reaction relates to the carrier buffer[27]. This greatly reduces the risk of accidental unblinding.

10.5 Participant Withdrawal Criteria

There are two types of withdrawal in this trial:

- Treatment termination – participant withdraws from IMP administration
- Trial Withdrawal – participant withdraws from the trial in its entirety

Participants may choose to withdraw from treatment or the full study at any stage without prejudice to standard clinical care.

10.5.1 Treatment termination

Clinicians should cease treatment with the study drug according to their judgement or under any of the following criteria:

- Withdrawal of consent for treatment administration
- Development of renal impairment (eGFR <60 ml/min/1.73m^2) or severe hepatic impairment (Child Pugh grade C)
- Development of neutropenia (neutrophil count <1.5x10^9/l)
- Detection of a significant GI pathogen in stool specimen
- Sufficient recovery to allow for cessation of IV steroids
- Hospital discharge due to recovery
- Decision to commence medical or surgical rescue therapy
- CMV reactivation necessitating treatment with anti-viral agents (in the judgement of treating clinicians)
- The development of a SAE necessitating termination of IMP treatment.

For peer review only - http://bmjopen.bmj.com/site/about/guidelines.xhtml

Where possible, trial assessments will continue following treatment termination, with the exception of the withdrawal conditions described in Section 10.5.2.

Participants who undergo sufficient recovery to be discharged from hospital will not be assessed daily but will be followed up as normal. Participants who undergo a colectomy during their inpatient stay will undergo assessments as planned, with the exception of the MTWSI score.

10.5.2 Trial withdrawal

In addition to the treatment termination criteria, the following criteria would result in participant withdrawal from the trial, with no further assessments being performed:

- Withdrawal of consent for further assessments and data collection
- Death

All participants who received at least one dose of IMP and are withdrawn from the trial will have their outcomes reported as part of the main findings.

Due to the short nature of the intervention, withdrawal rates are expected to be low.

The iDMC will regularly review all safety data and consider whether there is evidence of harm that might necessitate recommending changes to the trial protocol or trial termination. In addition, there will be 2 pre-specified analyses during the trial. The iDMC will consider performance of the trial against pre-specified criteria, and whether to recommend trial termination.

11 Trial Treatments

11.1 Treatment Summary

For the purpose of this trial, the following are considered the IMP(s):

- Anakinra solution for injection
- Placebo to match anakinra solution for injection

11.1.1 Name and description of the IMP

Anakinra (brand name Kineret) is a clear, colourless to white solution for injection which may contain some translucent-to-white particles of protein in the solution. The placebo solution will have the same physical appearance. Both active and placebo treatments will be supplied in commercial pre-filled syringe configurations (100mg/0.67ml), packed into trial-specific participant kits, each containing 11 pre-filled syringes. The syringes and kits will have blinded labelling conforming to regulatory requirements and be identified by unique pack (kit) numbers.

11.1.1.1 *Legal status*

Anakinra is a licensed drug in the UK for the treatment of rheumatoid arthritis and several other indications under the Cryopyrin-Associated Periodic Syndromes (CAPS) grouping and is administered subcutaneously. Within this study, the IMP is being used in a new indication (ASUC), dosage regimen and for the loading dose, administered by a currently unlicensed route (IV bolus dose).

The trial is being carried out under a Clinical Trial Authorisation. The drug is therefore only to be used by the named investigators, for the participants specified in this protocol, and within the trial.

For peer review only - http://bmjopen.bmj.com/site/about/guidelines.xhtml

11.1.1.2 *Supply*

IMP supply to sites will be overseen by the trial co-ordinator and distributed by the Royal Free Hospital, London. Upon initial authorisation by the sponsor, an initial supply will be sent to sites; supplies thereafter will be requested and distributed as detailed in the pharmacy manual. IMP will be distributed and stored as per requirements of the SmPC for Anakinra.

IMP for the trial will be provided free-of-charge for participating sites.

11.1.1.3 *Packing and Labelling*

IMP will be ordered using trial-specific prescriptions stating the kit number to dispense. Participant-specific details will be added to labelling at point of dispensing.

11.1.1.4 *Storage conditions*

IMP will be stored in accordance with the requirements of the SmPC for Anakinra.

11.1.1.5 *Maximum duration of treatment of a participant*

The maximum amount of time a participant will be receiving the IMP will be 5 days (a total of 1 IV and 10 SC doses).

11.1.1.6 *Dose*

Initial IV dose – 100mg
Subsequent SC dose – 100mg for up to 10 doses as per administration schedule in Section 11.1.1.7.

11.1.1.7 *Administration*

Day 0

On the first day of trial medication administration (Day 0):

- IV loading dose of 100 mg IMP
- 100 mg IMP SC twice daily (BD), approximately 12 hours apart
  - The first SC dose is given immediately following the IV loading dose
  - In the event that the first dose of IMP occurs after noon on Day 0, only one 100 mg SC dose will be given that day. Full BD dosing will commence the following day and continue as indicated below for Days 1-5.

Administration of the first dose of IMP will only be performed in the following circumstances:

- The time of administration of the first IMP dose falls no later than 36 hours following the time of first dose of IV corticosteroid.
- IV corticosteroid therapy remains ongoing at the time of administration of the first IMP dose (i.e. that a decision to discontinue corticosteroids or transition to oral steroids has not been made after the time of participant consent and before the time of the administration of the first IMP dose).
- A firm decision to start rescue therapy or perform emergency colectomy has not already been taken.

Day 1-5

During the days subsequent to the first dose of trial medication, the following dosing schedule will be followed:

- BD administration of 100 mg IMP SC

For peer review only - http://bmjopen.bmj.com/site/about/guidelines.xhtml

- For those participants who received two 100 mg IMP SC doses on Day 0, no administration will occur on Day 5.
- For those participants who received only one SC dose on Day 0, the administration of the final dose of 100 mg IMP SC will occur on Day 5.

Dosing will continue until the earliest of:

- 5 days (a total of 1 IV and 10 SC doses)
- Fulfilment of the treatment termination criteria specified in Section 10.5.

11.1.1.8 *Known drug reactions*

Known drug reactions for anakinra are summarised in Section 4.8 of the SmPC for anakinra (Kineret) approved by the MHRA for use in this trial.

Interactions between anakinra and other medicinal products have not been investigated in formal studies. In clinical trials, interactions between anakinra and other medicinal products (including nonsteroidal anti-inflammatory medicinal products, corticosteroids, and DMARDs) have not been observed.

Concurrent anakinra and TNF antagonist treatment

In a clinical trial with RA patients receiving background methotrexate, participants treated with anakinra and etanercept were observed to have a higher rate of serious infections (7%) and neutropenia than participants treated with etanercept alone and higher than observed in previous trials where anakinra was used alone. The concurrent use of anakinra with etanercept or any other TNF antagonist is not recommended.

Within the study, current treatment with anti-TNF-ɑ therapy or anti-TNF-ɑ discontinuation within previous 16 weeks is an exclusion criterion. Where rescue therapy with anti-TNF-ɑ therapy is required, anakinra will be discontinued with rapid elimination expected from the pharmacokinetic characteristics outlined in Section 7.2 above.

Cytochrome P450 Substrates

The formation of cytochrome p450 (CYP450) enzymes is suppressed by increased levels of cytokines (e.g., IL-1) during chronic inflammation. Thus, it may be expected that for an IL-1 receptor antagonist, such as anakinra, the formation of CYP450 enzymes could be normalized during treatment. This would be clinically relevant for CYP450 substrates with a narrow therapeutic index (e.g. warfarin and phenytoin). Upon start or end of anakinra treatment in patients on these types of medicinal products, it may be relevant to consider therapeutic monitoring of the effect or concentration of these products and the individual dose of the medicinal product may need to be adjusted.

Participants in IASO will be acutely unwell in an inpatient setting and only treated with anakinra for a short duration; routine therapeutic monitoring would be standard practice for appropriate concomitant drugs.

11.1.1.9 *Dose modifications*

No dosage modifications are permitted. The IMP course may be stopped before all 10 subcutaneous doses have been completed, as described in Section 10.5.

For peer review only - http://bmjopen.bmj.com/site/about/guidelines.xhtml

11.1.1.10 *Procedures for monitoring treatment compliance*

Participants are treated as inpatients with all IMP being administered by healthcare professionals. Compliance with treatment may be assessed by referring to medication administration records and any remaining unused IMP after treatment has been stopped, which should be returned to the site pharmacy for reconciliation.

11.1.1.11 *Placebo/Active comparator products*

Placebo to match anakinra.

11.2 Non Investigational Medicinal Products

IV methylprednisolone or hydrocortisone, as per local guidelines for ASUC standard care. As these form part of standard care, IV corticosteroids will not be provided to participating sites as part of the trial.

The trial pharmacy manual should be consulted for further details regarding nIMP procedures.

11.3 Concomitant Therapy

Participants will continue to be treated with routine concomitant medications for other co-morbidities as appropriate within their current clinical condition, with modifications e.g. Oral to IV switches, or temporary cessation as necessary. Appropriate supportive care may also be prescribed, including fluids, nutritional supplements and other medications as necessary.

11.4 Emergency unblinding

In the event of a valid medical or safety reason (e.g. in the case of an SAR where it is necessary for the investigator or treating health care professional to know which treatment the participant is receiving before the participant can be treated), the responsibility to break the treatment code resides solely with the treating clinician.

The online Sealed Envelope randomisation system will also be used for emergency unblinding. Appropriately trained and delegated site staff will be given the necessary access rights and permissions to access this facility. The name, contact details of the unblinder and reason for unblinding will be recorded within the Sealed Envelope system.

The unblinder will not be shown the treatment allocation on-screen. Instead the allocation will be sent by email or fax and should be printed and retained confidentially within the ISF. An email stating that an unblinding has taken place will be automatically sent to the coordination team for oversight purposes.

Wherever possible, members of the research team should remain blinded.

11.5 Accountability and dispensing

Sites will maintain a full accountability record documenting receipt, storage, dispensing, administration, return of unused IMP and used and unused IMP packaging as appropriate, as specified in the pharmacy manual.

There will no accountability records maintained for nIMPs listed in section 11.2.

For peer review only - http://bmjopen.bmj.com/site/about/guidelines.xhtml

12 Procedures and assessments

12.1 Participant identification

We will recruit inpatients with ASUC. Identification of potential participants will be undertaken by the clinical team. Consenting and screening of potential trial participants will be performed at each participating site by trial investigators, or by a suitably qualified and delegated member of the trial team.

All patients who are interested in learning more about the trial will be provided with the approved patient information sheet and be invited to consent to join the trial as soon as possible after admission. Anonymised data on all participants who are approached will be collated in accordance with Consolidated Standards of Reporting Trials (CONSORT) guidelines.

12.2 Consent

The Informed Consent form must be approved by the REC and must be in compliance with GCP, local regulatory requirements and legal requirements. The investigator or designee must ensure that each trial participant is fully informed about the nature and objectives of the trial and possible risks associated with their participation.

The investigator or designee will receive written informed consent from each participant before any trial-specific activity is performed. The informed consent form used for this trial and any change made during the course of this trial, must be prospectively approved by the REC. The investigator will retain the original of each participant signed informed consent form.

Should a participant require a verbal translation of the trial documentation by a locally approved interpreter/translator, it is the responsibility of the individual investigator to use locally approved translators.

Any new information which becomes available, which might affect the participant's willingness to continue participating in the trial will be communicated to the participant as soon as possible.

12.2.1 Sub-study Consent

A total of 40 individuals recruited at 4 centres will be asked to join an optional scientific sub-study cohort. These patients will be provided with the approved sub-study patient information sheet. As part of this they will be asked to consent to an additional flexible sigmoidoscopy at Day 3 (± 1 day) after the initiation of IMP treatment with biopsy collection. Participants can decline to participate in the sub-study but still participate in the main study.

Biopsies will be stored in pseudoanonymised form in compliance with the terms of the Human Tissue Act (2004) for the duration of the trial. If a participant withdraws consent from the scientific sub-study and requests that their samples do not undergo analysis, then any biopsy material not already analysed at this stage can be destroyed. Patients who initially consent to the sub-study but do not undergo the flexible sigmoidoscopy (e.g. due to withdrawal of consent or hospital discharge prior to the procedure) will be replaced until a total of 40 trial participants have undergone the procedure.

For peer review only - http://bmjopen.bmj.com/site/about/guidelines.xhtml

12.3 Screening evaluation

12.3.1 Screening Assessments

Trial specific assessments will only be conducted after participants have given written informed consent.

Screening will be performed based upon the inclusion/exclusion criteria outlined in Section 10. Eligibility will be confirmed by the trial investigator or suitably qualified and delegated member of the trial team. The tests required for screening all form part of standard clinical care, therefore no additional tests will be performed other than a urine β-human chorionic gonadotropin (β-hCG) test for females where there is a possibility of pregnancy.
The screening process will therefore involve:

- Review of the patient's medical record to confirm eligibility in line with the inclusion/exclusion criteria
- Urine β-hCG, for women of childbearing potential

12.3.2 Participant Randomisation

Participants will be randomised by a suitably trained and delegated member of the research team as described in section 10.3 and the randomisation manual

12.4 Baseline Assessments

The following activities will be performed and recorded at baseline:

- Medical history review
- Review of blood parameters (haemoglobin, total white blood cell, neutrophil, lymphocyte, monocyte, platelet, albumin and c-reactive protein) following a blood test performed as part of standard care
- 3 research blood samples (1 plasma tube, 1 nucleic acid tube, 1 genotyping tube) as described in the laboratory manual
- Concomitant medications review
- Demographics
- MTWSI assessment
- Stool sample collection
- EQ-5D and CUCQ-32 questionnaires (Appendix 4 – EQ-5D and CUCQ-32 Questionnaires)

12.5 Trial assessments

The below procedures will all occur during the inpatient admission. Participants will be managed in line with standard clinical guidelines for ASUC[2], as set out in the trial decision tree (Appendix 5 – Treatment decision tree for IASO trial
).

Wherever possible, research blood samples will be taken at the same time as a standard care blood samples. Safety monitoring in the trial will be conducted according to the steps described in Section 13. It should be noted that both Baseline and, subsequently, Day 0 may occur on the same calendar date.

Day 0

Prior to the first administration of IMP, the following assessments will be performed:

- Review of adverse events
- Recording of any changes to concomitant medication since the last assessment

For peer review only - http://bmjopen.bmj.com/site/about/guidelines.xhtml

- Confirmation that IMP administration can proceed, according to the following criteria:
  - The time of administration of the first IMP dose falls no later than 36 hours following the time of first dose of IV corticosteroid.
  - IV corticosteroid therapy remains ongoing at the time of administration of the first IMP dose (i.e. that a decision to discontinue corticosteroids or transition to oral steroids has not been made after the time of participant consent to participate in the trial and before the time of the administration of the first IMP dose).
  - A firm decision to start rescue therapy or perform emergency colectomy has not already been taken
- IMP administration

Trial assessments will be performed as normal in the event that a participant was randomised but did not receive IMP.

Day 1-5

During the days subsequent to the first dose of trial medication, the following activities will be recorded for each day up to and including Day 5:

- MTWSI assessment / review of standard care clinical MTWSI assessment domains[#]
- Recording of any changes to concomitant medication prescription since the last assessment
- Review of blood parameters (haemoglobin, total white blood cell, neutrophil, lymphocyte, monocyte, platelet, albumin and c-reactive protein) following blood tests performed as part of standard care
- 2 research blood samples (1 plasma tube, 1 nucleic acid tube)*
- Review of adverse events
- Medical or surgical (emergency colectomy) rescue therapy assessment

[#] *This will not be recorded for patients who have undergone colectomy*

**A minimum of 1 sample every 72 hours should be taken. Wherever possible every effort should be made to obtain the samples every day (up to 10 samples).*

Where possible, trial assessments will continue following treatment termination, with the exception of the withdrawal conditions described in Section 10.5.2. In the event of hospital discharge prior to Day 5, the above activities will not be recorded beyond the timepoint of discharge.

Day 3 (sub-study only) ± 1 day

In addition to the daily assessments described above for Days 1-5, a scientific sub-study group of 40 participants will undergo an additional optional flexible sigmoidoscopy (without bowel preparation) on Day 3 (± 1 day). This will be performed during the index inpatient admission.

Findings will be recorded according to a standardised endoscopic scoring system (Mayo) by blinded local and central assessors. Histology will be scored on up to a maximum of

For peer review only - http://bmjopen.bmj.com/site/about/guidelines.xhtml

6 biopsies taken from each of the rectum and sigmoid colon, according to validated scoring systems for colitis severity at the end of the trial [28, 29]' [30].

Where possible, endoscopic scores, text reports and photographic recordings of sigmoidoscopies will be sent to the central trial team for verification. In addition, where available, data collected as part of standard care (non sub-study) endoscopies will be used to supplement trial-specific data. These will be scored by the blinded central assessor.

Day 5

In addition to the daily assessments described above for Days 1-5, a stool sample will also be collected on Day 5 (± 1 day).

Follow Up Period

Following the participants' inpatient stay, the following assessments will occur:

- Day 10 (+3) following commencement of IMP treatment – Rescue therapy assessment*
- Day 10 (+3) following commencement of IMP treatment – AE monitoring, as described in Section 13
- Day 98 (+ 14) following commencement of IMP treatment – colectomy assessment†
- Approximately 3 months following commencement of IMP treatment – Participants will be contacted by post and asked to complete the EQ-5D and CUCQ-32 questionnaires.
- Approximately 6 months following commencement of IMP treatment – EQ-5D and CUCQ-32 questionnaires.

*If a participant proceeds to rescue therapy or colectomy during the admission, this will be recorded in the medical records and the CRF. In the event that a participant is discharged prior to Day 10 without having started rescue therapy, a member of the trial team will contact the participant on (or within 3 days following) Day 10 to confirm that the participant remains out of hospital and has not received rescue therapy elsewhere since discharge. If the participant is not contactable, local hospital records will be used to check for admission.

†If not previously recorded, a member of the trial team will contact the participant on Day 98 (+14) to confirm that the participant remains out of hospital and has not undergone a colectomy since discharge. If the participant is not contactable, local hospital records will be used to check for admission.

Participant compliance is expected to be high and loss-to-follow up minimal.

The 3 & 6 month questionnaires will be sent to the participants from the central research team based in Cambridge by post. If a participant fails to return the questionnaire a member of the local research team will contact the participant by telephone after 4 and 8 weeks. If required, questionnaires can be re-sent by the central research team as appropriate. Any 3-month questionnaires not received within 12 weeks of being sent to a participant will be marked as missing. However, unless

For peer review only - http://bmjopen.bmj.com/site/about/guidelines.xhtml

requested otherwise by the participant, the subsequent 6 month questionnaires will still be sent to the participant for completion.

For peer review only

For peer review only - http://bmjopen.bmj.com/site/about/guidelines.xhtml

12.6 Schedule of Assessments

Assessments	Treatment phase				Follow up phase			
	Screening	Baseline	Day 0	Assessment Days 1 - 5 (1)	Day 10 (+3 days)	Day 98 (+ 14 days)	~3 Months	~6 Months
Informed consent	✓							
Eligibility assessments	✓							
Urine β-hCG*	✓							
Randomisation	✓							
Medical history review		✓						
Socio-demographic data		✓						
Questionnaires (EQ-5D, CUCQ-32)		✓					✓	✓
Review of standard-care blood parameters		✓		✓				
Collection of 3 research blood samples		✓						
Collection of 2 research blood samples				✓				
Daily MTWSI assessment / review of standard care clinical MTWSI assessment domains#		✓		✓				
Stool Sample Collection		✓		✓(2)				
Concomitant medications (full list)		✓						
Concomitant medications (changes since last assessment)			✓	✓				
Review of adverse events			✓	✓	✓			
Confirmation of continued eligibility to receive IMP and that IMP administration falls within 36 hour window			✓					
Rescue therapy (medical or surgical) assessment				✓	✓(5)			
Colectomy assessment				✓		✓(5)		
**Sub-study-Only Assessments**								
Informed consent	✓							
Flexible sigmoidoscopy and ~12x pinch biopsy collection*				✓(3)				
**Administration Schedule**								
IMP administration – 100 mg IV			✓					
IMP administration – 100 mg SC BD			✓	✓(4)				

(1) – Assessments will continue until hospital discharge/withdrawal/death. Treatment will continue until hospital discharge/treatment termination criteria have been met
(2) – Assessment Day 5 only ± 1day
(3) – Assessment Day 3 only ± 1day
(4) – Dosing at Day 5 (representing the 10th BD SC dose) will only occur in the event that a participant received their 1st overall SC dose in the latter part of Day 0.
(5) – If information not already collected.
*for women of childbearing potential,
#MTWSI score will not be calculated for participants that have undergone colectomy

For peer review only - http://bmjopen.bmj.com/site/about/guidelines.xhtml

12.7 Long-Term Follow-up Assessments

Participant status following discharge, including readmissions and subsequent surgery may be assessed using patient-specific HES data from the NHS Digital, Data Access Request Service. Any data collection would start at 1 year post-commencement of IMP treatment and examination of outcomes via HES data would potentially continue for up to 5 years, depending on study outcomes. A yearly assessment of HES outcomes by the TSC would determine whether collection of HES data should continue.

12.8 End of Trial Participation

Participants will return to their normal standard of care as defined by their local physicians, following completion of trial treatment.

12.9 Trial restrictions

Live vaccinations should not be given during the treatment period. Live vaccinations can be given after the end of treatment period as soon as the participant is judged otherwise well enough to receive the vaccine.

Contraception use or abstinence will not be mandated as participants will be hospital in-patients with an acute severe medical condition. The drug is rapidly metabolised and will be fully eliminated from the circulation within 24 hours of drug cessation. The drug is not genotoxic. Therefore no longer term contraceptive measures are required in participants of either sex.

13 Assessment of Safety

13.1 Definitions

13.1.1 Adverse event (AE)

Any untoward medical occurrence in a participant or clinical trial participant administered a medicinal product and which does not necessarily have a causal relationship with this treatment.
An adverse event can therefore be any unfavourable and unintended sign (including an abnormal laboratory finding), symptom, or disease temporally associated with the use of an investigational medicinal product, whether or not considered related to the investigational medicinal product.

Please note: Recording of all adverse events must start from the point of Informed Consent regardless of whether a participant has yet received a medicinal product.

13.1.2 Adverse reaction to an investigational medicinal product (AR)

All untoward and unintended responses to an investigational medicinal product related to any dose administered. All adverse events judged by either the reporting investigator or the sponsor as having a reasonable causal relationship to a medicinal product qualify as adverse reactions. The expression reasonable causal relationship means to convey in general that there is evidence or argument to suggest a causal relationship.

13.1.3 Unexpected adverse reaction

An adverse reaction, the nature, or severity of which is not consistent with the applicable reference safety information (RSI).

When the outcome of the adverse reaction is not consistent with the applicable RSI this adverse reaction should be considered as unexpected.

For peer review only - http://bmjopen.bmj.com/site/about/guidelines.xhtml

The term "severe" is often used to describe the intensity (severity) of a specific event. This is not the same as "serious," which is based on participant /event outcome or action criteria.

13.1.4 Serious adverse event or serious adverse reaction (SAE / SAR)

Any untoward medical occurrence that at any dose:

- Results in death
- Is life-threatening
- Requires hospitalisation or prolongation of existing inpatients´ hospitalisation
- Results in persistent or significant disability or incapacity
- Is a congenital anomaly or birth defect.
- Is an important medical event - Some medical events may jeopardise the participant or may require an intervention to prevent one of the above characteristics/ consequences. Such events (hereinafter referred to as 'important medical events') should also be considered as 'serious'

Life-threatening in the definition of a serious adverse event or serious adverse reaction refers to an event in which the participant was at risk of death at the time of event; it does not refer to an event which hypothetically might have caused death if it were more severe.

13.1.5 Suspected Unexpected Serious Adverse Reaction (SUSAR)

A serious adverse reaction, the nature and severity of which is not consistent with the information set out in the Reference Safety Information

13.1.6 Reference Safety Information (RSI)

A list of medical events that defines which reactions are expected for the IMP within a given trial and thus determining which Serious Adverse Reactions (SARs) require expedited reporting.

For this trial the Reference Safety Information is: Section 4.8 of the SmPC for anakinra (Kineret), updated 31 March 2016 approved by the MHRA for use in this trial

13.2 Expected Adverse Reactions/Serious Adverse Reactions (AR /SARs)

All expected Adverse Reactions are listed in the latest MHRA approved version of the RSI as specified in section 13.1.6. This must be used when making a determination as to the expectedness of the adverse reaction. If the adverse reaction meets the criteria for seriousness, this must be reported as per section 13.5.

13.3 Expected Adverse Events/Serious Adverse Events (AE/SAE)

The following AEs are common and well-recognised side effects associated with administration of the IMP. They are generally not serious in nature and will not be recorded in the AE/AR Log as part of this trial:

- Injection site and mild skin reactions following SC injection of IMP (anakinra or matched placebo)

The following AEs, which may fall under the definition of SAEs as indicated in Section 13.1, are expected to occur commonly as a result of disease progression, rather than trial participation:

- Diarrhoea/worsening diarrhoea
- Abdominal pain/increased abdominal pain
- Rectal pain/worsening rectal pain

For peer review only - http://bmjopen.bmj.com/site/about/guidelines.xhtml

- Development of toxic megacolon
- Development of hypoalbuminaemia

The above signs and symptoms are expected during disease progression necessitating rescue therapy (medical or surgical). As these events are common and expected outcomes, they will be exempt from SAE reporting (as outlined in Section 13.5), however details will be collected in the CRF. In addition, the decision to prescribe medical rescue therapy or perform emergency colectomy will not need SAE reporting. All instances of medical and surgical rescue therapy will still be collected via the trial outcome data and provided to the iDMC for review.

13.4 Evaluation of adverse events

The Sponsor expects that adverse events are recorded from the point of Informed Consent regardless of whether a participant has yet received a medicinal product. Individual adverse events should be evaluated by the investigator or delegate. This includes the evaluation of its seriousness, and any relationship between the investigational medicinal product(s) and/or concomitant therapy and the adverse event (causality).

13.4.1 Assessment of seriousness

Seriousness is assessed against the criteria in section 13.1.4.This defines whether the event is an adverse event, serious adverse event or a serious adverse reaction

13.4.2 Assessment of causality

Definitely: A causal relationship is clinically/biologically certain. **This is therefore an Adverse Reaction**

Probably: A causal relationship is clinically / biologically highly plausible and there is a plausible time sequence between onset of the AE and administration of the investigational medicinal product and there is a reasonable response on withdrawal. **This is therefore an Adverse Reaction.**

Possibly: A causal relationship is clinically / biologically plausible and there is a plausible time sequence between onset of the AE and administration of the investigational medicinal product. **This is therefore an Adverse Reaction.**

Unlikely: A causal relation is improbable and another documented cause of the AE is most plausible. **This is therefore an Adverse Event.**

Unrelated: A causal relationship can be definitely excluded and another documented cause of the AE is most plausible. **This is therefore an Adverse Event.**

Unlikely and Unrelated causalities are considered NOT to be trial drug related
Definitely, Probably and Possibly causalities are considered to be trial drug related

A pre-existing condition must not be recorded as an AE or reported as an SAE unless the condition worsens during the trial and meets the criteria for reporting or recording in the appropriate section of the CRF.

13.4.3 Clinical assessment of severity

Mild: The participant is aware of the event or symptom, but the event or symptom is easily tolerated

Moderate: The participant experiences sufficient discomfort to interfere with or reduce his or her usual level of activity

For peer review only - http://bmjopen.bmj.com/site/about/guidelines.xhtml

Severe: Significant impairment of functioning; the subject is unable to carry out usual activities and / or the participant's life is at risk from the event.

13.4.4 Recording of adverse events

Adverse events and adverse reactions should be recorded in the medical notes and the appropriate section of the CRF. Serious Adverse Events and Serious Adverse Reactions should be reported to the sponsor as detailed in section 13.5.

13.5 Reporting serious adverse events

Each Principal Investigator needs to record all adverse events and report serious adverse events to the Chief Investigator using the trial specific SAE form within 24 hours of their awareness of the event.

The Chief Investigator is responsible for ensuring the assessment of all SAEs for expectedness and relatedness is completed and the onward notification of all SAEs to the Sponsor immediately but not more than 24 hours of first notification. The sponsor has to keep detailed records of all SAEs reported to them by the trial team.

The Chief Investigator is also responsible for prompt reporting of all serious adverse event findings to the MHRA if they could:

- Adversely affect the health of participants
- Impact on the conduct of the trial
- Alter the risk to benefit ratio of the trial
- Alter the competent authority's authorisation to continue the trial in accordance with Directive 2001/20/EC

The completed SAE form can be faxed or emailed. Details of where to report the SAE's can be found on the IASO SAE form and the front cover of the protocol. SAE information will be shared with the Sobi Drug Safety Department.

The IMP has a short half-life (<6h). No cumulative or late effects are anticipated. For this reason, recording of AEs and reporting of SAEs will be actively monitored up to Day 10 (+3 days). Following the end of the active monitoring period, investigators are still required to report and SARs or SUSARs of which they become aware.
Where a participant withdraws consent for further processing of data, this does not preclude the reporting of SARs and SUSARs which are required to continue being reported according to the protocol for regulatory purposes. All participants must be followed-up until SAE, SAR & SUSAR resolution in all instances.

13.6 Reporting of Suspected Unexpected Serious Adverse Reactions (SUSARs)

All suspected adverse reactions related to an investigational medicinal product (the tested IMP and comparators) which occur in the concerned trial, and that are both unexpected and serious (SUSARs) are subject to expedited reporting. Please see section 13.1.6 for the Reference Safety Information to be used in this trial.

13.6.1 Who should report and whom to report to?

The Sponsor delegates the responsibility of notification of SUSARs to the Chief Investigator. The Chief Investigator must report all the relevant safety information previously described, to the:

- Sponsor
- Competent authorities in the concerned member states (e.g. MHRA)
- Ethics Committee in the concerned member states

For peer review only - http://bmjopen.bmj.com/site/about/guidelines.xhtml

The Chief Investigator shall inform all investigators concerned of relevant information about SUSARs that could adversely affect the safety of participants.

13.6.2 When to report?

13.6.2.1 Fatal or life-threatening SUSARs

All parties listed in 13.6.1 must be notified as soon as possible but no later than **7 calendar days** after the trial team and Sponsor has first knowledge of the minimum criteria for expedited reporting.

In each case relevant follow-up information should be sought and a report completed as soon as possible. It should be communicated to all parties within an additional **8 calendar days**.

13.6.2.2 Non-fatal and non-life-threatening SUSARs

All other SUSARs and safety issues must be reported to all parties listed in 13.6.1 as soon as possible but no later than **15 calendar days** after first knowledge of the minimum criteria for expedited reporting. Further relevant follow-up information should be given as soon as possible.

13.6.3 How to report?

13.6.3.1 Minimum criteria for initial expedited reporting of SUSARs

Information on the final description and evaluation of an adverse reaction report may not be available within the required time frames for reporting. For regulatory purposes, initial expedited reports should be submitted within the time limits as soon as the minimum following criteria are met:
a) A suspected investigational medicinal product
b) An identifiable participant (e.g. trial participant code number)
c) An adverse event assessed as serious and unexpected, and for which there is a reasonable suspected causal relationship
d) An identifiable reporting source

and, when available and applicable:
- An unique clinical trial identification (EudraCT number or in case of non-European Community trials the sponsor's trial protocol code number)
- An unique case identification (i.e. sponsor's case identification number)

13.6.3.2 Follow-up reports of SUSARs

In case of incomplete information at the time of initial reporting, all the appropriate information for an adequate analysis of causality should be actively sought from the reporter or other available sources. Further available relevant information should be reported as follow-up reports.
In certain cases, it may be appropriate to conduct follow-up of the long-term outcome of a particular reaction.

13.6.3.3 Format of the SUSARs reports

Electronic reporting is the expected method for expedited reporting of SUSARs to the competent authority. The format and content as defined by the competent authority should be adhered to.

For peer review only - http://bmjopen.bmj.com/site/about/guidelines.xhtml

13.7 Pregnancy Reporting

Pregnancy reporting is only required for trial participants whilst they are receiving IMP and for up to 24 hours post their last dose.

14 Toxicity/Reaction Management

Anaphylaxis: intravenous chlorphenamine 10mg (Piriton), hydrocortisone 200mg, and rarely adrenaline, will be given in line with local procedures for anaphylaxis.

Injection site reactions are relatively common with both IMP and placebo, but can be minimised by warming the syringe before injection. If necessary, application of a cold pack to the injection site 2–3 min before and immediately after the injection can reduce acute pain associated with injections. Paracetamol may also be used to help with pain. Delayed skin reactions can be managed by topical application of 1% hydrocortisone or anti-histamine creams. It is recommended to alternate the injection sites to avoid recall reactions.

15 Evaluation of Results (Definitions and response/evaluation of outcome measures)

MTWSI will be recorded along with other patient reported outcomes and laboratory measures, as detailed in the CRF.

15.1 Response criteria

Treatment response according to MTWSI will be defined as 2 consecutive days with MTWSI <10.

16 Storage and Analysis of Samples

Blood samples will be collected and centrifuged according to the laboratory manual. Following centrifugation, plasma should be apportioned into clean, labelled tubes prior to storage in a -20°C to -80 freezer.

Nucleic acid and genotyping tubes will be labelled and placed in storage in a –20°C to -80 freezer.

Stool samples will be collected, handled and preserved according to the laboratory manual, prior to storage.

Biopsy samples (endoscopic sub-study only) will be collected from the rectum and sigmoid colon as detailed in section 12.5. From *each* location, up to 6 biopsies will be taken and distributed into storage reagents according to the laboratory manual.

The study coordinator will liaise with sites to coordinate batch shipment of samples in secure, protective packaging to the receiving lab at Addenbrooke's Hospital. On receipt, samples will be logged and stored in an approximately -80°C freezer prior to processing. Analysis will be performed using validated analytical methods in compliance with local standard operating procedures.

Unused samples will be stored for use in future approved research.

17 Statistics

17.1 Statistical methods

A statistical analysis plan will be prepared before the final database lock and will be approved by the TSC.

For peer review only - http://bmjopen.bmj.com/site/about/guidelines.xhtml

17.1.1 Primary analysis

We will report summary statistics on the primary and secondary endpoints according to treatment group. The primary analysis will consist of an estimate, 95% confidence interval and p-value of the absolute risk difference of the incidence rates of the need for medical or surgical rescue therapy within 10 days following the first administration of IV corticosteroids (primary endpoint) between the two treatment arms, using logistic regression to adjust for important baseline covariates. The primary efficacy analysis will be based upon upon the Full Analysis Population (Section 17.1.3).

The baseline characteristics will include:

- Prior diagnosis of IBD at the point of hospitalisation ('first presentation')
- Prior hospitalisation for ASUC
- Previous or current therapy with any of: immunomodulators (azathioprine, 6-mercaptopurine, 6-thioguanine, methotrexate, ciclosporin) biologics (anti-TNF-ɑ monoclonal antibodies, anti-adhesion molecule antibodies, other anti-cytokine antibodies) or oral janus kinase inhibitors
-
- Current or previous oral corticosteroid prescription(s) within 8 weeks prior to first dose of intravenous corticosteroids
- Demographics

Secondary endpoints will be compared in a similar regression model to estimate the treatment effect on an appropriate scale of comparison. This will include an assessment of the effects of treatment with anakinra on the endpoints described in Section 9.6.2.

All continuous variables will be summarised using the following descriptive statistics: n (non-missing sample size), mean, median, maximum, minimum and standard deviation. The frequency and percentages (based on the non-missing sample size) of observed levels will be reported. All summary tables will be structured with a column for each treatment in the order (Control, Anakinra) and will be annotated with the total population size relevant to that table/treatment, including any missing observations.

17.1.2 Secondary analyses

The following secondary analyses will be performed in the trial:

- Analysis of the Randomised Population (Section 17.1.3)
- Complier Average Causal Effect analysis to assess the influence of the amount of treatment received, as distinct from treatment assigned

To test the hypothesis that the clinical effects of anakinra in ASUC may differ between groups of patients with differing levels of prior inflammatory burden, pre-specified subgroup analyses, in the form of estimating treatment-covariate interactions, will be performed on the following baseline characteristics:

- Prior diagnosis of IBD at the point of hospitalisation ('first presentation')
- Prior hospitalisation for ASUC
- Naïvety to any of: immunomodulators (azathioprine, 6-mercaptopurine, 6-thioguanine, methotrexate, ciclosporin) biologics (anti-TNF-ɑ monoclonal antibodies, anti-adhesion molecule antibodies, other anti-cytokine antibodies) or oral janus kinase inhibitorsat the point of hospitalisation.
- Receipt of oral corticosteroids within 8 weeks prior to first dose of intravenous corticosteroids
- Cases of suspected or confirmed ASUC without evidence of CMV reactivation requiring treatment with anti-viral agents and without evidence of a significant GI pathogen
- Duration between 1st dose of IV corticosteroids to 1st dose of IMP.

For peer review only - http://bmjopen.bmj.com/site/about/guidelines.xhtml

17.1.3 Definition of populations

Populations to be analysed in the trial are as follows:

- Full Analysis Population: All randomised participants who receive at least one dose of IMP
- Randomised Population: All randomised participants, regardless of whether IMP was received
- Safety Population: All consenting participants

Any further populations may additionally be defined within the Statistical Analysis Plan

17.2 Interim analyses and criteria for the premature termination of the trial

Except in the case of a valid medical or safety reason, unblinding will only be performed as part of an interim analysis by a designated unblinded statistician. The unblinded statistican will be named in the IDMC charter and will provide outcome data to the iDMC. A blinded report from the iDMC will be made to the TSC and outcome data will not be shared at this stage. Final unblinding of the data to the trial statisticians and TSC will only be made after creation of a locked final analysis data set at the end of the trial.

As part of the interim analyses, the iDMC will examine the unblinded data and provide a recommendation to the TSC as to whether the trial should continue at the following two prespecified points, in addition to other timepoints detailed in the iDMC charter.

- After the feasibility stage (once the first 20 participants have completed the initial 10 days of follow up to report the primary endpoint). Assessment of feasibility will be according to the criteria set out in Section 17.2.1.
- After the first 100 participants have been recruited. Assessment for harm or futility will be according to the criteria set out in Section 17.2.2.

17.2.1 Feasibility study

After the first 20 participants have been recruited, and all completed a minimum of 10 days follow up (to the time of the primary endpoint), feasibility will be assessed. The trial will be regarded as feasible only if all of the following measures are met:

1) ≥10% of eligible participants are recruited (based upon use of screening logs to determine the percentage of eligible participants that are randomised in each site)
2) Adherence to dosing as per protocol (≥80% of participants received appropriately prescribed doses within any given 24h window)
3) Rescue therapy started on or before the 7th day following initial IV corticosteroid administration in ≥80% cases where required during the index admission (as determined by review of timing of rescue therapy administration relative to corticosteroid administration by trial investigators)
4) Maintenance of blinding: there should be no evidence of unnecessary unblinding of participants or investigators.
5) No serious safety concerns identified, especially with regard to infections or SUSARs.

17.2.2 Interim analysis

After the first 100 participants have completed Day 10 assessments, futility analysis will be performed to test the hypothesis that the reduction in absolute risk difference in the primary outcome is ≥10% using a 1-sided 2.5% significance test using logistic regression on the absolute risk reduction scale. If the hypothesis is rejected, i.e. the lower limit of a 95% 2-sided confidence interval for Treatment - Control is above -10%, then the iDMC will consider the recommendation for the study to stop early for futility. This is equivalent to spending 0.1% from the 15% total beta value set to control the

For peer review only - http://bmjopen.bmj.com/site/about/guidelines.xhtml

type 2 error under the alternative hypothesis (49% and 29% rates). If the study continues past the interim, then the final analysis will not be adjusted for the futility analysis, and standard p-values and confidence intervals will be reported.

The formal hypothesis test at the interim is non-binding and will be at the discretion of the iDMC to incorporate into the recommendation they reach. The iDMC will also review safety data, particularly relating to rescue therapy and colectomy rates and infection-related SAEs and advise the TSC accordingly.

The probability of the three possible outcomes (futile, late negative, late positive) from the interim and final analyses is shown in Figure 2, based on the true rates in the arms of the study.

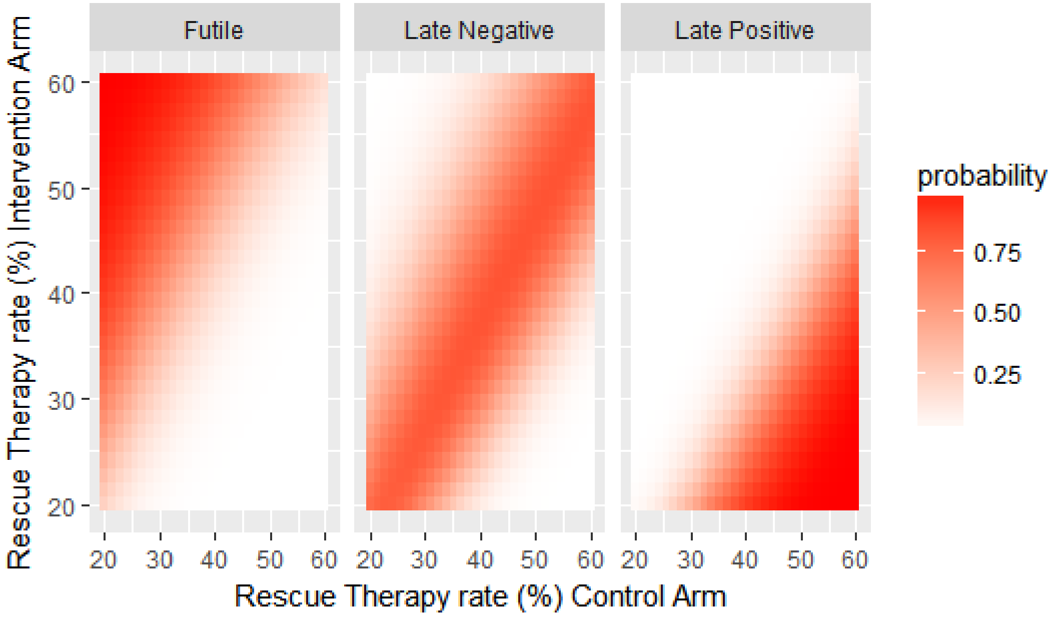

Figure 2. Probability of IASO trial outcomes

17.3 Number of Participants to be enrolled

17.3.1 Sample size calculation

We will recruit 214 patients (107 per group. This will give 85% power to detect a 20% absolute risk reduction (ARR) in the primary endpoint from a rate in the control group of 49% testing at the 5% significance level. Since the main trial observation is brief and performed in an inpatient setting, we expect negligible loss-to-follow up. The trial intervention is well tolerated and we expect low dropout rates.

We have based our estimate of the primary endpoint rate on data from the recently completed NIHR-funded CONSTRUCT trial[31]. This trial recruited 270 patients with ASUC across 52 UK sites between 2010-2013. In this trial, 49% of patients admitted with ASUC needed medical therapy (Prof J Williams, personal communication). Since there are no existing estimates of effect size for anakinra in ASUC, we chose to power IASO to detect a 20% ARR following protocol review by members of the British Society of Gastroenterology IBD clinical research group, who advised that this was the minimum effect size needed that would make a substantial difference to the patient population, and which would be broadly in line with effect sizes seen in other drugs for moderate/severe colitis[32].

For peer review only - http://bmjopen.bmj.com/site/about/guidelines.xhtml

The scientific sub-study based on testing cytokines will be performed on 40 participants. The choice of sample size is based on power studies in transcriptomic studies in UC[33] with adjustment for multiple testing, as well as pragmatic grounds of cost.

17.4 Procedure to account for missing or spurious data

Due to short and intensive period of follow up for the primary endpoint, we do not anticipate significant missing data. For the exploratory analyses of quality of life, which will require postal questionnaire responses some months after discharge, where we are unable to obtain missing data within 12 weeks of the 3- or 6-month timepoints, we will employ the standard approaches to management of missing quality of life data previously described in the CONSTRUCT trial[31].

For participants who withdraw consent to treatment, permission to continue to acquire data for outcome analysis will be sought. For those who do not consent to ongoing monitoring, including those who wish to withdraw entirely from the study, existing data acquired to the point of withdrawal will be included in the final study analysis, with missing data handled according to standard missing data methods, including missing-at-random (MAR) methods with sensitivity testing for deviation from MAR assumptions.

17.5 Definition of the end of the trial

For regulatory notification purposes, the end of trial will be the earliest of:

- The receipt of the final, returned 6 month quality of life questionnaires
- 6 months (+12 weeks) after the last participant entered the study (date of questionnaire censoring).

However, participants may enter a period of long-term follow-up via HES data examination and the trial would remain open to the Ethics Committee until the last participant's HES data capture has been completed.

18 Data handling and record keeping

18.1 CRF

All data will be transferred into a Case Report Form (CRF) which will be anonymised. All trial data in the CRF must be extracted from and be consistent with the relevant source documents. The CRFs must be completed, dated and signed by the investigator or designee in a timely manner. It remains the responsibility of the investigator for the timing, completeness, legibility and accuracy of the CRF pages. The CRF will be accessible to trial coordinators, data managers, the investigators, Clinical Trial Monitors, Auditors and Inspectors as required.

Completed originals of the CRFs should be posted, emailed or faxed to the trial coordination centre within within the timelines detailed in the CRF-completion guidelines.

The investigator will retain a copy of each completed CRF page at site. If faxed or emailed, they will retain the original. The investigator will also supply the trial coordination centre with any required, anonymised background information from the medical records as required.

The investigators must ensure that the CRFs and other trial-related documentation are sent to the trial coordination centre containing no participant identifiable data.

For peer review only - http://bmjopen.bmj.com/site/about/guidelines.xhtml

A separate form containing participant identifiable data necessary for the postage of quality of life questionnaires (name and address) and the capturing of HES data (NHS number and date of birth) will be sent to the central research team. Information contained within this form will be added to a secure database and the physical form kept in a limited-access, locked secure cabinet. Personal identifiable data kept for these purposes will be destroyed at the end of the trial.

All CRF pages must be clear, legible and completed in black ink. Any errors should be crossed with a single stroke so that the original entry can still be seen. Corrections should be inserted and the change dated and initialled by the investigator or designee. If it is not clear why the change has been made, an explanation should be written next to the change. Typing correction fluid must not be used.

18.2 Source Data

To enable peer review, monitoring, audit and/or inspection the investigator must agree to keep records of all participating participants (sufficient information to link records e.g., CRFs, hospital records and samples), all original signed informed consent forms and copies of the CRF pages.

In this trial source data include, but are not limited to:

- Patient medical records,
- Informed Consent Forms,
- Approach and Screening Logs,
- Results of clinical tests
- Questionnaires
- Worksheets for sample collection, processing, storage and shipment.

18.3 Data Protection & Participant Confidentiality

All investigators and trial site staff involved in this trial must comply with the requirements of the Data Protection Act 1998 and Trust Policy with regards to the collection, storage, processing, transfer and disclosure of personal information and will uphold the Act's core principles.

19 Data Monitoring Committee/Trial Steering Committee

Overall supervision of the trial will rest with the TSC. The TSC will be constituted in accordance with EME guidance. In particular, 66% of the TSC members will be required in order to achieve quorum; 75% of members voting must be independent; the TSC chair will be independent. A patient representative will serve as a member of the TSC. The TSC will meet in person or by teleconference with a minimum frequency of 1/year (more frequent meetings may be required at the discretion of the TSC chair) as fully detailed in the TSC Charter.

The TSC will consider recommendations from a fully independent iDMC. iDMC members and their host institutions will not be involved in the running of the trial. Meetings and running of the iDMC will be subject to a separate iDMC charter to be agreed by the iDMC chair.

The TMG will comprise investigators and individuals closely involved in the running of the trial. The TMG will meet more frequently to ensure that all practical details of the trial are progressing well.

During the trial, interim analyses of all SAEs will be provided regularly in strict confidence to the chair of the iDMC in accordance with the charter. In the light of these analyses and any other information considered relevant, the iDMC will advise the TSC

For peer review only - http://bmjopen.bmj.com/site/about/guidelines.xhtml

if, in their view, the randomised comparisons in the trial have provided both (i) "proof beyond reasonable doubt"[1] that for all, or some specific types of patients, use of anakinra is clearly indicated or clearly contraindicated; and (ii) evidence that might reasonably be expected to influence materially the patient management of many clinicians who are already aware of the results of other trials. The TSC will then decide whether to modify the trial, or to seek additional data. Unless this happens, the TSC, collaborators, trial participants and all trial staff (except those who provide the confidential analyses to the iDMC) will remain blind to the interim results on mortality and morbidity until the end of the trial.

20 Ethical & Regulatory considerations

Consent to participate in the scientific sub-study will be sought separately. Patients will be free to elect to participate in the main trial but decline the sub-study.

20.1 Ethical committee review

Before the start of the trial or implementation of any amendment we will obtain approval of the trial protocol, protocol amendments, informed consent forms and other relevant documents (e.g. GP information letters) from the REC and the HRA. All correspondence with the REC/HRA will be retained in the Trial Master File/Investigator Site File.

Annual reports will be submitted to the REC in accordance with national requirements. It is the Chief Investigator's responsibility to produce the annual reports as required.

20.2 Regulatory Compliance

The trial will not commence until a Clinical Trial Authorisation (CTA) is obtained from the MHRA. The protocol and trial conduct will comply with the Medicines for Human Use (Clinical Trials) Regulations 2004 and any relevant amendments.

Development Safety Update Reports (DSURs) will be submitted to the MHRA in accordance with national requirements. It is the Chief Investigators responsibility to produce the annual reports as required.

20.3 Protocol Amendments

Protocol amendments must be reviewed and agreement received from the Sponsor for all proposed amendments prior to submission to the HRA, REC and/or MHRA.

The only circumstance in which an amendment may be initiated prior to HRA, REC and/or MHRA approval is where the change is necessary to eliminate apparent, immediate risks to the participants (Urgent Safety Measures). In this case, accrual of new participants will be halted until the HRA, REC and/or MHRA approval has been obtained. Participating sites will be notified by email or telephone in the event of Urgent Safety Measures.

20.4 Peer Review

The proposed trial has been favourably reviewed by members of the British Society of Gastroenterology IBD Clinical Research Group.

---

1 Appropriate criteria of proof beyond reasonable doubt cannot be specified precisely, but in general a difference of at least three standard deviations in major morbidity or mortality in an interim analysis would be needed to justify halting, or modifying, the study prematurely. This criterion has the practical advantage that the exact number of interim analyses is of little importance.

For peer review only - http://bmjopen.bmj.com/site/about/guidelines.xhtml

20.5 Public and Patient Involvement

Patient involvement has been actively sought during the planning and preparation stage for this trial proposal, and will form a key part of the trial as it progresses. This is viewed as particularly important given the vital need to safeguard trial recruitment through an acceptable trial design, as well as through the provision of materials that will assist in timely recruitment under difficult conditions for the patients involved.

In December 2014, we started discussions about IASO with members of Crohn's & Colitis UK (CCUK). We invited a wide range of service users to a patient information evening, which we ran as a workshop around trial design. We subsequently established a 10-member patient steering group that has reviewed trial protocols and assisted with responses to the initial application reviews. In particular, this group have provided valuable insight into the acceptability of different dosing regimens and into ways to support patients during the consent process that has informed trial design.

The patient steering group helped to develop the trial protocol, as well as all patient-facing materials that will be used in the trial. A patient representative will serve as a member of the TSC. The patient steering group includes CCUK representatives, and will assist in the preparation and dissemination of plain language summaries of trial updates.

20.6 Declaration of Helsinki and Good Clinical Practice

The trial will be performed in accordance with the spirit and the letter of the declaration of Helsinki, the conditions and principles of Good Clinical Practice, the protocol and applicable local regulatory requirements and laws.

20.7 GCP Training

All trial staff must hold evidence of appropriate GCP training or undergo GCP training prior to undertaking any responsibilities on this trial. This training should be updated every 2 years or in accordance with your Trust's policy.

21 Sponsorship, Financial and Insurance

The trial is sponsored by Cambridge University Hospitals NHS Foundation Trust and University of Cambridge. The trial is funded as described in Table 1.

Table 1. Details of the funders of the IASO trial:

FUNDER(S)	FINANCIAL AND NON FINANCIAL SUPPORT GIVEN
EME programme (NIHR & MRC)	Financial support for all aspects of study
Swedish Orphan Biovitrum	Provision of study drug
Wellcome Trust Sanger Institute	Financial support and academic collaboration for sequencing work

Cambridge University Hospitals NHS Foundation Trust, as a member of the NHS Clinical Negligence Scheme for Trusts, will accept full financial liability for harm caused to participants in the clinical trial caused through the negligence of its employees and honorary contract holders. There are no specific arrangements for compensation should a participant be harmed through participation in the trial, but no-one has acted negligently.

The University of Cambridge will arrange insurance for negligent harm caused as a result of protocol design and for non-negligent harm arising through participation in the clinical trial.

For peer review only - http://bmjopen.bmj.com/site/about/guidelines.xhtml

22 Monitoring, Audit & Inspection

The investigator must make all trial documentation and related records available should an MHRA Inspection occur. Should a monitoring visit or audit be requested, the investigator must make the trial documentation and source data available to the Sponsor's representative. All participant data must be handled and treated confidentially.

The Sponsor's monitoring frequency will be determined by an initial risk assessment performed prior to the start of the trial. A detailed monitoring plan will be generated detailing the frequency and scope of the monitoring for the trial. Throughout the course of the trial, the risk assessment will be reviewed and the monitoring frequency adjusted as necessary.

Remote monitoring will be conducted for all participating sites. The scope and frequency of the monitoring will be determined by the risk assessment and detailed in the Monitoring Plan for the trial.

23 Protocol Compliance and Breaches of GCP

Prospective, planned deviations or waivers to the protocol are not allowed under the UK regulations on Clinical Trials and must not be used. Substantial protocol amendments would be sought from the MHRA and REC in the event of planned changes to eligibility criteria or restrictions.

Protocol deviations, non-compliances, or breaches are departures from the approved protocol. They can happen at any time, but are not planned. They must be adequately documented on the relevant forms and reported to the Chief Investigator and Sponsor immediately.

Deviations from the protocol which are found to occur constantly again and again will not be accepted and will require immediate action and could potentially be classified as a serious breach.

Any potential/suspected serious breaches of GCP must be reported immediately to the Sponsor without any delay.

24 Publications policy

Ownership of the data arising from this trial resides with the trial team. On completion of the trial the data will be analysed and tabulated and a Final Trial Report prepared.

Participating investigators do not have rights to publish any trial data.

This is a NIHR funded trial and the NIHR publishing guidelines will be followed.

Participants will be provided with a link to the study website in the participant information sheet. The website will publish a lay summary of the trial findings.

The NIHR and the Department of Health may publicise the outcome of NIHR-funded research studies through its website, in publications and in press releases where appropriate.

Authorship of final study outputs will be assigned in accordance with guidelines set out by the International Committee of Medical Journal Editors.

For peer review only - http://bmjopen.bmj.com/site/about/guidelines.xhtml

25 Appendices

25.1 Appendix 1 – Authorisation of Participating Sites

25.1.1 Required Documentation

Prior to initiating a participating site, the following documentation is required;

- PI and other key trial team staff CV (signed and dated) and GCP certificate
- Competent Authority approval (HRA, REC, MHRA)
- Local R & D capability and capacity approval
- Participating Site Agreement executed, including pharmacy participating site agreement
- Patient Information Sheets and consent forms on local headed paper
- Protocol signed and dated by PI
- Delegation of Authority Log
- Confirmation of randomisation system training

25.1.2 Procedure for initiating/opening a new site

When all the regulatory paperwork is in place, prior to site opening, an initiation meeting will take place, either face-to-face or via a teleconference. This will be led by the clinical trial coordinator or delegate with as many of the local team present as is practicable. This initiation meeting constitutes training for the trial and it is therefore imperative that all members of the trial team who will be involved in the trial are represented at the meeting. A log of attendees will be completed during the meeting. The presentation slides will be provided to the site in advance of the meeting. A trial initiation form will be completed for each site initiation meeting. Copies of all initiation documentation must be retained in the Investigator Site File (ISF) and TMF.

The sponsor's regulatory green light procedure will be followed. Following the green light, the initial supply of IMP will be ordered for shipment to the site on the authorisation of the coordinating centre coordinator. Following confirmation of receipt of the IMP at site, the site will be opened for recruitment and the randomisation system opened to that site.

25.1.3 Principal Investigator Responsibilities

The PI has overall responsibility for the conduct of the trial at the participating site.

In particular, the PI has responsibilities which include (but are not limited to):

- Ensuring the appropriate approvals are sought and obtained
- Continuing oversight of the trial
- Ensuring the trial is conducted according to the protocol
- Ensuring consent is received in accordance with the protocol and national requirements
- Ensuring that the ISF is accurately maintained
- Delegation of activities to appropriately trained staff (this must be documented on the Delegation of Authority Log)
- Providing protocol or specialised training to new members of the trial team and ensuring that if tasks are delegated, the member of staff is appropriately trained and qualified
- Appropriate attendance at the initiation meeting
- Ensuring appropriate attendance at the TSC/IDMC meetings if required and ensuring appropriate safety information is made available to the coordinating centre team in advance of the meetings

For peer review only - http://bmjopen.bmj.com/site/about/guidelines.xhtml

- Dissemination of important safety or trial-related information to all stakeholders at the participating site
- Safety reporting within the timelines and assessment of causality and expectedness of all SAEs

For peer review only

For peer review only - http://bmjopen.bmj.com/site/about/guidelines.xhtml

25.2 Appendix 2 – Safety Reporting Flow Chart

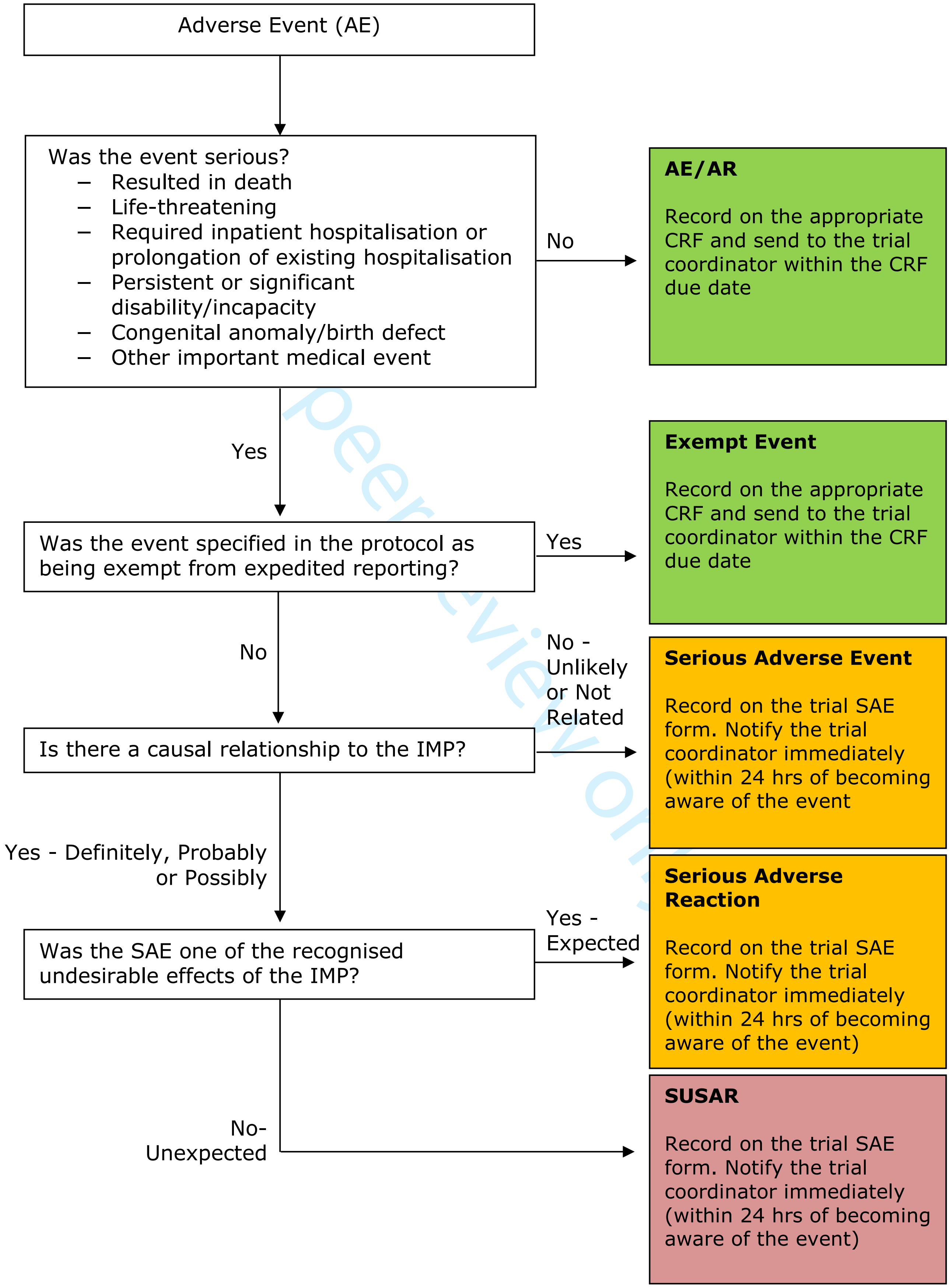

For peer review only - http://bmjopen.bmj.com/site/about/guidelines.xhtml

25.3 Appendix 3 – Amendment History

See page 2

25.4 Appendix 4 – EQ-5D and CUCQ-32 Questionnaires

EQ-5D

Under each heading, please tick the ONE box that best describes your health TODAY.

MOBILITY

I have no problems in walking about ❑
I have slight problems in walking about ❑
I have moderate problems in walking about ❑
I have severe problems in walking about ❑
I am unable to walk about ❑

SELF-CARE

I have no problems washing or dressing myself ❑
I have slight problems washing or dressing myself ❑
I have moderate problems washing or dressing myself ❑
I have severe problems washing or dressing myself ❑
I am unable to wash or dress myself ❑

USUAL ACTIVITIES *(e.g. work, study, housework, family or leisure activities)*

I have no problems doing my usual activities ❑
I have slight problems doing my usual activities ❑
I have moderate problems doing my usual activities ❑
I have severe problems doing my usual activities ❑
I am unable to do my usual activities ❑

PAIN / DISCOMFORT

I have no pain or discomfort ❑
I have slight pain or discomfort ❑
I have moderate pain or discomfort ❑
I have severe pain or discomfort ❑
I have extreme pain or discomfort ❑

ANXIETY / DEPRESSION

I am not anxious or depressed ❑
I am slightly anxious or depressed ❑
I am moderately anxious or depressed ❑
I am severely anxious or depressed ❑
I am extremely anxious or depressed ❑

For peer review only

For peer review only - http://bmjopen.bmj.com/site/about/guidelines.xhtml

UK (English) © 2009 EuroQol Group EQ-5D™ is a trade mark of the EuroQol Group

- We would like to know how good or bad your health is TODAY.
- This scale is numbered from 0 to 100.
- 100 means the best health you can imagine.
  0 means the worst health you can imagine.
- Mark an X on the scale to indicate how your health is TODAY.
- Now, please write the number you marked on the scale in the box below.

YOUR HEALTH TODAY = ☐

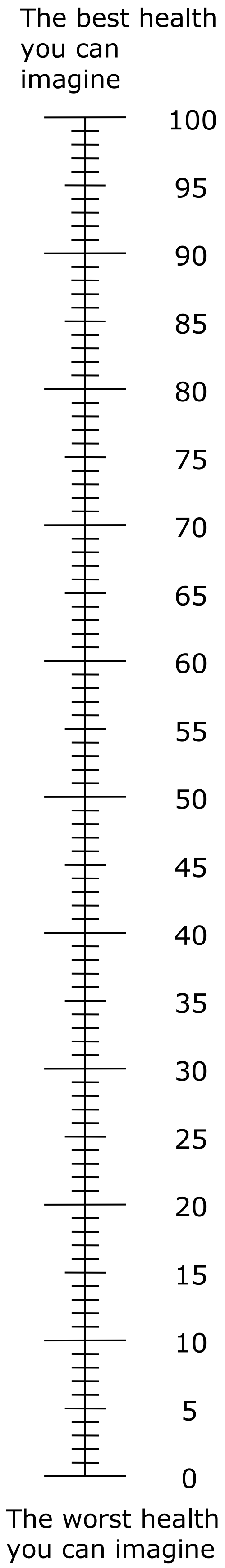

For peer review only

For peer review only - http://bmjopen.bmj.com/site/about/guidelines.xhtml

CUCQ-32

The following questions ask for your views about your bowel problem and how it has affected your life over the **last two weeks**. Please answer all the questions. If you are unsure about how to answer any question, just give the best answer you can. Do not spend too much time answering, as your first thoughts are likely to be the most accurate. If you do not wish to answer any of these questions, please leave it blank and complete the details of the question and reason(s) why it was not answered.

1. On how many days over the last two weeks have you had loose or runny bowel movements? .... days

2. On how many days in the last two weeks have you noticed blood in your stools?
.... days

3. On how many days over the last two weeks have you felt tired?
.... days

4. In the last two weeks have you felt frustrated?
a) No, not at all
b) Yes, some of the time
c) Yes, most of the time
d) Yes, all of the time

5. In the last two weeks, has your bowel condition prevented you from carrying out your work or other normal activities?
a) No, not at all
b) Yes, some of the time
c) Yes, most of the time
d) Yes, all of the time

6. On how many days over the last two weeks have you opened your bowels more than three times a day?
.... days

7. On how many days over the last two weeks have you felt full of energy?
.... days

8. In the last two weeks did your bowel condition prevent you from going out socially?
a) No, not at all
b) Yes, some of the time
c) Yes, most of the time
d) Yes, all of the time

9. On how many days over the last two weeks have your bowels opened accidentally? .... days

10. On how many days over the last two weeks have you felt generally unwell?
.... days

11. In the last two weeks have you felt the need to keep close to a toilet?
a) No, not at all

For peer review only - http://bmjopen.bmj.com/site/about/guidelines.xhtml
For peer review only

b) Yes, some of the time
c) Yes, most of the time
d) Yes, all of the time

12. In the last two weeks, has your bowel condition affected your leisure or sports activities?
a) No, not at all
b) Yes, some of the time
c) Yes, most of the time
d) Yes, all of the time

13. On how many days over the last two weeks have you felt pain in your abdomen?
……….. days

14. On how many nights over the last two weeks have you been unable to sleep well (days if you are a shift worker)?
………………… nights (or days)

15. On how many nights in the last two weeks have you had to get up to use the toilet because of your bowel condition after you have gone to bed?
………… nights

16. In the last two weeks have you felt depressed?
a) No, not at all
b) Yes, some of the time
c) Yes, most of the time
d) Yes, all of the time

17. In the last two weeks have you had to avoid attending events where there was no toilet close at hand?
a) No, not at all
b) Yes, some of the time
c) Yes, most of the time
d) Yes, all of the time

18. On how many days over the last two weeks, have you had a problem with large amounts of wind?
………………….. days

19. On how many days over the last two weeks have you felt off your food?
…………….. days

20. Many patients with bowel problems have worries about their illness. How often during the last two weeks have you felt worried?
a) No, not at all
b) Yes, some of the time
c) Yes, most of the time
d) Yes, all of the time

21. On how many days over the last two weeks has your abdomen felt bloated?
……………… days

22. In the last two weeks have you felt relaxed?

For peer review only - http://bmjopen.bmj.com/site/about/guidelines.xhtml

a) No, not at all
b) Yes, some of the time
c) Yes, most of the time
d) Yes, all of the time

23. In the last two weeks have you been embarrassed by your bowel problem?
a) No, not at all
b) Yes, some of the time
c) Yes, most of the time
d) Yes, all of the time

24. On how many days over the last two weeks have you wanted to go back to the toilet immediately after you thought you had emptied your bowels?
………………… days

25. In the last two weeks have you felt upset?
a) No, not at all
b) Yes, some of the time
c) Yes, most of the time
d) Yes, all of the time

26. On how many days over the last two weeks have you had to rush to the toilet?
………… days

27. In the last two weeks have you felt angry as a result of your bowel problem?
a) No, not at all
b) Yes, some of the time
c) Yes, most of the time
d) Yes, all of the time

28. In the last two weeks, has your sex life been affected by your bowel problem?
a) No, not at all
b) Yes, some of the time
c) Yes, most of the time
d) Yes, all of the time

29. On how many days over the last two weeks have you felt sick?
…………. days

30. In the last two weeks have you felt irritable?
a) No, not at all
b) Yes, some of the time
c) Yes, most of the time
d) Yes, all of the time

31. In the last two weeks have you felt lack of sympathy from others?
a) No, not at all
b) Yes, some of the time
c) Yes, most of the time
d) Yes, all of the time

32. In the last two weeks have you felt happy?
a) No, not at all

For peer review only - http://bmjopen.bmj.com/site/about/guidelines.xhtml

b) Yes, some of the time
c) Yes, most of the time
d) Yes, all of the time

If you did not complete any of these questions, please record the question number(s) below and, if possible, give a reason why it was not completed.

For peer review only

For peer review only - http://bmjopen.bmj.com/site/about/guidelines.xhtml

25.5 Appendix 5 – Treatment decision tree for IASO trial

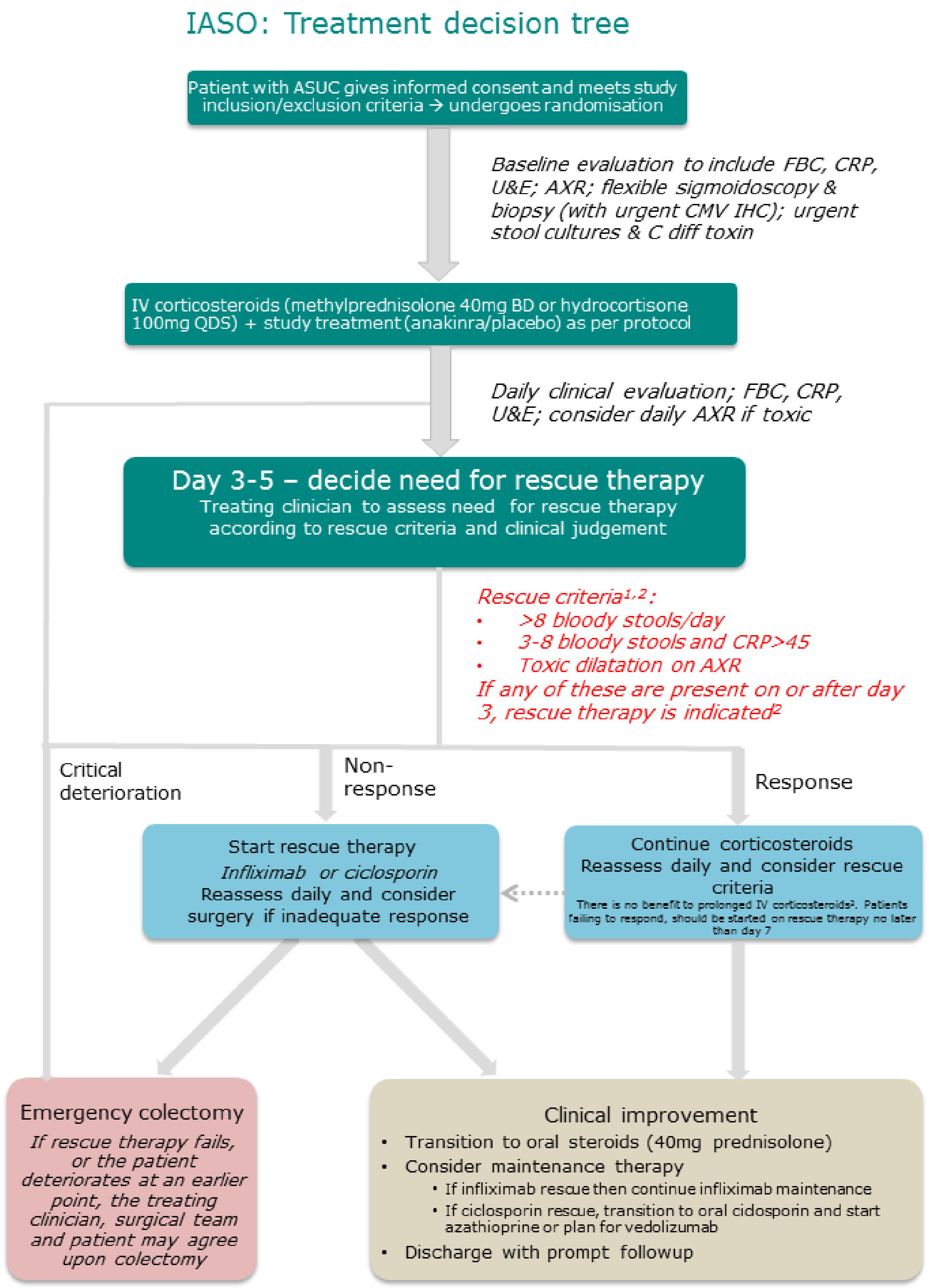

1. Travis SL *et al. Gut* 1996;**38**:905-910
2. Harbord M *et al. J Crohn's Colitis* 2017 **DOI:** https://doi.org/10.1093/ecco-jcc/jjx009

For peer review only - http://bmjopen.bmj.com/site/about/guidelines.xhtml

26 REFERENCES

1. NELA_project_team, *First patient report of the National Emergency Laparotomy Audit*. 2015, London: RCoA London.
2. Dignass, A., et al., *Second European evidence-based consensus on the diagnosis and management of ulcerative colitis part 1: definitions and diagnosis.* J Crohns Colitis, 2012. **6**(10): p. 965-90.
3. Dinarello, C.A., *Interleukin-1 in the pathogenesis and treatment of inflammatory diseases.* Blood, 2011. **117**(14): p. 3720-32.
4. Planell, N., et al., *Transcriptional analysis of the intestinal mucosa of patients with ulcerative colitis in remission reveals lasting epithelial cell alterations.* Gut, 2013. **62**(7): p. 967-76.
5. Hendel, J., et al., *A simple filter-paper technique allows detection of mucosal cytokine levels in vivo in ulcerative colitis. Interleukin-1 and interleukin-1-receptor antagonist.* Dig Dis Sci, 1996. **41**(9): p. 1775-9.
6. Ludwiczek, O., et al., *Imbalance between interleukin-1 agonists and antagonists: relationship to severity of inflammatory bowel disease.* Clin Exp Immunol, 2004. **138**(2): p. 323-9.
7. Dinarello, C.A., A. Simon, and J.W. van der Meer, *Treating inflammation by blocking interleukin-1 in a broad spectrum of diseases.* Nat Rev Drug Discov, 2012. **11**(8): p. 633-52.
8. Cominelli, F. and T.T. Pizarro, *Interleukin-1 and interleukin-1 receptor antagonist in inflammatory bowel disease.* Aliment Pharmacol Ther, 1996. **10 Suppl 2**: p. 49-53; discussion 54.
9. Opal, S.M., et al., *Confirmatory interleukin-1 receptor antagonist trial in severe sepsis: a phase III, randomized, double-blind, placebo-controlled, multicenter trial. The Interleukin-1 Receptor Antagonist Sepsis Investigator Group.* Crit Care Med, 1997. **25**(7): p. 1115-24.
10. Jiang, Y., et al., *A multicenter, double-blind, dose-ranging, randomized, placebo-controlled study of recombinant human interleukin-1 receptor antagonist in patients with rheumatoid arthritis: radiologic progression and correlation of Genant and Larsen scores.* Arthritis Rheum, 2000. **43**(5): p. 1001-9.
11. de Luca, A., et al., *IL-1 receptor blockade restores autophagy and reduces inflammation in chronic granulomatous disease in mice and in humans.* Proc Natl Acad Sci U S A, 2014. **111**(9): p. 3526-31.
12. Bersudsky, M., et al., *Non-redundant properties of IL-1alpha and IL-1beta during acute colon inflammation in mice.* Gut, 2014. **63**(4): p. 598-609.
13. Yang, B.B., S. Baughman, and J.T. Sullivan, *Pharmacokinetics of anakinra in subjects with different levels of renal function.* Clin Pharmacol Ther, 2003. **74**(1): p. 85-94.
14. Arend, W.P. and B.P. Coll, *Interaction of recombinant monocyte-derived interleukin 1 receptor antagonist with rheumatoid synovial cells.* Cytokine, 1991. **3**(5): p. 407-13.
15. Carty, E., et al., *Measurement of in vivo rectal mucosal cytokine and eicosanoid production in ulcerative colitis using filter paper.* Gut, 2000. **46**(4): p. 487-92.
16. Langereis, J.D., et al., *Steroids induce a disequilibrium of secreted interleukin-1 receptor antagonist and interleukin-1beta synthesis by human neutrophils.* Eur Respir J, 2011. **37**(2): p. 406-15.

For peer review only - http://bmjopen.bmj.com/site/about/guidelines.xhtml

17. Truelove, S.C. and L.J. Witts, *Cortisone in ulcerative colitis; final report on a therapeutic trial.* Br Med J, 1955. **2**(4947): p. 1041-8.
18. Lichtiger, S., et al., *Cyclosporine in severe ulcerative colitis refractory to steroid therapy.* N Engl J Med, 1994. **330**(26): p. 1841-5.
19. Jarnerot, G., et al., *Infliximab as rescue therapy in severe to moderately severe ulcerative colitis: a randomized, placebo-controlled study.* Gastroenterology, 2005. **128**(7): p. 1805-11.
20. Laharie, D., et al., *Ciclosporin versus infliximab in patients with severe ulcerative colitis refractory to intravenous steroids: a parallel, open-label randomised controlled trial.* Lancet, 2012. **380**(9857): p. 1909-15.
21. Irvine, E.J., *Review article: patients' fears and unmet needs in inflammatory bowel disease.* Aliment Pharmacol Ther, 2004. **20 Suppl 4**: p. 54-9.
22. Turner, D., et al., *Response to corticosteroids in severe ulcerative colitis: a systematic review of the literature and a meta-regression.* Clin Gastroenterol Hepatol, 2007. **5**(1): p. 103-10.
23. Galea, J., et al., *Reduction of inflammation after administration of interleukin-1 receptor antagonist following aneurysmal subarachnoid hemorrhage: results of the Subcutaneous Interleukin-1Ra in SAH (SCIL-SAH) study.* J Neurosurg, 2017: p. 1-9.
24. Lee, J.C., et al., *Gene expression profiling of CD8+ T cells predicts prognosis in patients with Crohn disease and ulcerative colitis.* J Clin Invest, 2011. **121**(10): p. 4170-9.
25. EuroQol, G., *EuroQol--a new facility for the measurement of health-related quality of life.* Health Policy, 1990. **16**(3): p. 199-208.
26. Alrubaiy, L., et al., *Development of a short questionnaire to assess the quality of life in Crohn's disease and ulcerative colitis.* J Crohns Colitis, 2015. **9**(1): p. 66-76.
27. Kaiser, C., et al., *Injection-site reactions upon Kineret (anakinra) administration: experiences and explanations.* Rheumatol Int, 2012. **32**(2): p. 295-9.
28. Geboes, K., et al., *A reproducible grading scale for histological assessment of inflammation in ulcerative colitis.* Gut, 2000. **47**(3): p. 404-9.
29. Marchal-Bressenot, A., et al., *Development and validation of the Nancy histological index for UC.* Gut, 2015.
30. Mosli, M.H., et al., *Development and validation of a histological index for UC.* Gut, 2015.
31. Seagrove, A.C., et al., *Randomised controlled trial. Comparison Of iNfliximab and ciclosporin in STeroid Resistant Ulcerative Colitis: Trial design and protocol (CONSTRUCT).* BMJ Open, 2014. **4**(4): p. e005091.
32. Rutgeerts, P., et al., *Infliximab for induction and maintenance therapy for ulcerative colitis.* N Engl J Med, 2005. **353**(23): p. 2462-76.
33. Boland, B.S., et al., *Validated gene expression biomarker analysis for biopsy-based clinical trials in ulcerative colitis.* Aliment Pharmacol Ther, 2014. **40**(5): p. 477-85.

For peer review only - http://bmjopen.bmj.com/site/about/guidelines.xhtml

Modified Truelove Witts Severity Index Form

Information for the IASO Trial

Subject ID: N ☐ ☐ - 1 ☐ ☐ ☐ **IASO Trial Day:** ________

Subject Date of Birth: D D - M M M - Y Y Y Y

Date of completion: D D - M M M - Y Y Y Y

Symptom Checklist (Circle the score that applies)

Category	Score
Diarrhoea (Number of daily stools)	
0–2	0
3–4	1
5–6	2
7–9	3
≥10	4
Nocturnal diarrhoea	
No	0
Yes	1
Visible blood in stool (% of movements)	
0%	0
<50%	1
≥50%	2
100%	3
Faecal incontinence	
No	0
Yes	1
Abdominal pain or cramping	
None	0
Mild	1
Moderate	2
Severe	3

Further categories are included overleaf

For peer review only

Modified Truelove Witts Severity Index Form

Category	Score
General well-being	
Perfect	0
Very good	1
Good	2
Average	3
Poor	4
Terrible	5
Abdominal tenderness	
None	0
Mild and localised	1
Mild to moderate & diffuse	2
Severe or rebound	3
Need for antidiarrhoeal drugs	
No	0
Yes	1

Total Score (Add all the circled scores together)

Total MTWSI score (between 0–21): ☐

For peer review only - http://bmjopen.bmj.com/site/about/guidelines.xhtml

SPIRIT

STANDARD PROTOCOL ITEMS: RECOMMENDATIONS FOR INTERVENTIONAL TRIALS

SPIRIT 2013 Checklist: Recommended items to address in a clinical trial protocol and related documents*

Section/item	Item No	Description	Page in Summary Manuscript (and full protocol if specified)
**Administrative information**			
Title	1	Descriptive title identifying the study design, population, interventions, and, if applicable, trial acronym	p.1
Trial registration	2a	Trial identifier and registry name. If not yet registered, name of intended registry	p.1
	2b	All items from the World Health Organization Trial Registration Data Set	p.1, 4-5, 12-13
Protocol version	3	Date and version identifier	p.2
Funding	4	Sources and types of financial, material, and other support	p.13
Roles and responsibilities	5a	Names, affiliations, and roles of protocol contributors	p.13
	5b	Name and contact information for the trial sponsor	p.13 (contact information in full protocol – Sponsor oversight carried out by Cambridge Clinical Trials Unit)

For peer review only

	5c	Role of study sponsor and funders, if any, in study design; collection, management, analysis, and interpretation of data; writing of the report; and the decision to submit the report for publication, including whether they will have ultimate authority over any of these activities	p. 12-13 (Sponsorship & Funding)<br><br>In full protocol: Sponsor oversight of adverse events (p.35), approval of amendments (p.44), monitoring (p.46).<br><br>Full sponsor review of the trial, including the protocol, was performed prior to submitting for ethics, HRA and MHRA approval.
	5d	Composition, roles, and responsibilities of the coordinating centre, steering committee, endpoint adjudication committee, data management team, and other individuals or groups overseeing the trial, if applicable (see Item 21a for data monitoring committee)	p.11 (roles of the committees)<br>Composition covered in full protocol p.43-44
**Introduction**			
Background and rationale	6a	Description of research question and justification for undertaking the trial, including summary of relevant studies (published and unpublished) examining benefits and harms for each intervention	p.2-3
	6b	Explanation for choice of comparators	p.2-3
Objectives	7	Specific objectives or hypotheses	p.3

For peer review only - http://bmjopen.bmj.com/site/about/guidelines.xhtml

Trial design	8	Description of trial design including type of trial (eg, parallel group, crossover, factorial, single group), allocation ratio, and framework (eg, superiority, equivalence, noninferiority, exploratory)	p.4

Methods: Participants, interventions, and outcomes

Study setting	9	Description of study settings (eg, community clinic, academic hospital) and list of countries where data will be collected. Reference to where list of study sites can be obtained	p.4 (information about study site locations not featured in protocol but can be viewed at http://www.isrctn.com/ISRCTN43717130)
Eligibility criteria	10	Inclusion and exclusion criteria for participants. If applicable, eligibility criteria for study centres and individuals who will perform the interventions (eg, surgeons, psychotherapists)	p.4-5
Interventions	11a	Interventions for each group with sufficient detail to allow replication, including how and when they will be administered	p.6 (extensive details covered in full protocol Section 11)
	11b	Criteria for discontinuing or modifying allocated interventions for a given trial participant (eg, drug dose change in response to harms, participant request, or improving/worsening disease)	p.6
	11c	Strategies to improve adherence to intervention protocols, and any procedures for monitoring adherence (eg, drug tablet return, laboratory tests)	p.11
	11d	Relevant concomitant care and interventions that are permitted or prohibited during the trial	p.4-6

Outcomes	12	Primary, secondary, and other outcomes, including the specific measurement variable (eg, systolic blood pressure), analysis metric (eg, change from baseline, final value, time to event), method of aggregation (eg, median, proportion), and time point for each outcome. Explanation of the clinical relevance of chosen efficacy and harm outcomes is strongly recommended	p.5-6<br>(further information in full protocol p.20-21)
Participant timeline	13	Time schedule of enrolment, interventions (including any run-ins and washouts), assessments, and visits for participants. A schematic diagram is highly recommended (see Figure)	p.4 + Figure 1
Sample size	14	Estimated number of participants needed to achieve study objectives and how it was determined, including clinical and statistical assumptions supporting any sample size calculations	p.10
Recruitment	15	Strategies for achieving adequate participant enrolment to reach target sample size	p.7 (identification of participants)<br>p.11-12 interim analyses demonstrate formal testing procedure for assessing the feasibility of the trial including recruitment

Methods: Assignment of interventions (for controlled trials)

Allocation:

Sequence generation	16a	Method of generating the allocation sequence (eg, computer-generated random numbers), and list of any factors for stratification. To reduce predictability of a random sequence, details of any planned restriction (eg, blocking) should be provided in a separate document that is unavailable to those who enrol participants or assign interventions	p.7 (stratification) Allocation sequence generation method not specified in the protocol – the randomisation and concealment lists for the trial are generated by the provider of the randomisation system (Sealed Envelope)
Allocation concealment mechanism	16b	Mechanism of implementing the allocation sequence (eg, central telephone; sequentially numbered, opaque, sealed envelopes), describing any steps to conceal the sequence until interventions are assigned	p.7
Implementation	16c	Who will generate the allocation sequence, who will enrol participants, and who will assign participants to interventions	p.7 (identification and enrolment). Full protocol (p.22) details who will be able to randomise patients<br><br>Allocation sequence generation method not specified in the protocol – the randomisation and concealment lists for the trial are generated by the provider of the randomisation system (Sealed Envelope)
Blinding (masking)	17a	Who will be blinded after assignment to interventions (eg, trial participants, care providers, outcome assessors, data analysts), and how	p.7 (full details in full protocol Section 10.4, p.22)

	17b	If blinded, circumstances under which unblinding is permissible, and procedure for revealing a participant's allocated intervention during the trial	Full protocol Section 11.4 (p.26)

Methods: Data collection, management, and analysis

Data collection methods	18a	Plans for assessment and collection of outcome, baseline, and other trial data, including any related processes to promote data quality (eg, duplicate measurements, training of assessors) and a description of study instruments (eg, questionnaires, laboratory tests) along with their reliability and validity, if known. Reference to where data collection forms can be found, if not in the protocol	p.7-9<br>p.12 (use of case report forms; location is not specified in protocol)
	18b	Plans to promote participant retention and complete follow-up, including list of any outcome data to be collected for participants who discontinue or deviate from intervention protocols	p.12 (missing data)
Data management	19	Plans for data entry, coding, security, and storage, including any related processes to promote data quality (eg, double data entry; range checks for data values). Reference to where details of data management procedures can be found, if not in the protocol	p.12 (data management)<br>More information available in full protocol Section 18 (p.42-43)

Statistical methods	20a	Statistical methods for analysing primary and secondary outcomes. Reference to where other details of the statistical analysis plan can be found, if not in the protocol	p.10-12<br>More information available in full protocol Section 17 (p.38-41)
	20b	Methods for any additional analyses (eg, subgroup and adjusted analyses)	p.11 (secondary analyses)<br>More information available in full protocol Section 17 (p.38-41)
	20c	Definition of analysis population relating to protocol non-adherence (eg, as randomised analysis), and any statistical methods to handle missing data (eg, multiple imputation)	p.10 (Patient Population)<br>p.12 (Missing Data)
**Methods: Monitoring**			
Data monitoring	21a	Composition of data monitoring committee (DMC); summary of its role and reporting structure; statement of whether it is independent from the sponsor and competing interests; and reference to where further details about its charter can be found, if not in the protocol. Alternatively, an explanation of why a DMC is not needed	p.11<br>Composition covered in full protocol p.43-44
	21b	Description of any interim analyses and stopping guidelines, including who will have access to these interim results and make the final decision to terminate the trial	p.11 (interim analyses)
Harms	22	Plans for collecting, assessing, reporting, and managing solicited and spontaneously reported adverse events and other unintended effects of trial interventions or trial conduct	p.9 (safety monitoring)<br>More information available in full protocol Section 13 (p.33-38)

Auditing	23	Frequency and procedures for auditing trial conduct, if any, and whether the process will be independent from investigators and the sponsor	Information available in full protocol Section 22 (p.46)
**Ethics and dissemination**			
Research ethics approval	24	Plans for seeking research ethics committee/institutional review board (REC/IRB) approval	p.12 (Approvals)
Protocol amendments	25	Plans for communicating important protocol modifications (eg, changes to eligibility criteria, outcomes, analyses) to relevant parties (eg, investigators, REC/IRBs, trial participants, trial registries, journals, regulators)	See full protocol Section 20.3, p.44
Consent or assent	26a	Who will obtain informed consent or assent from potential trial participants or authorised surrogates, and how (see Item 32)	p.7 (Participant Identification & Informed Consent)
	26b	Additional consent provisions for collection and use of participant data and biological specimens in ancillary studies, if applicable	This is covered in the consent form for the trial
Confidentiality	27	How personal information about potential and enrolled participants will be collected, shared, and maintained in order to protect confidentiality before, during, and after the trial	p.12 (data management) More information in full protocol Section 18.1, p.43
Declaration of interests	28	Financial and other competing interests for principal investigators for the overall trial and each study site	p.14

For peer review only

Access to data	29	Statement of who will have access to the final trial dataset, and disclosure of contractual agreements that limit such access for investigators	p.13 (data sharing)
Ancillary and post-trial care	30	Provisions, if any, for ancillary and post-trial care, and for compensation to those who suffer harm from trial participation	See full trial protocol Section 21 (p.45)
Dissemination policy	31a	Plans for investigators and sponsor to communicate trial results to participants, healthcare professionals, the public, and other relevant groups (eg, via publication, reporting in results databases, or other data sharing arrangements), including any publication restrictions	p.13 (dissemination plan)
	31b	Authorship eligibility guidelines and any intended use of professional writers	p.13 (dissemination plan)
	31c	Plans, if any, for granting public access to the full protocol, participant-level dataset, and statistical code	p.13 (dissemination plan)<br>p.13 (data sharing)
**Appendices**			
Informed consent materials	32	Model consent form and other related documentation given to participants and authorised surrogates	Separate document; not included with the trial protocol
Biological specimens	33	Plans for collection, laboratory evaluation, and storage of biological specimens for genetic or molecular analysis in the current trial and for future use in ancillary studies, if applicable	See full trial protocol (Section 16, p.38)

*It is strongly recommended that this checklist be read in conjunction with the SPIRIT 2013 Explanation & Elaboration for important clarification on the items. Amendments to the protocol should be tracked and dated. The SPIRIT checklist is copyrighted by the SPIRIT

For peer review only

Group under the Creative Commons "Attribution-NonCommercial-NoDerivs 3.0 Unported" license.

For peer review only
